# Development of a Chiral Supercritical Fluid Chromatography–Tandem Mass Spectrometry and Reversed-Phase Liquid Chromatography–Tandem Mass Spectrometry Platform for the Quantitative Metabolic Profiling of Octadecanoid Oxylipins

**DOI:** 10.1021/acs.analchem.2c02601

**Published:** 2022-10-11

**Authors:** Alessandro Quaranta, Benedikt Zöhrer, Johanna Revol-Cavalier, Kurt Benkestock, Laurence Balas, Camille Oger, Gregory S. Keyes, Åsa M. Wheelock, Thierry Durand, Jean-Marie Galano, Christopher E. Ramsden, Mats Hamberg, Craig E. Wheelock

**Affiliations:** † Unit of Integrative Metabolomics, Institute of Environmental Medicine, Karolinska Institutet, 171 77 Stockholm, Sweden; ‡ Department of Respiratory Medicine and Allergy, Karolinska University Hospital, 171 76 Stockholm, Sweden; § Respiratory Medicine Unit, K2 Department of Medicine Solna and Center for Molecular Medicine, Karolinska Institutet, 171 76 Stockholm, Sweden; ∥ Larodan Research Laboratory, Karolinska Institutet, 171 65 Stockholm, Sweden; ⊥ Waters Sweden AB, 171 65 Stockholm, Sweden; # IBMM, Univ Montpellier, CNRS, ENSCM, 34293 Montpellier, France; ∇ Laboratory of Clinical Investigation, National Institute on Aging, National Institutes of Health, 21224 Baltimore, Maryland, United States; ○ Division of Physiological Chemistry II, Department of Medical Biochemistry and Biophysics, Karolinska Institutet, 171 77 Stockholm, Sweden; ◆ Gunma University Initiative for Advanced Research (GIAR), Gunma University, Maebashi, Gunma 371-8511, Japan

## Abstract

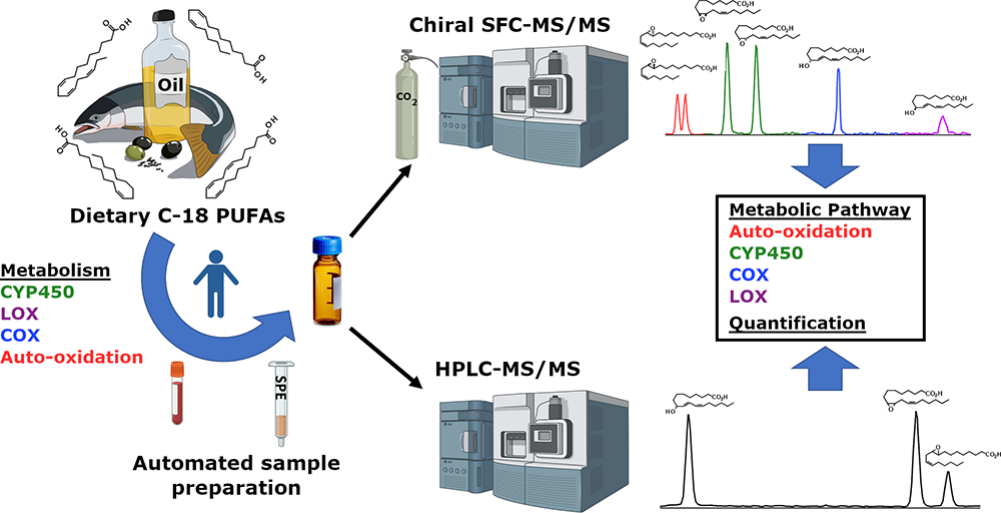

Octadecanoids are broadly defined as oxylipins (*i.e.,* lipid mediators) derived from 18-carbon fatty acids. In contrast to the well-studied eicosanoids, there is a lack of analytical methods for octadecanoids, hampering further investigations in the field. We developed an integrated workflow combining chiral separation by supercritical fluid chromatography (SFC) and reversed-phase liquid chromatography (LC) coupled to tandem mass spectrometry detection for quantification of a broad panel of octadecanoids. The platform includes 70 custom-synthesized analytical and internal standards to extend the coverage of the octadecanoid synthetic pathways. A total of 103 octadecanoids could be separated by chiral SFC and complex enantioseparations could be performed in <13 min, while the achiral LC method separated 67 octadecanoids in 13.5 min. The LC method provided a robust complementary approach with greater sensitivity relative to the SFC method. Both methods were validated in solvent and surrogate matrix in terms of linearity, lower limits of quantification (LLOQ), recovery, accuracy, precision, and matrix effects. Instrumental linearity was good for both methods (*R*^2^ > 0.995) and LLOQ ranged from 0.03 to 6.00 ng/mL for SFC and 0.01 to 1.25 ng/mL for LC. The average accuracy in the solvent and surrogate matrix ranged from 89 to 109% in SFC and from 106 to 220% in LC, whereas coefficients of variation (CV) were <14% (at medium and high concentrations) and 26% (at low concentrations). Validation in the surrogate matrix showed negligible matrix effects (<16% for all analytes), and average recoveries ranged from 71 to 83%. The combined methods provide a platform to investigate the biological activity of octadecanoids and expand our understanding of these little-studied compounds.

Oxylipins are oxygenated compounds that are formed from fatty acids by reaction(s) involving at least one step of mono- or dioxygenase-catalyzed oxygenation^1^ as well as products of autoxidation. The most studied class of oxylipins is eicosanoids, which are derived from 20-carbon polyunsaturated fatty acids via cyclooxygenase (COX), lipoxygenase (LOX), and cytochrome P450 (CYP) activity. However, 18-carbon fatty acids including oleic, linoleic, α-linolenic, γ-linolenic, and stearidonic acid can also be metabolized by these same enzymes^2,3^ as well as undergo radical-mediated reactions and autoxidation, resulting in a complex combination of structurally heterogeneous 18-carbon oxylipins, collectively defined as octadecanoids (Figure 1). While eicosanoids regulate a diverse set of homeostatic and inflammatory processes linked to numerous diseases,^4^ much less is known about the biological activity of octadecanoids. Recently, interest in octadecanoids is increasing, with studies showing their involvement in itch and pain modulation,^5^ thermogenesis and fatty acid uptake in the skeletal muscle and adipose tissue,^6,7^ protection against fat-induced obesity,^8^ regulation of intestinal inflammation and insulin resistance,^8^ proliferation of cancer cells,^9^ impediment of immune tolerance,^10^ and correct formation of the skin water barrier.^11^

**Figure 1:**
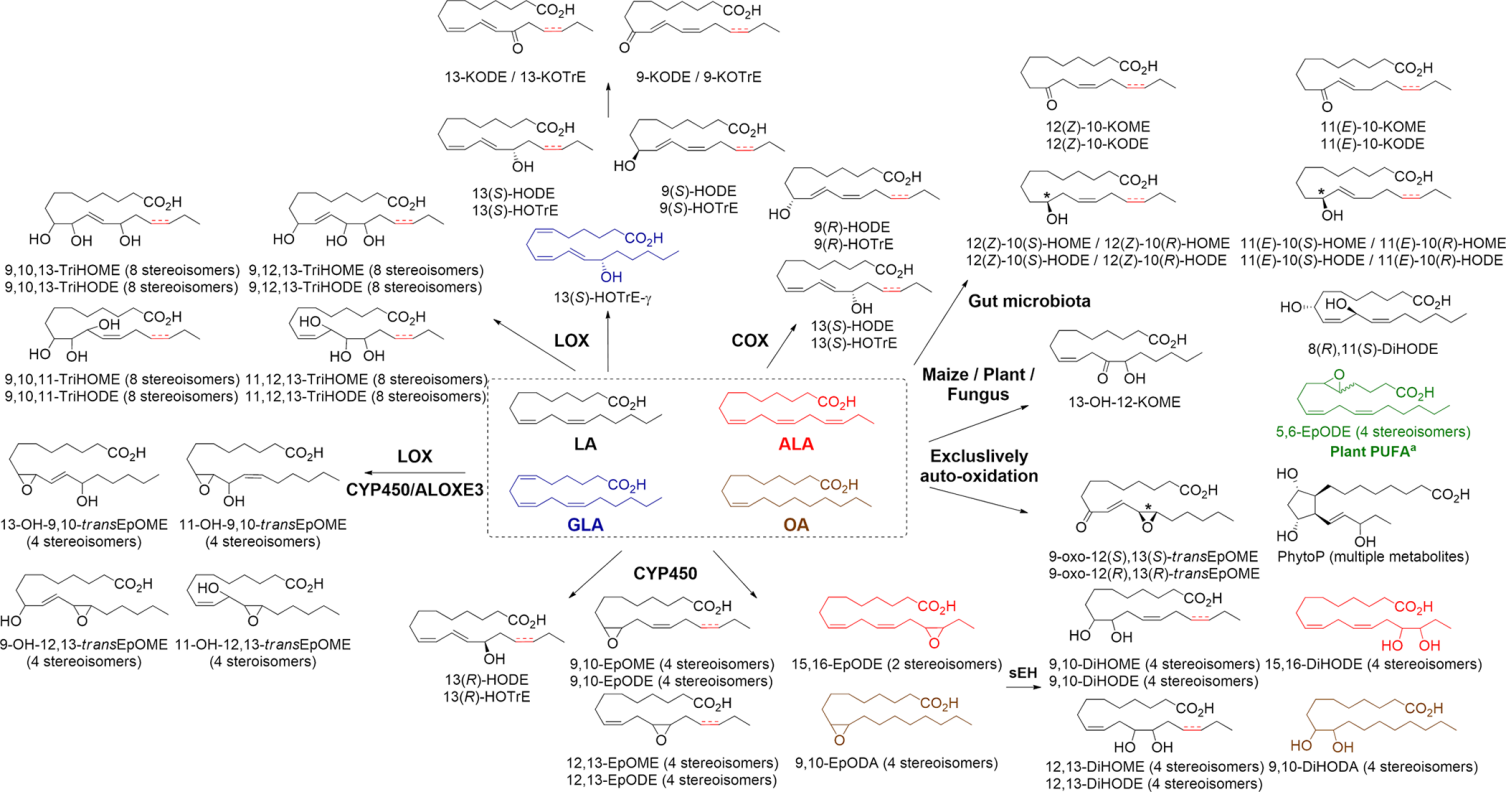
Synthetic pathway for octadecanoid compounds included in the developed analytical platform. LA = linoleic acid, ALA = α-linolenic acid, GLA = γ-linolenic acid, and OA = oleic acid. The fatty acid substrate is indicated by color. See Table S1 for a list of octadecanoid nomenclature. Superscript a indicates that the 5,6-EpODEs (colored green) are derived from pinolenic acid (*cis*,*cis*,*cis*-5,9,12) and columbinic acid (*trans*,*cis*,*cis*-5,9,12), which are isomers of GLA. The asterisk indicates that both enantiomers are included in the SFC method based upon the availability of enantiopure standards. In IUPAC nomenclature, the term "oxo" is used to indicate an "=O" group bonded to the corresponding numbered carbon; however , in this work the colloquial "keto" is used (e.g., 9-keto or 9-KOTrE *vs.* 9-OxoOTrE).

Enzymatic biosynthesis of octadecanoids results in enzyme-dependent stereospecific oxidation,^12^ whereas autoxidation produces racemic mixtures.^13^ Determining chirality can therefore be useful in establishing the synthetic route as well as the function.^14^ For example, enantiomers can interact in divergent ways with receptors, resulting in varying biological effects.^15^ Chiral separation of oxylipins can be performed by normal-phase liquid chromatography (NPLC), with excellent resolution and enantioselectivity. However, due to poor compatibility of NPLC solvents with electrospray ionization (ESI), chiral reversed-phase LC (RPLC) separations are preferred.^16^ Chiral RPLC methods can provide similar levels of resolution but generally require long analysis and equilibration times.^17,18^ The new generation of chiral columns, with sub-2 μm particles and higher tolerance to a range of solvents, have significantly improved chiral separation.^19^ Recently, supercritical fluid chromatography (SFC) has gained popularity^20^ due to improved coupling to the mass spectrometer and introduction of robust instrumentation.^21^ SFC employs supercritical CO_2_ as the primary eluent which, due to low viscosity and high diffusivity, enables the use of high flow rates with small particle columns. Supercritical CO_2_ is equivalent to hexane in polarity but is highly miscible with most organic solvents, enabling the use of modifiers such as MeOH and acetonitrile,^22^ making it a good option for chiral chromatography. Rapid achiral methods for oxylipin quantification by SFC have been recently developed;^23,24^ however, application for chiral characterization has not yet been reported.

We developed methods for both the chiral and achiral quantification of octadecanoids (Figure S1). Chiral-SFC was able to resolve complex configurations including combinations of regio- and stereoisomers in epoxides and diols as well as structures with multiple chiral centers (*e.g.,* triols) in reasonable time frames. The achiral LC method maintained resolution of regioisomers while enabling more sensitive quantification. The methods can be used separately or sequentially to obtain a complete characterization of the octadecanoids. This platform provides the broadest panel to date to quantify these little-studied compounds, enabling exploration of their putative role(s) in disease processes and expanding the field of octadecanoid research.

## Experimental Section

### Chemicals and Reagents

Commercially available octadecanoid standards were purchased from Cayman Chemical (Ann Arbor, MI, USA) or from Larodan AB (Solna, Sweden). All other standards were custom-synthesized, with details provided in the Supporting Information Section III. A full list of the octadecanoids included in the method as well as their source and nomenclature is reported in Table S1. MS-grade methanol (MeOH), ethanol (EtOH), acetonitrile (ACN), isopropanol (IPA), acetic acid, and ammonium acetate were acquired from Fisher Scientific (Waltham, MA, USA). Water was produced through a Milli-Q system (Millipore, Bedford, MA, USA). Food-grade carbon dioxide (CO_2_, purity: ≥99.7%) was purchased from Strandmöllen AB (Ljungby, Sweden). Human reference plasma was acquired from Seralab (Haywards Heat, UK) and stored at −80 °C until use. It had the following reported characteristics: origin, USA; sex, female; code, PK2F-123-F-28425; batch no., T2012313.

### Solid-Phase Extraction (SPE) Method Development

Prior to extraction, an internal standard (IS) solution consisting of a mix of 10 isotopically labeled octadecanoids and one non-labeled IS for phytoprostanes and phytofurans (PhytoP/Fs) was added (Table S1; Figure S2). The IS solution was prepared at individual concentrations of 100–200 ng/mL, adjusted to have a signal intensity of ∼10% of the highest calibration point. Octadecanoids were extracted using an Extrahera automated sample preparation system (Biotage, Uppsala, Sweden). The automated workflow enabled the parallel processing of 48 samples in 90 min. The SPE protocol was adapted from a previously published method,^14^ with minor modifications. Briefly, 250 μL of plasma was diluted to 1 mL with extraction solution (citric acid 0.1 M/Na_2_HPO_4_ 0.2 M, pH 5.6) and loaded onto preconditioned 3 mL (3 cc/60 mg) Waters Oasis HLB cartridges (Milford, MA, USA). Samples were washed 3 × 3 mL with water and a fourth time with 3 mL MeOH:H_2_O (1:9). Octadecanoids were eluted with 2.5 mL of MeOH, and the eluates were dried under a gentle N_2_ stream. Dried samples were reconstituted in 80 μL of MeOH, filtered through a 0.1 μm polyvinylidene fluoride membrane spin filter (Amicon, Merck Millipore Cooperation, Billerica, MA, USA), and transferred to LC vials for analysis.

To ensure compatibility of sample solvent composition between SFC and LC, 10 μL of water was added to all samples between SFC and LC analysis. Water is not compatible with the stationary phase of the chiral column used in the SFC separation but is fundamental to ensure a Gaussian peak shape for the more polar compounds in the RPLC separation. The final sample solvent composition was 100% MeOH for SFC and MeOH:H_2_O (85:15) for LC. An overview of the full analytical workflow is presented in Figure S1.

### SFC-MS/MS Method Development

The SFC method was developed on a Waters UPC^2^ system, equipped with a binary solvent manager delivery pump, a sample manager, a two-position column oven, an active backpressure regulator (ABPR), and an isocratic pump for the delivery of the make-up solvent. The system was coupled to a Waters Xevo TQ-XS mass spectrometer via the commercial interface kit (Waters), consisting of two T-junctions allowing back-pressure control and post-column infusion of the make-up solvent. Chiral separation was performed on a Waters Trefoil AMY1 column (3.0 × 150 mm, 2.5 μm). In order to stabilize the secondary structure of the amylose helices for an increased selectivity of the stationary phase, the column was conditioned and activated with 5000 mL of CO_2_:MeOH, CH_3_COONH_4_ (5 mM) (1:1) and 70 mL of ACN:IPA (1:1), and HCOOH 0.2% v/v before use. A detailed procedure for the stationary phase activation is reported in the Supporting Information.

Different compositions of the mobile phase and make-up solvent were tested to maximize the resolution between the highest possible number of octadecanoid diastereoisomers and the MS signal. The final method employed MeOH:EtOH (8:2) and CH_3_COOH 0.1% v/v as mobile phase B (mobile phase A being supercritical CO_2_) and MeOH and CH_3_COONH_4_ (5 mM) as the make-up solvent. The gradient (Table S2) started with 5% B, which was maintained until 1 min and then increased linearly to 25% at 11 min, and to 30% at 12.3 min. The column was then washed in 50% B for 2.5 min and re-equilibrated under the initial condition for 2.2 min. The flow rate was 2.0 mL/min during separation and equilibration but was decreased to 1.5 mL/min during the wash to avoid system overpressure. The chiral separation was achieved in 12.5 min, and the total analysis time was 18 min including injection. The column temperature was maintained at 35 °C, and the ABPR was set at 2000 psi (isobaric conditions over the gradient). The make-up solvent was delivered at a constant flow rate of 0.2 mL/min, the sample manager temperature was set to 8 °C, and the injection volume was set to 2 μL.

The MS source was operated in negative-ion ESI mode under the following conditions: capillary voltage: 2.4 kV, source offset: 30.0 V, source temperature: 150 °C, desolvation temperature: 600 °C, cone gas flow: 150 L/h, desolvation gas flow: 1000 L/h, and nebulizer gas pressure: 7.0 bar. MS analyses were performed in negative MRM mode, with the collision energy, cone voltage, and dwell time manually optimized for each octadecanoid. One transition per analyte was selected based upon sensitivity and selectivity (Table S5).

### LC–MS/MS Method Development

LC–MS/MS analyses were performed on a Waters Acquity UPLC system coupled to a Xevo-TQ-XS mass spectrometer. An Acquity BEH C_18_ column (2.1 mm × 150 mm, 1.7 μm, Waters, Milford, MA, USA) was used for the separation, with mobile phase A consisting of Milli-Q water and CH_3_COOH 0.1% v/v and mobile phase B consisting of ACN:IPA (9:1). The column temperature was set to 60 °C, the autosampler temperature was maintained at 8 °C, the injection volume was set to 5 μL, and the flow rate was kept at 0.45 mL/min. Gradient elution (Table S7) was performed starting from 35% B, which was linearly increased to 40% at 2.1 min, to 42% at 3.5 min, to 50% at 5 min, to 65% at 11.5 min, to 72.5% at 13 min, and to 80% at 15 min. The column was then washed in 100% B for 2 min and re-equilibrated under initial conditions for 2 min. The resulting separation time was 13.5 min, for a total run time of 20 min including injection. MS analyses were performed in negative MRM mode, with the collision energy, cone voltage, and dwell time manually optimized for each octadecanoid. One transition per analyte was selected based upon sensitivity and selectivity (Table S8).

### Method Validation

Both methods were validated following the EMA ICH guidelines with the following exceptions: only three QC levels were used for the determination of recovery, accuracy, and precision; stability was only evaluated in the autosampler. Validation was performed in terms of sensitivity (lower limit of quantification, LLOQ), linearity, inter- and intra-day accuracy and precision, recovery, matrix effects, and autosampler analyte stability. Due to the challenge of obtaining a blank matrix, validation experiments were performed using a surrogate matrix, consisting of 100 mM phosphate buffer saline (PBS) at a pH of 7.2 and 40 g/L of fatty acid-free bovine serum albumin (BSA, Sigma-Aldrich, St. Louis, MO, USA). Accuracy and precision were evaluated both in the surrogate matrix for the whole analytical procedure and in the solvent to determine instrumental values. Recovery and matrix effects for the IS were evaluated both in the surrogate matrix and in the human plasma reference material. A complete description of the parameters evaluated during the validation of the two described methods and of the criteria employed in the process is reported in the Supporting Information, Section VII.

### Analysis of Octadecanoids in Human Plasma

The applicability of the platform for the analysis of plasma was evaluated by replicate extraction and injection of the spiked plasma reference material. Commercial pooled human plasma was spiked to contain all target octadecanoids at concentrations ranging 0.75–18.75 ng/mL, extracted 12 times, and injected at regular intervals during a study sequence consisting of 151 plasma samples for a total 72 h of run time. Octadecanoid concentrations in the reference plasma were monitored to evaluate the precision of the analytical workflow as well as the agreement between concentrations obtained by the two methods.

## Results and Discussion

### Chiral SFC Method Development

Initial efforts examined the Waters AMY-1 and CEL-2 columns, with the AMY-1 column evidencing superior performance for separating the octadecanoids, in agreement with earlier studies.^17,19^ The AMY-1 column employs a tris(3,5-dimethylphenylcarbamate) derivative of amylose as the stationary phase. The separation of sugar polymer-based chiral columns is dependent on the secondary structure of the sugar polymer and on the nature of the chemical substituents. These factors are connected by the stabilizing action of the substituents on the amylose helices through electrostatic interactions and hydrogen bonds.^25^ Small molecules in the mobile phase can interact via the same mechanisms and interfere with the secondary structure of the stationary phase, affecting the column selectivity. It was observed that AMY-1 columns conditioned according to the manufacturer instructions evidenced poor resolution and selectivity toward octadecanoid stereoisomers (Figures S3A*vs*Figure S3D). However, after testing multiple solvent and additive combinations, we found that we could permanently alter the column selectivity for octadecanoids. The optimal effect was obtained by flushing 5 mM CH_3_COONH_4_ in MeOH in combination with supercritical CO_2_, which improved the resolution for less polar species and increased the retention for all octadecanoids, and ACN:IPA (1:1) and acetic acid (0.2% v/v), which affected the more polar analytes (*e.g.,* diols and triols). The column preparation procedure is described in the Supporting Information and in Figure S3. This conditioning was vital to achieve the necessary selectivity and was stable over the column life span (tested on ∼1500 injections).

**Figure 2:**
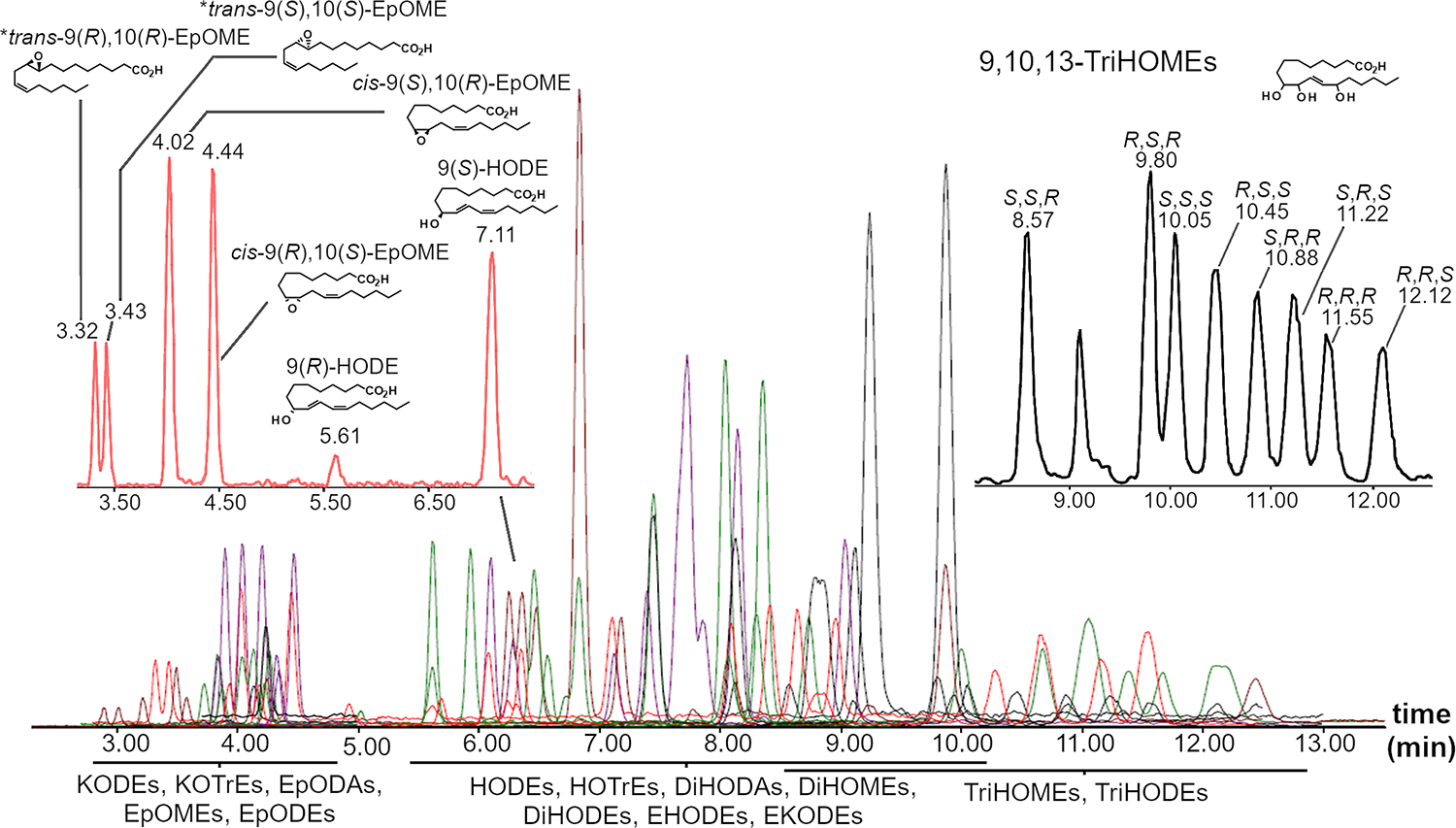
Overlaid chromatogram of all the acquired MRM transitions for the SFC octadecanoid method. The panels show the chiral separation of *cis-*/*trans-*9,10-EpOMEs (four diastereoisomers), (*R*),(*S*)-HODEs, and 9,10,13-TriHOMEs (eight diastereoisomers). The asterisk indicates that the elution order is not confirmed by an analytical standard and is instead inferred by comparison with analogous compounds.

The choice of the mobile phase composition was based on the ability to resolve the highest number of octadecanoid isomers in all the targeted chemical classes (*e.g.,* epoxides, mono-hydroxides, epoxy-alcohols (EHODEs), diols, and triols). MeOH was chosen as the main solvent because it has the highest polarity among CO_2_-compatible solvents, provided overall good separation for all octadecanoid classes, and was able to resolve the largest number of TriHOME diastereoisomers. Binary mixtures of MeOH with other solvents were used to evaluate the effect on the various classes of analytes and ternary mixtures were tested to fine-tune the separation (Figure S4 and Table S3). The best overall results were obtained with the ternary mixture MeOH:ACN:IPA (8:1:1, acetic acid 0.1% v/v) and with MeOH:EtOH (8:2, acetic acid 0.1% v/v). While the former still maintained the good diol separation provided by ACN without strongly affecting the other classes of analytes, the latter was chosen given the overall better performance for more polar compounds, the higher total number of resolved species, and the lower complexity of preparation. Under these conditions, summarized in Table S2, a total of 103 octadecanoids were separated (Figure 2 and Tables S1 and S5).

All analytes possessing a single chiral center could be readily resolved into the two enantiomers. For monohydroxides, it was observed that the (*R*)-enantiomer eluted earlier than the (*S*)-enantiomer, as previously reported for this stationary phase.^19^ This order of elution was inferred for analogous compounds for which enantiopure standards were not available. For compounds possessing more than one chiral center, assignment was possible only with enantiopure standards. Otherwise, peaks were reported with a sequential number indicating elution order. Enantiopure standards could be obtained for *cis-*epoxides and *threo-*diols of LA (*cis-*9,10-EpOME and *cis-*12,13-EpOME; *threo*-9,10-DiHOME and *threo*-12,13-DiHOME), as well as for *cis*-9,10-EpODA. For oxidation in the 9,10-position, the order of elution was conserved, with the 9(*R*),10(*S*)-enantiomer eluting after the 9(*S*),10(*R*), whereas 12,13-EpOME had the opposite elution order. Accordingly, the same elution order was assumed for the *cis*-9,10-EpODEs. The order of elution of the *threo-*9,10-and *threo-*12,13-DiHOME enantiomers reflected that of the monohydroxyls, with the (*R*),(*R*) enantiomers always eluting before the (*S*),(*S*). This order of elution was assumed for *threo*-diols from other parent fatty acids for cases in which they were chromatographically resolved. Assignment of chiral configuration is of utility when assessing biological activity and biosynthetic origin. For example, 9(*R*)- and 13(*S*)-HODE are synthesized as pure enantiomers by COX and LOX, respectively but are produced as racemates by autoxidation. The two enantiomers are generally reported as a single compound but may exert divergent biological activity. Cabral et al.^9^ showed the opposite effects of 9- and 13-HODE enantiomers on the proliferation of Caco-2 cells in colorectal cancer, with the (*S*)-enantiomer having a pro-apoptotic and antiproliferative effect and the (*R*)-enantiomer increasing cell growth and DNA synthesis. More complex enantioseparations were also possible, with all 9,10,13-TriHOME diastereoisomers separated in <13 min (Figure 2). TriHOMEs are the end product of multiple biosynthetic pathways, and their chiral determination can be useful for interpretating their origin (*e.g*., 15-LOX in eosinophils produces 9,10,13(*S*)-TriHOMEs and eLOX3 synthesizes 9(*R*),10,13(*R*)-TriHOMEs in the skin^11,26^).

Linoleic acid epoxides and diols are among the most studied octadecanoids to date and are involved in multiple biological processes including inducing pulmonary edema^27^ and impeding immune tolerance.^10^ Linoleic acid epoxides are produced by CYP epoxidation in *cis* and by autoxidation in both *cis* and *trans* configurations. The epoxide rings can subsequently be opened by the soluble epoxide hydrolase to produce the corresponding vicinal diols with retained configuration (*threo* from *cis* and *erythro* from *trans*).^28^ The method was able to fully resolve the four peaks of 9,10-EpOME (two *cis*- and two *trans*-enantiomers) and 9,10-DiHOME (two *threo*- and two *erythro*-enantiomers) but resolved only two peaks for 12,13-DiHOME. In general, improved separation was always achieved for compounds with oxidation at C-5/C-6 and C-9/C-10 compared to compounds oxidized at C-12/C-13 and C-15/C-16 (Figure S5). The systematic nature of this observation points to a better selectivity of this stationary phase for compounds where the oxidation site is closer to the carboxylic moiety compared to analytes with polar groups at both ends of the carbon chain. To confirm the involvement of the stationary phase, an independent method was developed on a Waters Trefoil CEL-2 column and used to separate the eight diastereoisomers of 9,12,13-TriHOME (Table S4 and Figure S6), providing an alternative method if full chiral resolution of these compounds is necessary.

Octadecanoid quantification was performed at two levels of confidence depending on the availability of analytical standards. For the 68 species for which pure standards could be obtained, 10-point calibration curves were prepared, covering a concentration range of 0.06–1000 ng/mL. The linear range designed for the calibration curves considered the sensitivity, MRM noise, and ESI efficiency for the various analytes. Thus, the number of calibration levels was not consistent, but a minimum of six points was ensured for all compounds. Twenty-six additional species were obtained in enriched solutions, which were used to optimize the MRM transitions and acquire the retention times. These compounds were quantified on the calibration curve of the structurally closest species (enantiomers, diastereoisomers, or regioisomers). An additional nine species were screened for but not quantified (Table S5). The linear ranges, equations, and correlation coefficients obtained for both solvent- and matrix-matched calibration curves are reported in Table S6.

### Achiral LC Method Development

The RPLC–MS/MS method was developed in parallel with the chiral SFC-MS/MS platform, to provide a complementary approach in terms of sensitivity and robustness as well as to analyze compounds produced exclusively by autoxidation. The LC separation was adapted from our previous method^14^ and modified to account for the different polarity of the target analytes. A total of 67 species were included in the method, of which 62 could be obtained as isolated pure standards. PhytoPs and PhytoFs were only included in the LC method because they possess multiple chiral centers in combination with multiple double bond configurations, the resolution of which would lead to complex chromatography.^29^ Additionally, because PhytoPs and PhytoFs are produced by autoxidation (Figure S2), the ensuing signal splitting of the racemates would result in sensitivity decrease.

The final method could resolve all the key isomeric features, including isobaric PhytoPs, *cis-* and *trans-*epoxides, *threo-* and *erythro-*diols, and (*E*)/(*Z*) double bond configurations (Figure S7). Although epoxide and diol regioisomers can be easily resolved by RPLC, they are not commonly included in oxylipin quantification methods.^30^ Given the different formation routes, with *cis*-epoxides and *threo*-diols formed enzymatically and *trans*-epoxides and *erythro*-diols by autoxidation,^28^ their discrimination and characterization could provide useful insight into the biosynthesis of both classes of isomers and a more precise assessment of bioactivity. Octadecanoids were quantified on 11-point calibration curves ranging from 0.01 to 1000 ng/mL. The linear ranges, equations, and correlation coefficients obtained for both solvent- and matrix-matched calibration curves are reported in Table S9.

### Modification to the SPE Procedure

Due to the manufacturer’s recommendation not to use water with the Trefoil column, the SPE procedure was modified by additional aqueous wash steps to minimize the breakthrough of polar species, which could precipitate in the beginning of the SFC gradient under low MeOH conditions. The addition of three extra aqueous wash steps ensured the quantitative removal of phosphates from eluates (Table S11 and Figure S8). For a detailed discussion of the results, see Section VI of the Supporting Information.

### Method Validation

Both methods were validated for linearity, sensitivity, inter- and intra-day accuracy and precision, matrix effect, recovery, and autosampler stability. Inter- and intra-day accuracy and precision were determined both in the solvent and in surrogate matrix after SPE extraction at three concentration levels. The former was used to evaluate the instrumental accuracy and precision and is a common approach when analyte-free matrices are not available,^14,31^ while the latter was employed to assess accuracy and precision of the complete procedure. Average figures of the validation and number of species exceeding the thresholds for each parameter are reported in Table 1.

**Table 1:** Metrics for Validation of the LC and SFC Methods at Three Different Concentrations^g^

		method metrics	SFC (*n* = 68)	LC (*n* = 62)		
		linearity (*R*^2^): solvent-based validation^a^	0.995 (*n* = 0)	0.995 (*n* = 0)		
		linearity (*R*^2^): matrix-based validation^a^	0.995 (*n* = 0)	0.995 (*n* = 0)		
		intra-day accuracy,^b^ deviation in %				
		low – solvent	107% (*n* = 4)	152% (*n* = 9)		
		low – matrix	100% (*n* = 7)	183% (*n* = 38)		
		medium – solvent	103% (*n* = 0)	118% (*n* = 9)		
		medium – matrix	89% (*n* = 29)	125% (*n* = 46)		
		high – solvent	109% (*n* = 13)	106% (*n* = 3)		
		high – matrix	90% (*n* = 25)	107% (*n* = 48)		
		inter-day accuracy,^b^ deviation in %				
		low – solvent	104% (*n =* 1)	126% (*n =* 7)		
		low – matrix	104% (*n =* 7)	220% (*n =* 35)		
		medium – solvent	102% (*n =* 0)	112% *(n =* 7)		
		medium – matrix	94% (*n =* 16)	126% (*n =* 47)		
		high – solvent	108% (*n =* 2)	106% (*n =* 1)		
		high – matrix	96% (*n =* 16)	106% (*n =* 44)		
		intra-day precision,^c^ RSD in %				
		low – solvent	12% (*n =* 0)	7% (*n =* 0)		
		low – matrix	25% (*n =* 13)	9% (*n =* 0)		
		medium – solvent	3% (*n =* 0)	3% (*n =* 0)		
		medium – matrix	9% (*n =* 4)	6% (*n =* 2)		
		high – solvent	2% (*n =* 0)	2% (*n =* 0)		
		high – matrix	6% (*n =* 0)	5% (*n =* 2)		
		inter-day precision,^c^ RSD in %				
		low – solvent	10% (*n =* 0)	13% (*n =* 5)		
		low – matrix	26% (*n =* 14)	14% (*n =* 3)		
		medium – solvent	4% (*n =* 0)	6% (*n =* 1)		
		medium – matrix	12% (*n =* 10)	8% (*n =* 3)		
		high – solvent	3% (*n =* 0)	2% (*n =* 0)		
		high – matrix	10% (*n =* 8)	7% (*n =* 2)		
		recovery^d^ value in %				
		low	74% (*n =* 4)	83% (*n =* 1)		
		medium	80% (*n =* 3)	75% (*n =* 4)		
		high	77% (*n =* 3)	71% (*n =* 4)		
		internal standard (plasma)	88% (*n =* 0)	77% (*n =* 0)		
		matrix effect; surrogate matrix,^e^ slope relative error in %	8% (*n =* 9)	8% (*n =* 8)		
		matrix effect; surrogate matrix,^f^ internal standards relative error in %	45% (*n =* 9)	–8% (*n =* 1)		
		matrix effect; plasma,^f^ internal standards relative error in %	39% (*n =* 9)	–35% (*n =* 10)		

a Average *R*^2^ value obtained by the average of three replicate calibration curves. The number of species with *R*^2^ < 0.995 is reported in parentheses.

b Accuracy reported as average percentage deviation from the theoretical concentration for all octadecanoids at three concentration levels. The number of compounds with deviation >15% (deviation >30% at low concentration) is reported in parentheses.

c Precision reported as average percentage RSD for all octadecanoids at three concentration levels. The number of species with RSD >15% (30% at low concentration) is reported in parentheses.

d Recovery values reported as average for all octadecanoids at three concentration levels. The number of species with recovery <40% is reported in parentheses.

e Matrix effects in the surrogate matrix reported as the average relative error between the slope of the calibration curve spiked in surrogate matrix extracts and the calibration curve spiked in the solvent. The number of species with matrix effect >15% is reported in parentheses.

f Matrix effects for the internal standards (*n* = 9 in SFC and *n* = 10 in LC) in the surrogate matrix and plasma are reported as average relative error between the signal (peak area) obtained by spiking the IS solution in matrix extracts (*n* = 6) and the signal obtained by preparing the same solution in solvent (MeOH). The number of species with matrix effects >15% is reported in parentheses.

g RSD, relative standard deviation.

The linearity and LLOQ for each method were evaluated on three solvent-matched calibration curves injected in three consecutive days. The LLOQ were generally higher for SFC (Table S6, 0.06–15.00 ng/mL) compared to LC (Table S9, 0.01–1.25 ng/mL) due to two primary factors: the lower injection volume in SFC and the average lower flow rate to the ESI source used in LC. Good linearity over the investigated linear ranges was obtained for all octadecanoids in both methods, with *R*^2^ coefficients always >0.995. Carryover was evaluated for both methods by injection of two consecutive solvent blanks after the most concentrated calibration level and was <5% for all analytes in the LC method and below detection in the SFC method (data not shown). The carryover of internal standards was evaluated by injection of a solvent blank after the lowest calibration level in both techniques. For both methods, no signal from the internal standards was detected.

Instrumental accuracy showed good results at all tested concentration levels in both methods, with only a few compounds exceeding the acceptable limits in the LC method. Notably, 9- and 13-HODE, 11(*E*)-10-KOME, 11(*E*)-10-HOME, and 9-OH-12,13-EpOME had high deviation from the theoretical concentration value at both low and medium concentrations (300–800%, Table S13). These observed deviations were due to issues associated with high background levels affecting quantification at the lower end of the calibration curve, discussed in detail in Section VII of the Supporting Information. If these compounds were excluded, then the average accuracy was 107 and 106% at low concentrations (intra- and inter-day, respectively) and 108 and 106% (intra- and inter-day, respectively) at medium concentrations.

When accuracy was determined for the full analytical procedure, average deviations were still within the acceptable limits; however, a large number of analytes exceeded the thresholds of ±30% (low concentration) and ± 15% (medium and high concentrations; Table 1 for average values; Tables S12 and S13 for single analytes in the SFC and LC platform, respectively). This could be due to the low number of IS available, resulting in poor average correction for recovery and matrix effects. Instrument and method precision were appropriate for both methods at all concentration levels, indicating that relative variations are accurately reproduced. This is particularly important in clinical studies where the relative differences in octadecanoid concentrations between groups are more relevant than the absolute concentration values.

Matrix effects in the surrogate matrix were <16% for all analytes in both platforms (average 8 and 9% for SFC and LC methods, Tables S14 and S15, respectively), with the exception of *cis*-/*trans*-5,6-EpODEs, affected by poor autosampler stability and of some EHODEs in the LC method. Internal standards were not affected in the LC method but suffered from significant signal enhancement in SFC (average 45%). Matrix effects in plasma, evaluated only on internal standards, were found to be significant for all the investigated species, with average 39% signal enhancement in SFC and 35% suppression in LC. A recent study comparing the matrix effects obtained in biological samples with SFC and LC coupled to ESI-MS highlighted the higher occurrence of signal suppression when analyzing plasma by LC–MS.^32^ The nature of the matrix effect measured by SFC-MS, however, depended upon the employed stationary phase. To our knowledge, no evaluation of the matrix effects on the column employed in this study has been reported, but a method for the analysis of plasma eicosanoids in achiral SFC showed similar signal enhancements.^23^

Recoveries were evaluated at three concentration levels and were 30–120% for all analytes in SFC (average values: 74, 80, and 77%, Table S14) and between 13 and 110% in LC (average values: 83, 75, and 71%, Table S15), where the only species with a recovery of <30% was 11(*E*)-10-HOME. Recoveries for PhytoPs were calculated only by LC and were acceptable for all species (83–106%). Differences in recovery calculated with the two methods can be observed for some analytes and can be explained with the independent quantification used, with varying linear ranges and, in turn, different concentration levels spiked in the QC used for the calculations. Recovery in plasma could not be calculated due to the difficulty in obtaining a blank/depleted matrix but was estimated only for the internal standards, which were in line with the results obtained for octadecanoids in the surrogate matrix (54–96%, average 88% for SFC and 53–88%, average 77% for LC). The recovery precision was acceptable for most analytes in both platforms, with a few compounds exceeding the 20% CV threshold at low concentrations. For the LC platform, a trend was observed with the greatest variations for LA- and ALA-derived ketones and compounds for which quantification is affected by variable background levels (*e.g*., 9,10-EpODA, 9,10-EpOME, 12,13-EpOME, and 9,10-DiHOME). In the SFC platform, the greatest deviations were measured for 9(*S*)- and 13(*S*)-HODE, 11(*E*)-10-KOME, and many TriHOME isomers. These species were also affected by poor precision in the matrix at low concentrations (Table S12), indicating that the observed recovery variability is a reflection of the greater method variability for these compounds under these conditions.

Autosampler stability of analytes and internal standards at 8 °C in the reconstitution solvent (MeOH 85%) was evaluated over the course of 96 h at 4 time points: 0, 24, 48, and 96 h. No significant difference was observed with the exception of 5,6-EpODEs, which had a 12–25% degradation after 24 h. The stability of IS was evaluated by area comparison, showing 11–31% signal enhancement at 48–96 h, compatible with day-by-day variability in ESI and observed also for the analytes at the same time points (Table S16 for internal standards, data not shown for analytes).

### Analysis of Octadecanoids in Human Plasma

**Figure 3:**
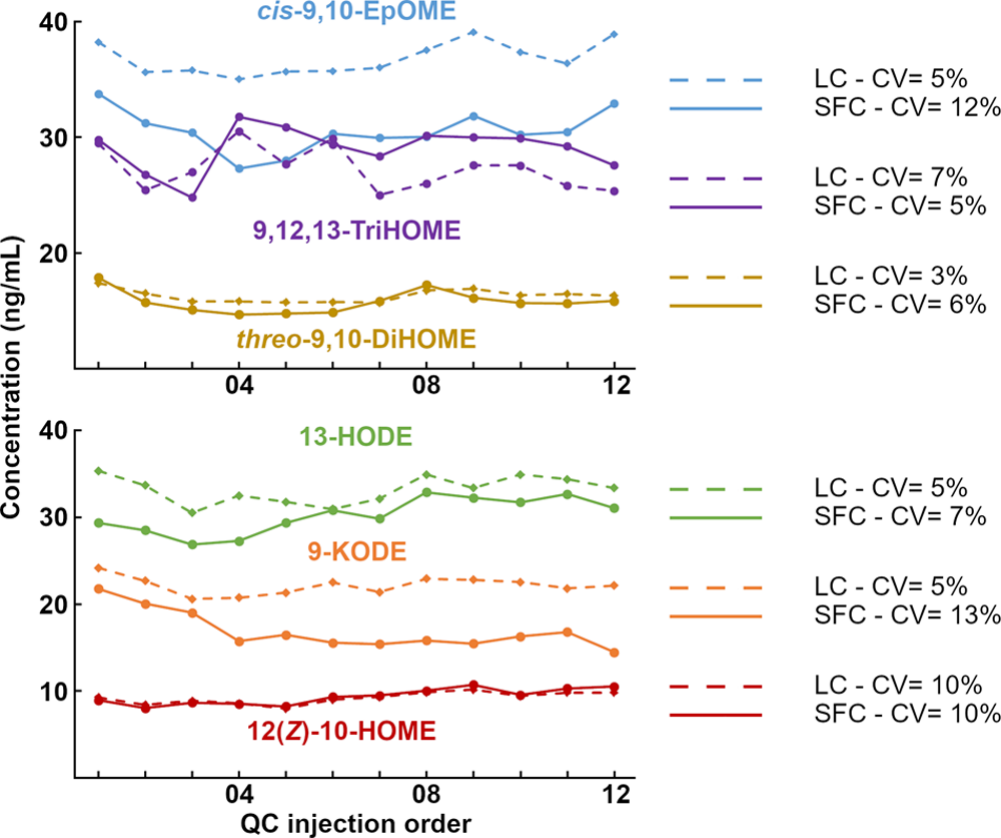
Precision of the SFC- and LC-based methods during study acquisition. Concentration plot for representative analytes for analytical replicates of extraction of QC samples (*n* = 12) acquired over the analysis of 151 mouse plasma samples with the two analytical methods (SFC: injections 1–151; LC: injections 152–303). For the purpose of comparison, multiple isomers resolved by SFC, which co-elute in LC, are reported as the sum of the individual concentrations. See Table S1 for a complete list of isomers. CVs are reported as the percentage relative standard deviation over 12 QC injections. Complete data for all the investigated compounds are reported in Table S17 (SFC) and Table S18 (LC).

To evaluate the stability and precision of the complete analytical procedure over the course of analysis in a typical study, 12 replicates of spiked plasma reference material were injected at regular intervals during a longer sequence consisting of 151 mouse plasma samples. All target octadecanoids were quantified by SFC-MS/MS and LC–MS/MS to evaluate the stability of the obtained concentration values and the precision of the two methods. Subsequently, the results obtained with the two platforms were compared for common analytes to evaluate the accordance between the obtained concentration values. Both methods provided precise quantification for the majority of the investigated analytes (Table S17 for SFC and Table S18 for LC). The average RSD obtained by SFC-MS/MS was 10%, with all compounds having RSD < 15%, with the exception of 13-KOTrE, 5,6-EpODEs (affected by poor autosampler stability), *cis*-12,13-EpODE, and *trans*-9,10-EpOMEs. Precision was slightly better by LC–MS/MS, with the average RSD being 7% and all compounds having RSD < 15%, with the exception of 13-KOTrE and 5,6-EpODEs. Good agreement in the absolute concentration values obtained with the two techniques was achieved for 60% of the analytes (RE < 25%). For other analytes, the measured differences could be caused by the different linear ranges and quantification systems as many of the isomers separated by SFC had to be quantified as the sum for comparison with LC. Additionally, in the SFC-MS/MS method, stereoisomers for which the enantiopure standard is not available are quantified on calibration curves built on a different isomer, resulting in less reliable absolute concentration values. The orthogonality between SFC and LC separations implies potential differences in co-eluting compounds, which can affect the ionization of the same analyte in the two techniques. Similarly, the MS ionization of an analyte is affected by the diverse solvent composition at elution encountered in the two separations. The resolution of chiral species by SFC implies a different elution environment for the resolved stereoisomers, which co-elute in LC. These issues are exacerbated by a lack of internal standards, which are needed to correct for these variations. Discrepancies in the quantification with the two methods, however, were systematic and stable over the injection sequence, as displayed in Figure 3 for six illustrative compounds, indicating good precision and the possibility to quantify relative differences in a study. The detected compounds in human and murine plasma using both methods are shown in Figure S9.

## Conclusions

Current analytical methods targeting oxylipins focus primarily on the arachidonic acid-derived eicosanoids even though 18-carbon fatty acids are the primary dietary fat in humans.^33^ Here, we describe the first analytical platform developed to broadly profile octadecanoids, using 70 custom-synthesized analytical standards to enable unprecedented coverage of enzymatic and autoxidative formation pathways from the main 18-carbon unsaturated fatty acids. The current method is focused primarily on mammalian- and microbial-derived octadecanoids; however, future advances should include plant-derived octadecanoids with a focus on investigating dietary-derived compounds. The chiral SFC-MS/MS method combines the high resolving power of sugar polymer-based chiral selectors with the high flow rates supported by SFC, enabling complex enantioseparations in shorter time frames. The subsequent LC–MS/MS method complements the information obtained by SFC-MS/MS with more robust and sensitive quantification, particularly for autoxidation products.

Both methods were validated and are suitable for the analysis of biological matrices (*e.g*., plasma). The good precision underlines the ability to accurately reproduce relative variations in the concentrations of octadecanoids in biological samples. The accuracy, however, was limited by the low number of available internal standards and, for the SFC-MS/MS method, by the lack of enantiopure analytical standards, which compelled the quantification of 33% of the investigated compounds on the calibration curve of the closest isomer. Additionally, the SFC method experienced increasing baseline pressure with the injection of multiple plasma samples, indicating that, despite SPE modifications, there was still breakthrough of polar impurities. Future efforts should focus on further optimizing the sample preparation to address this issue. In addition, there is a general need to synthesize additional isotopically labeled IS to improve the accuracy as well as expand the methods. The inclusion of additional enantiopure standards would be beneficial to identify single stereoisomers and to establish elution order patterns. Finally, the use of other ionization sources such as APCI could be explored to improve the sensitivity of the detection of low abundance species. This combined analytical platform provides a powerful tool to increase our knowledge of the biological activity of octadecanoids, improving our ability to study their biosynthetic source, to identify bioactive species, to investigate enzymatic alterations, and to elucidate disease mechanisms.

## Supporting Information Available

Schematic of the analytical platform (Section I); complete list of the compounds included in the platform, including nomenclature and origin of all standards (Section II); detailed description of the syntheses of custom-made octadecanoid standards (Section III); complete list of method parameters and detailed description of the characterization of the SFC-MS/MS method (Section IV); complete list of method parameters and detailed description of the characterization of the LC–MS/MS method (Section V); extended discussion of the adaptation of the SPE procedure to the present methodology (Section VI); and complete figures for the validation of the SFC-MS/MS and the LC–MS/MS methods for all investigated analytes and performance of the methods following application in biological matrices (Section VII) (PDF)

## Notes

The National Institute on Aging (NIH) has claimed intellectual property related to application of stable analogs of fatty acid derivatives for treatment of certain inflammatory diseases (PCT/US2018/041086) with this manuscript’s coauthors, C.E.R. and G.S.K., named as inventors.
