# Supplementary material for: Development of a Chiral Supercritical Fluid Chromatography–Tandem Mass Spectrometry and Reversed-Phase Liquid Chromatography–Tandem Mass Spectrometry Platform for the Quantitative Metabolic Profiling of Octadecanoid Oxylipins
Source: Anal Chem. 2022 Oct 11;94(42):14618–26. doi: 10.1021/acs.analchem.2c02601 (PMC9607849; doi:10.1021/acs.analchem.2c02601)
Supplement: Supplementary file 1 — ac2c02601_si_001.pdf [file ac2c02601_si_001.pdf]

Supporting information

Development of a chiral SFC-MS/MS and reversed-phase LC-MS/MS platform for the quantitative metabolic profiling of octadecanoid oxylipins

Alessandro Quaranta[1], Benedikt Zöhrer[2,3], Johanna Revol-Cavalier[1,4], Kurt Benkestock[5], Laurence Balas[6], Camille Oger[6], Gregory S. Keyes[7], Åsa M. Wheelock[2,3], Thierry Durand[6], Jean-Marie Galano[6], Christopher E. Ramsden[7], Mats Hamberg[4,8], Craig E. Wheelock[1,2,9]*

[1]Unit of Integrative Metabolomics, Institute of Environmental Medicine, Karolinska Institutet, 171 77, Stockholm, Sweden

[2]Department of Respiratory Medicine and Allergy, Karolinska University Hospital, 171 76, Stockholm, Sweden

[3]Respiratory Medicine Unit, K2 Department of Medicine Solna and Center for Molecular Medicine, Karolinska Institutet, 171 76, Stockholm, Sweden

[4]Larodan Research Laboratory, Karolinska Institutet, 171 65, Stockholm, Sweden

[5]Waters Sweden AB, 171 65, Stockholm, Sweden

[6]IBMM, Univ Montpellier, CNRS, ENSCM, 34293, Montpellier, France

[7]Laboratory of Clinical Investigation, National Institute on Aging, National Institutes of Health, 21224, Baltimore, MD, USA

[8]Division of Physiological Chemistry II, Department of Medical Biochemistry and Biophysics, Karolinska Institutet, 171 77, Stockholm, Sweden

[9]Gunma University Initiative for Advanced Research (GIAR), Gunma University, Maebashi, Gunma, 371-8511, Japan

*Corresponding author

Craig E. Wheelock

craig.wheelock@ki.se

Table of contents

I) Analytical workflow S4

Figure S1. Scheme of the analytical workflow S4

II) Description of all compounds included in both the SFC and LC-MS/MS methods S5

Table S1. List of compounds included in the SFC and LC-MS/MS methods........ S5

Figure S2. Octadecanoid formation by auto-oxidative processes S12

III) Description and characterization of in-house octadecanoid syntheses S13

Analytical and chromatographical methods S13

10(*R*,*S*)-Hydroxy-12(*Z*)-octadecenoic acid S13

10-Oxo-12(*Z*)-octadecenoic acid........ S13

10-Oxo-11(*E*)-octadecenoic acid S13

10(*R*,*S*)-Hydroxy-11(*E*)-octadecenoic acid S13

10(*R*,*S*)-Hydroxy-12(*Z*),15(*Z*)-octadecadienoic acid........ S14

10-Oxo-12(*Z*),15(*Z*)-octadecadienoic acid........ S14

10-Oxo-11(*E*),15(*Z*)-octadecadienoic acid........ S14

10(*R*,*S*)-Hydroxy-11(*E*),15(*Z*)-octadecadienoic acid S15

Trihydroxyoctadecenoates (TriHOMEs) and trihydroxyoctadecadienoates (TriHODEs) S15

Epoxyoctadecenoates (EpOMEs) and epoxyoctadecadienoates (EpODEs) S15

Dihydroxyoctadecenoates (DiHOMEs) and dihydroxyoctadecadienoates (DiHODEs) S15

Threo-12,13-Dihydroxy-9(*Z*)-octadecenoic acid enriched with the 12(*R*),13(*R*) enantiomer........ S16

Threo-9,10-Dihydroxy-12(*Z*)-octadecenoic acid enriched with the 9(*R*),10(*R*) enantiomer........ S16

9(*R*,*S*)-HOTrE and 13(*R*,*S*)-HOTrE S16

Synthesis scheme A........ S17

Synthesis scheme B........ S18

IV) Development of the chiral SFC method........ S19

Table S2. SFC method gradient S19

Conditioning procedure for Waters AMY-1 chiral column S20

Figure S3. Effects of conditioning on the AMY-1 stationary phase S21

Table S3. Solvent effect on chiral separation of octadecanoids S22

Figure S4. Solvent effect on chiral separation of octadecanoids........ S23

Figure S5. AMY-1 stationary phase resolution upon 9,10- vs. 12,13-oxidized octadecanoids S24

Table S4. Method development parameters for the CEL-2 column........ S25

Figure S6. Comparison of separation of 9,12,13-TriHOMEs on CEL-2 vs. AMY-1 columns........ S26

Table S5. SFC-MS/MS method parameters........ S27

Table S6. SFC-MS/MS quantification parameters........ S31

V) Reversed phase LC method development........ S34

Table S7. LC method gradient S34

Figure S7. Overlaid chromatogram for the LC separation of octadecanoids S35
Table S8. LC-MS/MS method parameter.. S36
Table S9. LC-MS/MS quantification parameters.. S39
VI) Evaluation of the modified SPE procedure.. S41
Experimental: Breakthrough determination of polar species S41
Results and Discussion: Breakthrough determination of polar species.. S41
Table S10. HILIC method for the determination of phosphates in SPE eluates S43
Figure S8. HILIC evaluation of phosphates in eluates with increasing wash volumes.................. S44
Table S11. Polar species removal by additional wash steps.. S45
VII) Validation of the analytical methods S46
Experimental: parameters evaluated during method validation S46
Results and discussion: evaluation of instrumental accuracy for deviating species....................... S47
Table S12. Inter- and intra-day accuracy (%) and precision (% CV) for the SFC-MS/MS method: comparison of solvent and matrix validation S48
Table S13. Inter- and intra-day accuracy (%) and precision (% CV) for the LC-MS/MS method: comparison of solvent and matrix validation S50
Table S14. Recovery and matrix effect in plasma and surrogate matrix (SFC-MS/MS method)... S52
Table S15. Recovery and matrix effect in plasma and surrogate matrix (LC-MS/MS method)..... S55
Table S16. Analyte stability in the autosampler over 96 hours at 8°C... S57
Table S17. Precision of extraction of QC samples analyzed by SFC-MS/MS............................... S59
Table S18. Precision of extraction of QC samples analyzed by LC-MS/MS................................. S61
Figure S9. Representative LC and SFC chromatogram of human and mouse plasma.................... S63
References S65

I) Analytical workflow

The developed analytical workflow includes chiral and achiral characterization of octadecanoids, which are first extracted using solid phase extraction (SPE), then analyzed by chiral supercritical fluid chromatography coupled to tandem mass spectrometry (SFC-MS/MS) and, finally, by reversed phase liquid chromatography coupled to MS/MS (LC-MS/MS). To monitor column and instrument performance, as well as retention time stability, a system suitability test (SST) solution containing all of the target octadecanoids (including all available regioisomers and diastereoisomers) at concentrations ranging 1.0–10.0 ng/mL was prepared, aliquoted in single-use vials, and stored at -80°C. Single vials were thawed prior to analysis of samples and repeatedly injected until a stable signal for all analytes was achieved.

Figure S1. Scheme of the analytical workflow

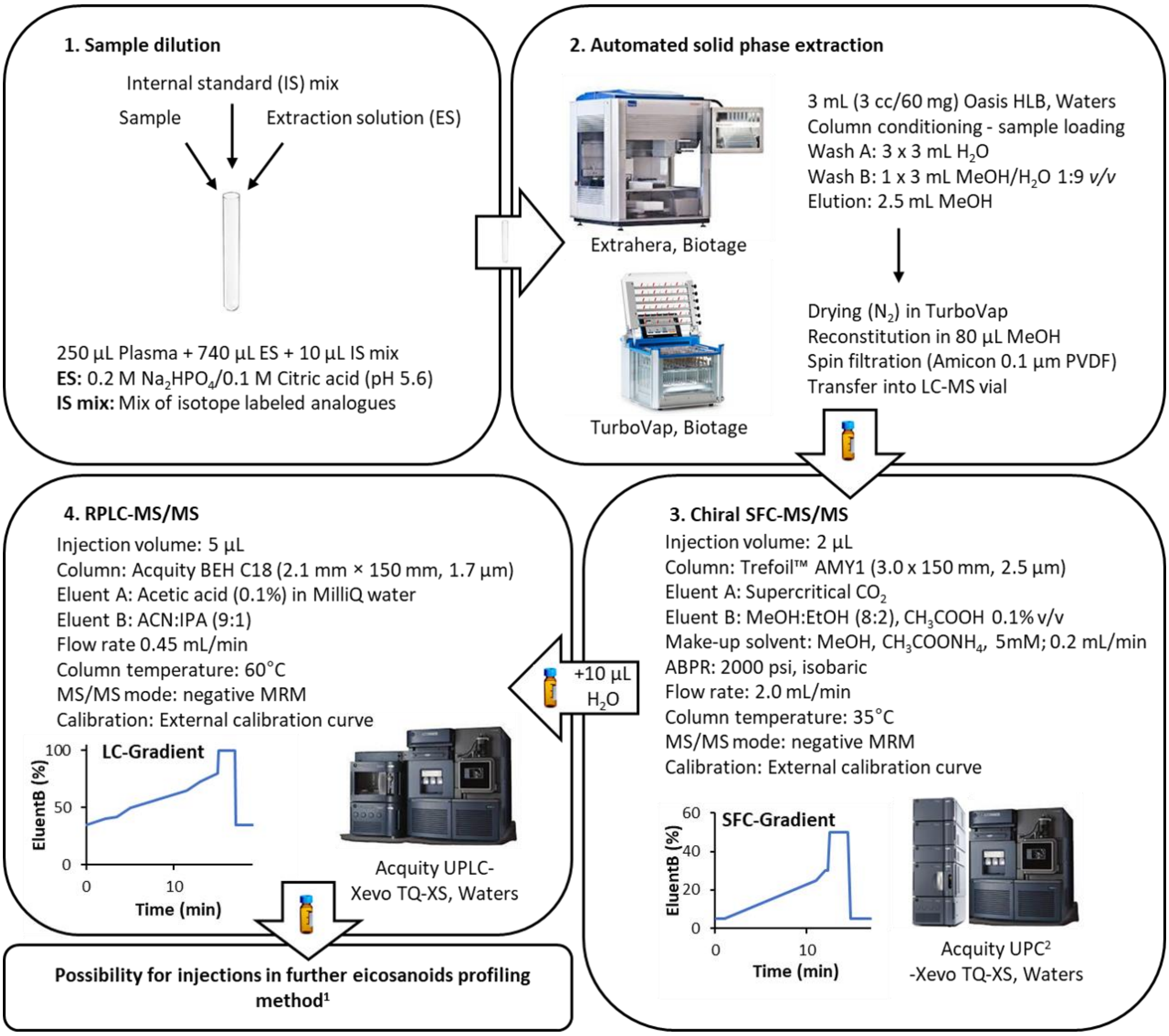

Figure S1. Scheme of the analytical workflow. Workflow overview from sample preparation to SFC- and LC-MS/MS analyses, including the primary parameters employed during the various workup steps. Residual sample volumes enable the further analysis of eicosanoids (*e.g.*, prostaglandins, isoprostanes, and leukotrienes) by employing our previously published method[1].

II) Description of all compounds included in both the SFC and LC-MS/MS methods

Table S1. List of compounds included in the SFC and LC-MS/MS methods

Compound	Parent PUFA[a]	Abbreviation	Structure	Peak number[b] SFC	Peak number[b] LC	Source[c]
***trans*-5,6-epoxy-octadecadienoic acid**	CA	*trans*-5,6-EpODE	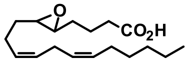	1, 2	68	Larodan
***cis*-5,6-epoxy-octadecadienoic acid**	PNLA	*cis*-5,6-EpODE	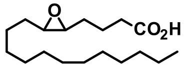	3, 8	66	Larodan
***trans*-9,10-epoxy-octadecenoic acid**	LA	*trans*-9,10-EpOME	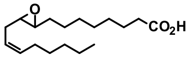	4, 6	75	In-house[2]
***cis*-9,10-epoxy-octadecenoic acid**	LA	*cis*-9,10-EpOME	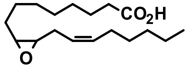	14 (*S*,*R*)<br>28 (*R*,*S*)	74	Larodan
***cis*-9,10-epoxy-octadecenoic acid-d_4**	IS	*cis*-9,10-EpOME-d_4	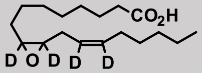	Not Included	72	Cayman
**10-oxo-12(*Z*)-octadecenoic acid**	LA	12(*Z*)-10-KOME	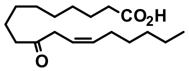	7	67	In-house[f]
***trans*-12,13-epoxy-octadecenoic acid**	LA	*trans*-12,13-EpOME	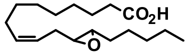	5	73	In-house[2]
***cis*-12,13-epoxy-octadecenoic acid**	LA	*cis*-12,13-EpOME	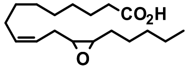	17 (*R*,*S*)<br>25 (*S*,*R*)	70	Larodan
**10-oxo-12(*Z*),15(*Z*)-octadecadienoic acid**	ALA	12(*Z*),15(*Z*)-10-KODE	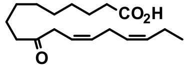	9	55	In-house[f]
***cis*-15,16-epoxy-octadecadienoic acid**	ALA	*cis*-15,16-EpODE	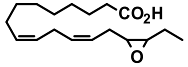	10	58	In-house[2]
**10-oxo-11(*E*),15(*Z*)-octadecadienoic acid**	ALA	11(*E*),15(*Z*)-10-KODE	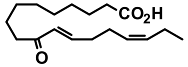	11	55	In-house
***trans*-9,10-epoxy-octadecanoic acid**	OA	*trans*-9,10-EpODA	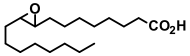	12, 15	77	Larodan

Compound	Parent PUFA[a]	Abbreviation	Structure	Peak number[b] SFC	Peak number[b] LC	Source[c]
***cis*-9,10-epoxy-octadecanoic acid**	OA	*cis*-9,10-EpODA	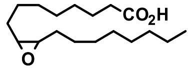	21 (*S*,*R*)*<br>29 (*R*,*S*)*	76	Larodan
***cis*-9,10-epoxy-octadecadienoic acid**	ALA	*cis*-9,10-EpODE	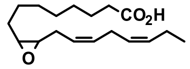	13 (*S*,*R*)*<br>19 (*R*,*S*)*	60	In-house[2]
**9-oxo-octadecadienoic acid**	LA	9-KODE	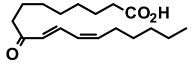	16	63	Larodan
**13-oxo-octadecatrienoic acid**	ALA	13-KOTrE	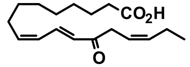	22	50	Larodan
**13-oxo-octadecadienoic acid**	LA	13-KODE	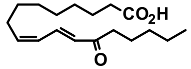	23	61	Larodan
**10-oxo-11(*E*)-octadecenoic acid**	LA	11(*E*)-10-KOME	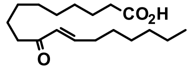	24	71	In-house
**13-oxo-octadecadienoic acid-d$_3$**	IS	13-KODE-d$_3$	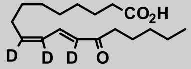	18	59	Cayman
**9-oxo-octadecatrienoic acid**	ALA	9-KOTrE	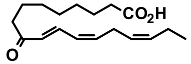	26	51	Larodan
***cis*-12,13-epoxy-octadecadienoic acid**	ALA	*cis*-12,13-EpODE	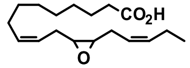	27	62	Larodan
***cis*-12,13-epoxy-octadecenoic acid-d$_4$**	IS	*cis*-12,13-EpOME-d$_4$	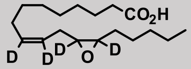	20	69	Cayman
**13-hydroxy-octadecatrienoic acid**	ALA	13-HOTrE	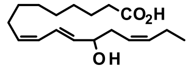	30 (*R*)<br>42 (*S*)	47	In-house<br>Larodan
**13-hydroxy-octadecadienoic acid**	LA	13-HODE	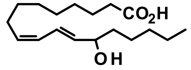	31 (*R*)<br>64 (*S*)	54	Larodan<br>Larodan
**8(*R*)-11(*S*)-dihydroxy-octadecadienoic acid**	LA	8(*R*),11(*S*)-DiHODE	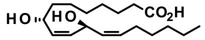	Not Included	23	Larodan

Compound	Parent PUFA[a]	Abbreviation	Structure	Peak number[b] SFC	Peak number[b] LC	Source[c]
**9-hydroxy-octadecadienoic acid**	LA	9-HODE	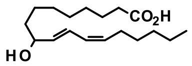	32 (*R*)<br>50 (*S*)	47	Larodan<br>Larodan
**10-hydroxy-12(*Z*)-octadecenoic acid**	LA	12(*Z*)-10-HOME	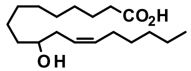	33 (*R*)*<br>36 (*S*)*	64	In-house[f]
**12-oxo-13-hydroxy-octadecenoic acid**	LA	13-OH-12-KOME	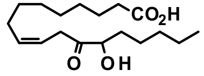	34	43	Larodan
**10-hydroxy-11(*E*),15(*Z*)-octadecadienoic acid**	ALA	11(*E*),15(*Z*)-10-HODE	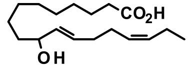	35 (*R*)*<br>39 (*S*)*	52	In-house
**10-hydroxy-12(*Z*),15(*Z*)-octadecadienoic acid**	ALA	12(*Z*),15(*Z*)-10-HODE	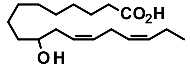	37 (*R*)*<br>57 (*S*)*	52	In-house[f]
**11-hydroxy-*trans*-12,13-epoxy-octadecenoic acid**	LA	11-OH-*trans*-12,13-EpOME	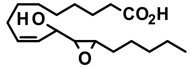	38, 41, 51, 54	37,40	In-house[3]
**11-hydroxy-*trans*-9,10-epoxy-octadecenoic acid**	LA	11-OH-*trans*-9,10-EpOME	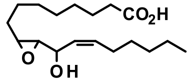	40, 43, 45, 52	36,41	In-house[3]
**10-hydroxy-11(*E*)-octadecenoic acid**	LA	11(*E*)-10-HOME	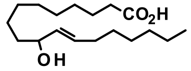	44 (*R*)*<br>47 (*S*)*	65	In-house
**9-hydroxy-octadecatrienoic acid**	ALA	9-HOTrE	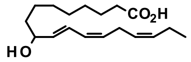	46 (*R*)<br>70 (*S*)	46	In-house<br>Larodan
**13(*S*)-hydroxy-octadecatrienoic acid-γ**	GLA	13(*S*)-HOTrE-γ	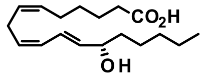	48	48	Larodan
**9(*S*)-hydroxy-octadecadienoic acid-d$_4$**	IS	9(*S*)-HODE-d$_4$	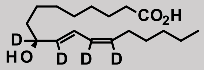	49	56	Cayman
***threo*-9,10-dihydroxy-octadecenoic acid-d$_4$**	IS	*threo*-9,10-DiHOME-d$_4$	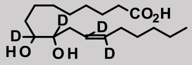	53, 73	38	Cayman

Compound	Parent PUFA[a]	Abbreviation	Structure	Peak number[b] SFC	Peak number[b] LC	Source[c]
**9-oxo-*trans*-12,13-epoxy-octadecenoic acid**	LA	9-oxo-*trans*-12,13-EpOME (EKODE)	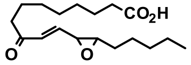	56, 67	45	Larodan
**9,10-dihydroxy-octadecenoic acid**	LA	9,10-DiHOME	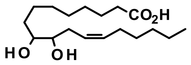	63, 71 (*erythro*)<br>58 (*R*,*R*), 67 (*S*,*S*) (*threo*)	39 (*erythro*)<br>33 (*threo*)	In-house[2]<br>Larodan
**12,13-dihydroxy-octadecenoic acid**	LA	12,13-DiHOME	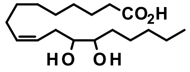	60 (*threo*)<br>60, 68 (*erythro*)	35 (*threo*)<br>30 (*erythro*)	Larodan<br>In-house[2]
**12,13-dihydroxy-octadecadienoic acid**	ALA	12,13-DiHODE	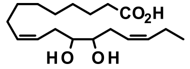	61	28 (*threo*)<br>25 (*erythro*)	Larodan<br>In-house[2]
***threo*-12,13-dihydroxy-octadecenoic acid-d_4**	IS	*threo*-12,13-DiHOME-d_4	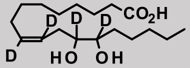	55, 59	34	Cayman
**13-hydroxy-*trans*-9,10-epoxy-octadecenoic acid**	LA	13-OH-*trans*-9,10-EpOME	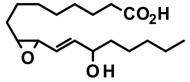	65, 89, 92	32	In-house[3]
**9,10-dihydroxy-octadecadienoic acid**	ALA	9,10-DiHODE	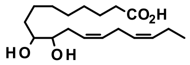	66 (*R*,*R*)*, 86 (*S*,*S*)* (*threo*)<br>79 (*erythro*)	29 (*threo*)<br>26 (*erythro*)	In-house[2]<br>In-house[2]
**13-hydroxy-*trans*-9,10-epoxy-octadecenoic acid-d_5**	IS	13-OH-*trans*-9,10-EpOME-d_5	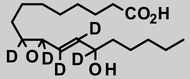	62	31	In-house[3]
**9-hydroxy-*trans*-12,13-epoxy-octadecenoic acid**	LA	9-OH-*trans*-12,13-EpOME	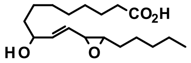	72, 97, 100	32	In-house[3]
**13(*S*)-hydroxy-octadecadienoic acid-d_4**	IS	13(*S*)-HODE-d_4	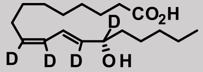	69	53	Cayman
**9,10-dihydroxy-octadecanoic acid**	OA	9,10-DiHODA	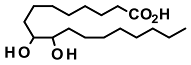	77 (*R*,*R*)*, 84 (*S*,*S*)* (*threo*)<br>84, 90 (*erythro*)	49 (*threo*)<br>44 (*erythro*)	Larodan

Compound	Parent PUFA[a]	Abbreviation	Structure	Peak number[b] SFC	Peak number[b] LC	Source[c]
**15,16-dihydroxy-octadecadienoic acid**	ALA	15,16-DiHODE	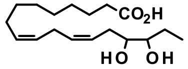	75 (*threo*)<br>75, 78 (*erythro*)	27 (*threo*)<br>24 (*erythro*)	In-house[2]
**9,10,13-trihydroxy-octadecenoic acid**	LA	9,10,13-TriHOME	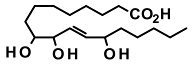	74 (*S*,*S*,*R*)	20[#]	In-house[4–6]
				83 (*R*,*S*,*R*)		In-house[4–6]
				88 (*S*,*S*,*S*)		Larodan
				91 (*R*,*S*,*S*)		In-house[4–6]
				94 (*S*,*R*,*R*)		In-house[4–6]
				98 (*S*,*R*,*S*)		In-house[4–6]
				101 (*R*,*R*,*R*)		In-house[4–6]
				103 (*R*,*R*,*S*)		In-house[4–6]
**11(*R*),12(*S*),13(*S*)-trihydroxy-octadecenoic acid**	LA	11(*R*),12(*S*),13(*S*)-TriHOME	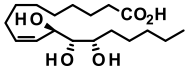	80	21	Larodan
**9(*S*),10(*S*),11(*R*)-trihydroxy-octadecenoic acid**	LA	9(*S*),10(*S*),11(*R*)-TriHOME	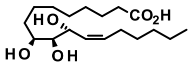	81	22	Larodan
**9,12,13-trihydroxy-octadecenoic acid**	LA	9,12,13-TriHOME	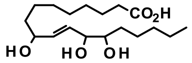	87 (*R*,*S*,*S*)	18[#]	In-house[4–6]
				93 (*S*,*S*,*R*)		In-house[4–6]
				95 (*S*,*R*,*S* + *R*,*S*,*R*)		In-house[4–6]
				99 (*S*,*S*,*S*)		Larodan
				102 (*S*,*R*,*R*)		In-house[4–6]
				104 (*R*,*R*,*S*+*R*,*R*,*R*)		In-house[4–6]
**9(*S*),10(*S*),13(*S*)-trihydroxy-octadecadienoic acid**	ALA	9(*S*),10(*S*),13(*S*)-TriHODE	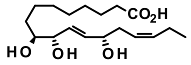	82	12	Larodan

Compound	Parent PUFA[a]	Abbreviation	Structure	Peak number[b] SFC	Peak number[b] LC	Source[c]
**9(*S*),10(*S*),11(*R*)-trihydroxy-octadecadienoic acid**	ALA	9(*S*),10(*S*),11(*R*)-TriHODE	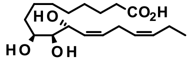	85	17	Larodan
**9(*S*),12(*S*),13(*S*)-$^{13}C_3$-trihydroxy-octadecenoic acid**	IS	9(*S*),12(*S*),13(*S*)-$^{13}C_3$-TriHOME	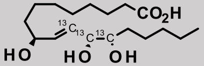	96	19	In-house[4]
**9(*S*),12(*S*),13(*S*)-trihydroxy-octadecadienoic acid**	ALA	9(*S*),12(*S*),13(*S*)-TriHODE	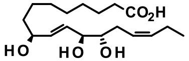	105	11	Larodan
**(*R*,*E*)-8-(2-(3-hydroxypent-1-en-1-yl)-5-oxocyclopent-1-en-1-yl)octanoic acid**	ALA	16-B_1-PhytoP$_{ALA}$	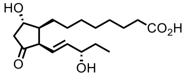	Not Included	16	In-house[7]
**(*S*,*E*)-11-(2-ethyl-3-oxocyclopent-1-en-1-yl)-9-hydroxyundec-10-enoic acid**	ALA	9-L_1-PhytoP$_{ALA}$	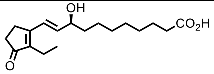	Not Included	15	In-house[7]
**7-((1*S*,2*R*,3*R*,5*S*)-3,5-dihydroxy-2-((*S*,*E*)-3-hydroxyoct-1-en-1-yl)cyclopentyl)heptanoic acid[e]**	DGLA[e]	15-F_{1t}-PhytoP$_{DGLA}$	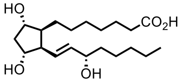	Not Included	14	In-house[d]
**(*E*)-10-((1*S*,2*R*,3*R*,5*S*)-3,5-dihydroxy-2-pentylcyclopentyl)-8-hydroxydec-9-enoic acid[e]**	DGLA[e]	8-F_{1t}-PhytoP$_{DGLA}$	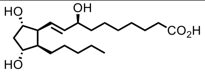	Not Included	13	In-house[d]
**8-((1*S*,2*R*,3*R*,5*S*)-3,5-dihydroxy-2-((*E*)-3-hydroxyhex-1-en-1-yl)cyclopentyl)octanoic acid**	IS	16-F_{1t}-PhytoP-C19	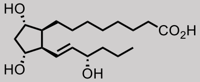	Not Included	10	In-house[d]
**(*R*,*E*)-11-((1*R*,2*S*,5*R*)-2-ethyl-5-hydroxy-3-oxocyclopentyl)-9-hydroxyundec-10-enoic acid**	ALA	*ent*-9-D_{1t}-PhytoP$_{ALA}$	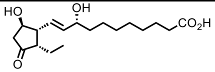	Not Included	9	In-house[d]
**5-((1*S*,2*R*,3*R*,5*S*)-3,5-dihydroxy-2-((*E*)-3-hydroxyoct-1-en-1-yl)cyclopentyl)pentanoic acid**	GLA	13-F_{1t}-PhytoP$_{GLA}$	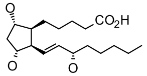	Not Included	8	In-house[d]
**(*E*)-8-((1*S*,2*R*,3*R*,5*S*)-3,5-dihydroxy-2-pentylcyclopentyl)-6-hydroxyoct-7-enoic acid**	GLA	6-F_{1t}-PhytoP$_{GLA}$	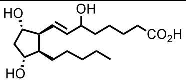	Not Included	7	In-house[d]
**(*S*,*E*)-11-((1*S*,2*R*,3*R*,5*S*)-2-ethyl-3,5-dihydroxy-cyclopentyl)-9-hydroxyundec-10-enoic acid**	ALA	9-F_{1t}-PhytoP$_{ALA}$	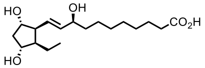	Not Included	6	In-house[8]
**8-((1*S*,2*R*,3*R*,5*S*)-3,5-dihydroxy-2-((*S*,*E*)-3-hydroxypent-1-en-1-yl)cyclopentyl)octanoic acid**	ALA	16-F_{1t}-PhytoP$_{ALA}$	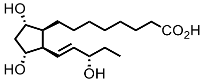	Not Included	5	In-house[8]
**(*S*,*E*)-8-((1*S*,2*R*,3*R*,5*S*)-3,5-dihydroxy-2-((*Z*)-pent-2-en-1-yl)cyclopentyl)-6-hydroxyoct-7-enoic acid**	SDA	6-F_{2t}-PhytoP$_{SDA}$	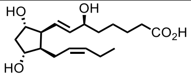	Not Included	4	In-house[d]

Compound	Parent PUFA[a]	Abbreviation	Structure	Peak number[b] SFC	Peak number[b] LC	Source[c]
**(*Z*)-8-((1*S*,2*R*,3*R*,5*S*)-3,5-dihydroxy-2-((*S*,*E*)-3-hydroxypent-1-en-1-yl)cyclopentyl)oct-6-enoic acid**	SDA	16-F_{2t}-$PhytoP_{SDA}$	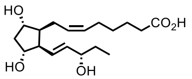	Not Included	3	In-house[d]
**8-((2*R*,3*S*,5*R*)-5-((1*S*,*E*)-1,4-dihydroxyhex-2-en-1-yl)-3-hydroxytetrahydrofuran-2-yl)octanoic acid**	ALA	*ent*-16-13-*epi*-ST-Δ^{14}-9-PhytoF	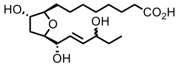	Not Included	2	In-house[9]
**(12*S*,*E*)-12-((2*R*,4*S*,5*R*)-5-ethyl-4-hydroxytetrahydrofuran-2-yl)-9,12-dihydroxydodec-10-enoic acid**	ALA	*ent*-9-12-*epi*-ST-Δ^{10}-13-PhytoF	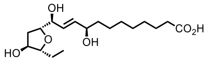	Not Included	1	In-house[9]

[a] Parent polyunsaturated fatty acid (PUFA) abbreviations: OA, Oleic Acid; LA, Linoleic Acid; ALA, α-Linolenic Acid; GLA, γ-Linolenic Acid; PNLA, Pinolenic Acid; CA, Columbinic Acid; DGLA, Dihomo-γ-Linolenic Acid; SDA, Stearidonic Acid; IS, Internal Standard (internal standards are highlighted in gray).

[b] Peak numbers refer to the elution order in the two analytical methods.

[c] Compounds were purchased from either Cayman Chemical (Ann Arbor, MI, USA) or Larodan AB (Solna, Sweden). Non-commercial compounds are described as "in-house" and were custom synthesized as described in **Section III Description and characterization of in-house octadecanoid syntheses.** Alternatively, a citation is provided for those compounds previously synthesized.

[d] These compounds were in-house synthesized and the details of their synthesis have been submitted for publication.

[e] These compounds are formed from the 20-carbon PUFA DGLA and are subsequently not octadecanoids.

[f] Following discussions generated by the first version of this manuscript during early stages of the publication process, Cayman Chemicals started the production of these compounds and now include them in its catalogue.

* Order of elution not confirmed with enantiopure standard due to unavailability but inferred by comparison with similar compounds under the same conditions.

[#] Eluted as three non-resolved peaks, integrated as a single peak.

Figure S2. Octadecanoid formation by auto-oxidative processes

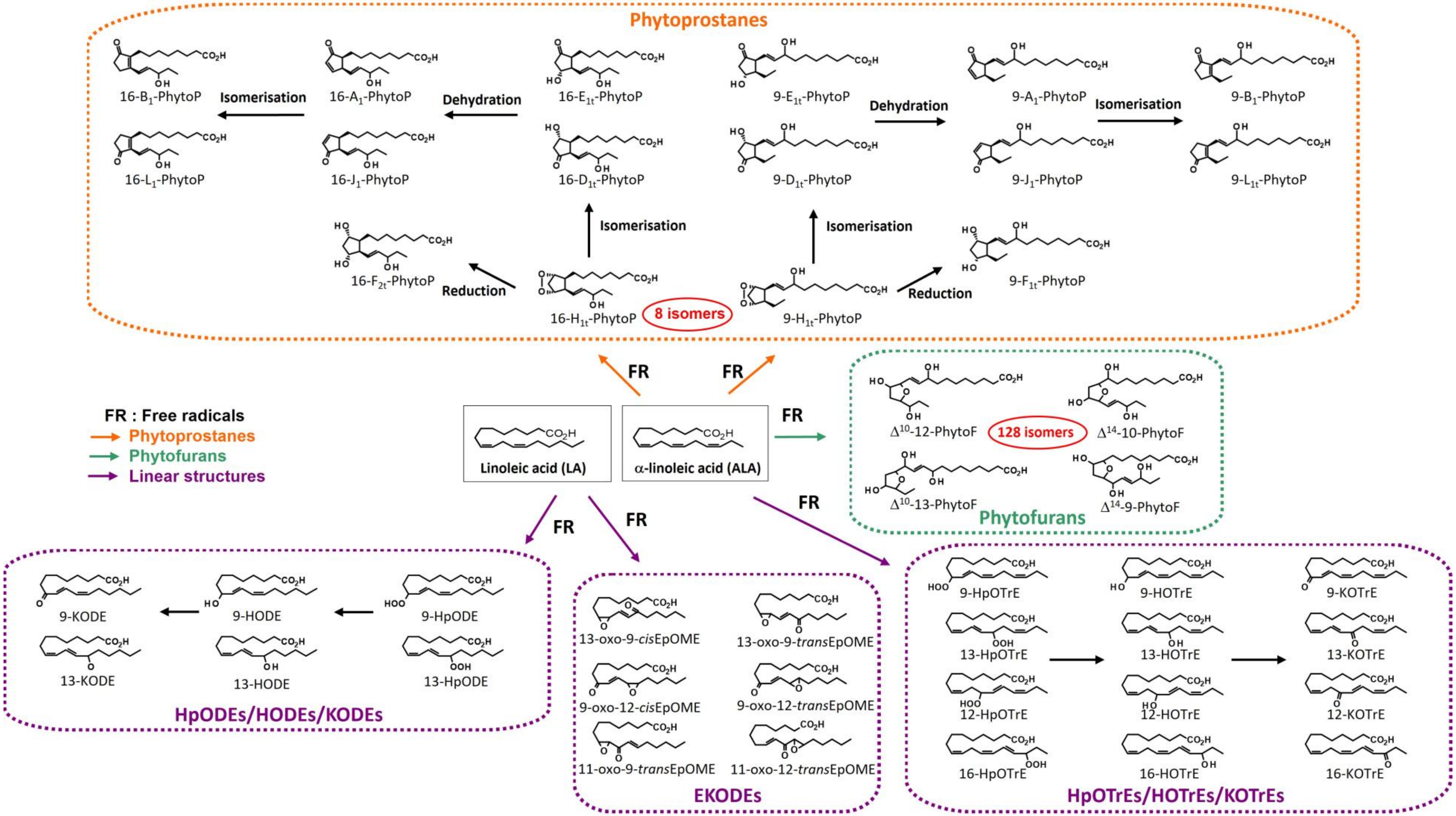

Figure S2. **Octadecanoid formation by auto-oxidative processes.** Major pathways of ROS- and lipid autoxidation-derived octadecanoid formation from linoleic acid (LA) and α-linolenic acid (ALA). See Table S1 for a list of complete octadecanoid nomenclature.

III) Description and characterization of in-house octadecanoid syntheses

Analytical and chromatographical methods. Gas chromatography-mass spectrometry (GC-MS) was performed using an Agilent mass selective detector model 5977E connected to an Agilent model 7820A gas chromatograph. A capillary column of 5% phenylmethylsiloxane (12 m, 0.33 µm film thickness) with helium as the carrier gas was used. The temperature was raised from 80°C to 320°C at a rate of 10°C/min. Reversed-phase HPLC (RP-HPLC) was performed with a column of 250 x 10 mm Nucleosil 100-7 C18 whereas a column of 250 x 10 mm Nucleosil 50-7 was used for straight-phase HPLC (SP-HPLC). In both cases the flow rate was 4 mL/min.

10(R,S)-Hydroxy-12(Z)-octadecenoic acid. A solution of 1-heptyne (31.3 mmol) in 100 mL of dry THF at -78°C was treated with butyllithium (30.8 mmol). After stirring for 1 h, a solution of methyl 10,11-epoxyundecanoate (16.4 mmol) in 50 mL of THF was added dropwise followed by BF_3-Et_2O (15.2 mmol)[10]. The mixture was stirred at -78°C for 1.5 h and subsequently quenched by the addition of saturated ammonium chloride. Extraction with diethyl ether followed by silica gel column chromatography (diethyl ether-hexane (1:3, v/v)) provided methyl 10-hydroxy-12-octadecynoate (12.2 mmol (yield, 74%)). Part of this material (3.2 mmol) was dissolved in 20 mL of toluene and stirred with Lindlar catalyst (100 mg) under hydrogen gas for 1.5 h. The product was saponified by treatment with 0.4 M NaOH in methanol-water 4:1 at 22°C for 15 h and purified by silica gel column chromatography (diethyl ether-hexane (2:3, v/v)). Crystallization from diethyl ether-hexane at -20°C afforded the title compound (2.0 mmol (yield, 62%), m.p., 47-48°C). The mass spectrum of the methyl ester showed prominent ions at *m/z* 312 (M^+), 294 (M^+ - H_2O), 201 (HO^+=CH-$(CH_2)_8$-$COOCH_3$), and 169 (201 - CH_3OH).

10-Oxo-12(Z)-octadecenoic acid. 0.67 mmol of the above 10-hydroxy-12(*Z*)-octadecenoic acid was dissolved in 8 mL of dichloromethane and stirred at 0°C with Dess-Martin periodinane (400 mg) for 1 h. The product was subjected to silica gel column chromatography (diethyl ether-hexane (1:4, v/v)) and further purified by RP-HPLC (mobile phase, acetonitrile-water-acetic acid (70:30:0.01, v/v/v)). Crystallization from diethyl ether-hexane at -20°C afforded the pure title compound (0.51 mmol (yield, 76%), m.p. 57.5-58.0°C). The mass spectrum of the methyl ester showed prominent ions at *m/z* 310 (M^+), 279 (M^+ - OCH_3), 199 (O^+≡C-$(CH_2)_8$-$COOCH_3$), and 139 (O^+≡C-CH_2-CH=CH-C_5H_{11}).

10-Oxo-11(E)-octadecenoic acid. The above 10-oxo-12(*Z*)-octadecenoic acid (0.41 mmol) was dissolved in 20 mL of THF and treated with 1 mL 60% perchloric acid at 50°C for 1.5 h. The product was subjected to RP-HPLC (mobile phase, acetonitrile-water-acetic acid (65:35:0.01, v/v/v)) and crystallized from diethyl ether-hexane at -20°C affording the title compound as a white solid (0.26 mmol, 63% yield), m.p. 53.0-53.5°C. The UV spectrum showed an absorption band with λ_{max} (EtOH) = 223 nm due to the α,β-unsaturated ketone, and the mass spectrum of the methyl ester showed prominent ions at *m/z* 310 (M^+), 279 (M^+ - OCH_3), 225 (M^+ - C_6H_{13}), 199 (O^+≡C-$(CH_2)_8$-$COOCH_3$), and 139 (O^+≡C-CH=CH-C_6H_{13})

10(R,S)-Hydroxy-11(E)-octadecenoic acid. 10-oxo-11(*E*)-octadecenoic acid (0.18 mmol) was dissolved in 3 mL of methanol and treated at 0°C with 50 mg of $NaBH_4$. After 10 min at 0°C

and 10 min at 22°C the solution was carefully acidified and extracted with ethyl acetate affording a white solid which was purified by RP-HPLC (mobile phase, acetonitrile-water-acetic acid (60:40:0.01, v/v/v)). Crystallization from diethyl ether-hexane at -20°C afforded the pure title compound as a white solid (0.11 mmol, 61% yield). The mass spectrum of the methyl ester-Me_3Si ether derivative showed prominent ions at *m/z* 312 (M^+), 294 (M^+ - H_2O), 227 (M^+ - C_6H_{13}), 195 (227 - CH_3OH), and 141 (HO^+=CH-CH=CH-C_6H_{13}).

10(R,S)-Hydroxy-12(Z),15(Z)-octadecadienoic acid. The title compound was prepared by two successive acetylene couplings as shown in Scheme A. Trimethylsilylacetylene (28.6 mmol) in 85 mL of THF was treated under argon at -78°C with n-butyllithium (30 mmol). After stirring at -78°C for 1 h a solution of methyl 10,11-epoxyundecanoate (23.8 mmol) in 16 mL of THF was added followed by BF_3-Et_2O (34.8 mmol). After stirring at -78°C for 1.5 h, saturated ammonium chloride was added and the product extracted with diethyl ether. The product (7.65 g of a pale-yellow oil) was dissolved in 300 mL of methanol-THF (1:1, v/v) and stirred with K_2CO_3 (17 mmol) at 22°C for 15 h. Extraction with diethyl ether followed by silica gel column chromatography (diethyl ether - hexane (3:7, v/v)) afforded the deprotected alkyne as a pale-yellow viscous oil (4.8 g, 19.8 mmol, yield from epoxyundecanoate, 83%). Copper-catalyzed coupling of this material to 1-bromo-2-pentyne was effected using the conditions described by Pivnitsky et al.[11] affording, after silica gel column chromatography, 5.8 g of the crude desired product. Part of this material (3 g) was crystallized from 150 ml of hexane at +4°C affording pure methyl 10-hydroxy-12,15-octadecadiynoate (1.5 g, 4.9 mmol; estimated total yield, 9.5 mmol, 48%). Partial hydrogenation was effected by stirring the diyne (4.9 mmol) with 330 mg of Lindlar catalyst and 0.11 mL of quinoline in 50 mL of methanol under H_2 for 3.5 h. The product was directly saponified by treatment with 0.33 M NaOH in methanol-water (5:1, v/v) at 22°C for 15 h affording material (1.1 g of oil) which was purified by silica gel column chromatography (diethyl ether-hexane (2:3, v/v)). Crystallization from diethyl ether-hexane at -20°C, afforded 2.9 mmol (yield, 59%) of >95% pure material. Final purification of an aliquot of this material by RP-HPLC (mobile phase, acetonitrile-water-acetic acid (55:45:0.01, v/v/v)) yielded the pure title compound as a colorless viscous oil which solidified upon standing at room temperature. The mass spectrum of the methyl ester-Me_3Si ether derivative showed prominent ions at *m/z* 292 (M^+ - H_2O), 279 (M^+ - OCH_3), 201 (HO^+=CH-$(CH_2)_8$-$COOCH_3$), and 169 (201 - CH_3OH).

10-Oxo-12(Z),15(Z)-octadecadienoic acid. An aliquot of the above hydroxy acid (0.53 mmol) was treated with Dess-Martin periodinane and purified by silica gel column chromatography and crystallization as described above for the corresponding hydroxy-monoenoic acid, producing the title compound (0.27 mmol, yield 51%) as a sticky white solid. The mass spectrum of the methyl ester showed prominent ions at *m/z* 308 (M^+), 277 (M^+ - OCH_3), 199 (O^+≡C-$(CH_2)_8$-$COOCH_3$), 167 (199 - CH_3OH), and 139.

10-Oxo-11(E),15(Z)-octadecadienoic acid. Perchloric acid-catalyzed isomerization of the 10-oxo-12,15-octadecadienoic acid into the α,β-unsaturated ketone and its further purification was carried out as described for the corresponding keto-monoenoic acid, producing the title compound as a white solid showing λ_{max} (EtOH) = 223 nm. The mass spectrum of the methyl ester showed prominent ions at *m/z* 308 (M^+), 277 (M^+ - OCH_3), 240 (M^+ - CH_2-CH=CH-

C_2H_5 + H), 199 (O^+≡C-$(CH_2)_8$-$COOCH_3$), 180, and 137 (O^+≡C-CH=CH-$(CH_2)_2$-CH=CH-C_2H_5).

10(R,S)-Hydroxy-11(E),15(Z)-octadecadienoic acid. The 10-oxo-11,15-octadecadienoic acid was subjected to $NaBH_4$ reduction as described for the corresponding hydroxy-monoenoic acid. After purification by RP-HPLC (mobile phase, acetonitrile-water-acetic acid (55:45:0.01, v/v/v)) the title compound was obtained as a white semisolid. The mass spectrum of the methyl ester showed prominent ions at *m/z* 292 (M^+ - H_2O), 279 (M^+ - OCH_3), 201 (HO^+=CH-$(CH_2)_8$-$COOCH_3$), and 169 (201 - CH_3OH). The mass spectra of the methyl esters of the 10-hydroxy-12(*Z*),15(*Z*)- and 10-hydroxy-11(*E*),15(*Z*)-isomers showed similar fragmentation patterns, however, the spectra of the methyl ester/Me_3Si derivatives were distinct having *m/z* 273 (Me_3SiO^+=CH-$(CH_2)_8$-$COOCH_3$) and *m/z* 211 (Me_3SiO^+=CH-CH=CH-$(CH_2)_2$-CH=CH-C_2H_5), respectively, as their most predominant ions.

Trihydroxyoctadecenoates (TriHOMEs) and trihydroxyoctadecadienoates (TriHODEs). The methodology used for preparation of most of the stereochemically defined TriHOMEs and TriHODEs is depicted in Scheme B[4,5,12]. Thus, treatment of conjugated fatty acid hydroperoxides obtained by lipoxygenase-catalyzed oxygenation of linoleic or α-linolenic acids (9(*S*)- and 13(*S*)-HpODEs, 9(*S*)- and 13(*S*)-HpOTrEs) with vanadium oxyacetylacetonate yielded diastereomeric α,β-epoxy alcohols which were separated by SP-HPLC. Treatment of the individual isomers with dilute hydrochloric acid produced diastereomeric trihydroxy acids containing the 1,2,5-trihydroxy-3(*E*)-pentene structural element. These were separated by TLC or SP-HPLC. In addition, smaller amounts of diastereomeric trihydroxy acids of the 1,2,3-trihydroxy-4(*Z*)-pentene type appeared[12]. Synthesis of the latter type of compounds having defined stereochemistry was carried out by treatment of epoxy alcohols with 0.29 M NaOH in THF-water (20:120, v/v) at 60°C for 1 h, a reaction taking place with inversion of the configuration at C-11 (Scheme B).

By using 9(*R,S*)- and 13(*R,S*)-HpODEs[6] as starting materials instead of lipoxygenase-derived hydroperoxides it was possible to generate TriHOMEs in racemic form using the same methodology. Another way of preparing racemic TriHOMEs was by autoxidation of linoleic acid followed by separation of products by TLC and SP-HPLC[4,5].

Epoxyoctadecenoates (EpOMEs) and epoxyoctadecadienoates (EpODEs). Racemic EpOMEs and EpODEs were prepared by monoepoxidation of linoleic and α-linolenic acids, respectively[2]. Of the optically pure compounds, 12(*S*),13(*R*)- and 12(*R*),13(*S*)-epoxy-9(*Z*)-octadecenoic acids ((+)- and (-)-vernolic acids, respectively) were purchased from Larodan Co., Stockholm, Sweden, whereas 9(*R*),10(*S*)-epoxy-12(*Z*)-octadecenoic acid ((+)-coronaric acid) was isolated from seeds of *Chrysanthemum coronarium* as described[13]. 9(*R*),10(*S*)-Epoxyoctadecanoic acid was prepared by catalytic hydrogenation of (+)-coronaric acid (15 mg in 3 mL of methanol, platinum catalyst) and was obtained in crystalline form (m.p. 56.5-57.5°C) following crystallization from hexane.

Dihydroxyoctadecenoates (DiHOMEs) and dihydroxyoctadecadienoates (DiHODEs). Vicinal diol derivatives of linoleic and α-linolenic acids were prepared by perchloric acid-catalyzed hydrolysis of the above EpOMEs and EpODEs or purchased from Larodan Co.

Threo-12,13-Dihydroxy-9(Z)-octadecenoic acid enriched with the 12(R),13(R) enantiomer. Earlier work in our laboratory has shown that acetolysis of (+)-vernolic acid (12(*S*),13(*R*)-epoxy-9(*Z*)-octadecenoic acid) takes place with a slight preference for the homoallylic position, thus producing an excess of the 12(*R*)-acetoxy-13(*R*)-hydroxy derivative relative to the 12(*S*)-hydroxy-13(*S*)-acetoxy compound. In addition, a slight separation of these two regioisomers on TLC allowed further enrichment of the first-mentioned compound. Accordingly, (+)-vernolic acid methyl ester (25 mg) and glacial acetic acid (1 mL) were kept at 70°C for 4 h. The product was subjected to preparative TLC using a solvent system of ethyl acetate-hexane (25:75, v/v). The upper half of the band visualized by spraying with 2´,7´-dichlorofluorescein was recovered and the product consisting of methyl 12-acetoxy-13-hydroxy- and 12-hydroxy-13-acetoxyoctadecenoates in proportion 3:1 according to analysis by GC-MS was saponified by treatment with 0.2 M NaOH in methanol-water 7:3 at room temperature for 18 h. The product was crystallized from diethyl ether-hexane at -20°C to provide a sample ready for analysis by chiral-phase HPLC.

Threo-9,10-Dihydroxy-12(Z)-octadecenoic acid enriched with the 9(R),10(R) enantiomer. The title compound was prepared from (+)-coronaric acid methyl ester (methyl 9(*R*),10(*S*)-epoxy-12(*Z*)-octadecenoate, 56 mg) using the protocol given above. However, in this case the lower part of the TLC band, in which the 9-hydroxy-10-acetoxy isomer was enriched, was used.

9(R,S)-HOTrE and 13(R,S)-HOTrE. 9-oxo-10(*E*),12(*Z*),15(*Z*)-octadecatrienoic (23 µmol; purchased from Larodan Co.) was dissolved in 2 mL of methanol and treated at 0°C with 15 mg of $NaBH_4$. The product was purified by SP-HLC using 2-propanol-hexane-acetic acid (1.5:98.5:0.01, v/v/v) as the mobile phase. The purity of the resulting 9(*R*,*S*)-HOTrE (about 15 µmol) was verified by SP-HPLC. The UV spectrum showed λ_{max} (EtOH) = 235 nm and the mass spectrum of the methyl ester/Me_3Si derivative showed prominent ions at *m/z* 380 (M^+), 311 (M^+ - CH_2-CH=CH-C_2H_5), and 223 (Me_3SiO^+=CH-CH=CH-CH=CH-CH_2-CH=CH-C_2H_5). 13(*R*,*S*)-HOTrE was prepared in an analogous way starting with 13-oxo-9(*Z*),11(*E*),15(*Z*)-octadecatrienoic acid. Its UV spectrum showed λ_{max} (EtOH) = 234 nm and the mass spectrum of the methyl ester/Me_3Si derivative showed a prominent ion at *m/z* 311 (M^+ - CH_2-CH=CH-C_2H_5) and weaker ions at 380 (M^+) and 290 (M^+ - Me_3SiOH). Enantiopure 9(*S*)-HOTrE and 13(*S*)-HOTrE were purchased from Larodan Co.

Method costs. The expense of purchasing analytical standards is a concern in establishing a new method. For the octadecanoid platform, the broadly estimated cost of purchasing the 47 currently commercially available standards is 11,500€ (including labeled IS). If the custom synthesized standards are costed at the same price as their commercially available structural analogs (*e.g.*, the price of *trans*-12,13-EpOME, commercially available, was used to estimate the price of *trans*-9,10-EpOME, not yet commercially available), the estimated cost for all standards is 19,500€ (including labeled IS). Based upon these costs, a minimum of 8500 calibration curves can be prepared (with the labeled IS as the limiting factor, which would need to be re-purchased every 10,000 samples) at a cost ~2.45€ per curve. While many standards are not yet commercially available, they are being actively produced by commercial companies and availability should increase in the near future. In the interim, all co-authors have agreed to make analytical quantities available for research purposes upon request.

Synthesis scheme A. Synthesis of 10-hydroxy-12(*Z*),15(*Z*)-octadecadienoic acid. *a*, n-butyllithium, BF_3-Et_2O; *b*, K_2CO_3, MeOH, THF; *c*, 1-bromo-2-pentyne, CuI, Cs_2CO_3, NaI; *d*, Lindlar catalyst, quinoline, H_2; *e*) NaOH, aq. MeOH. "TMS", trimethylsilyl.

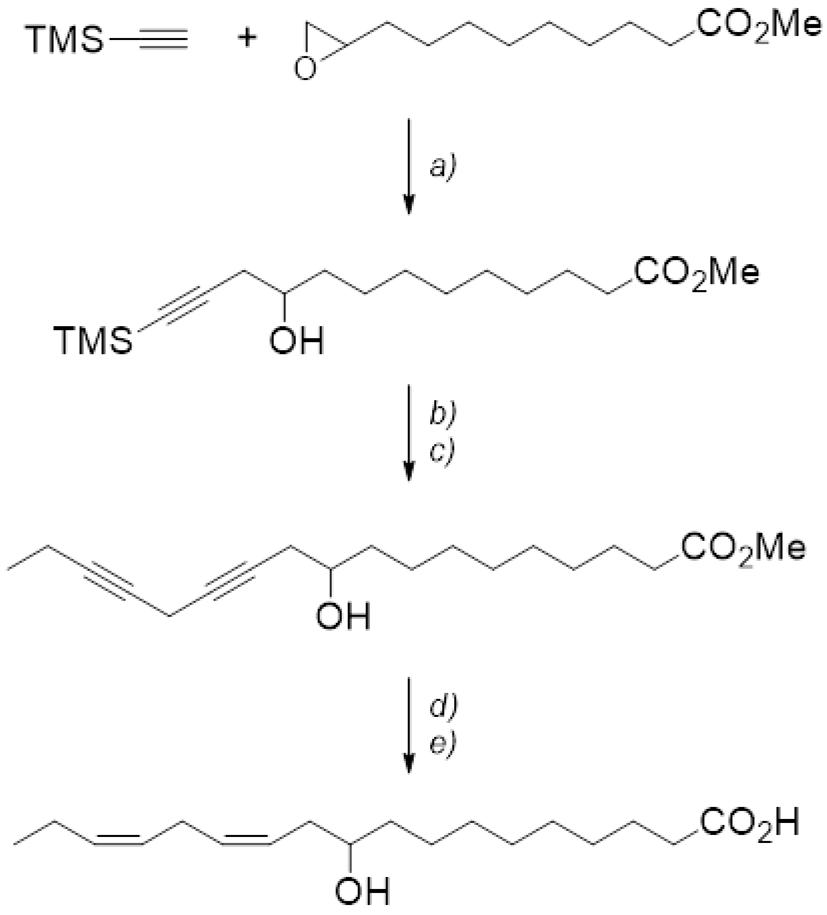

Synthesis scheme B. Methodology used for preparation of stereochemically defined TriHOMEs and TriHODEs. *a*, water-MeOH (99:1, v/v), pH 3; *b*, NaOH in THF-water. TriHOMEs, R_1 = $(CH_2)_4$-CH_3, R_2 = $(CH_2)_7$-COOH, or R_1 = $(CH_2)_7$-COOH, R_2 = $(CH_2)_4$-CH_3; TriHODEs, R_1 = CH_2-CH=CH-CH_2-CH_3, R_2 = $(CH_2)_7$-COOH, or R_1 = $(CH_2)_7$-COOH, R_2 = CH_2-CH=CH-CH_2-CH_3.

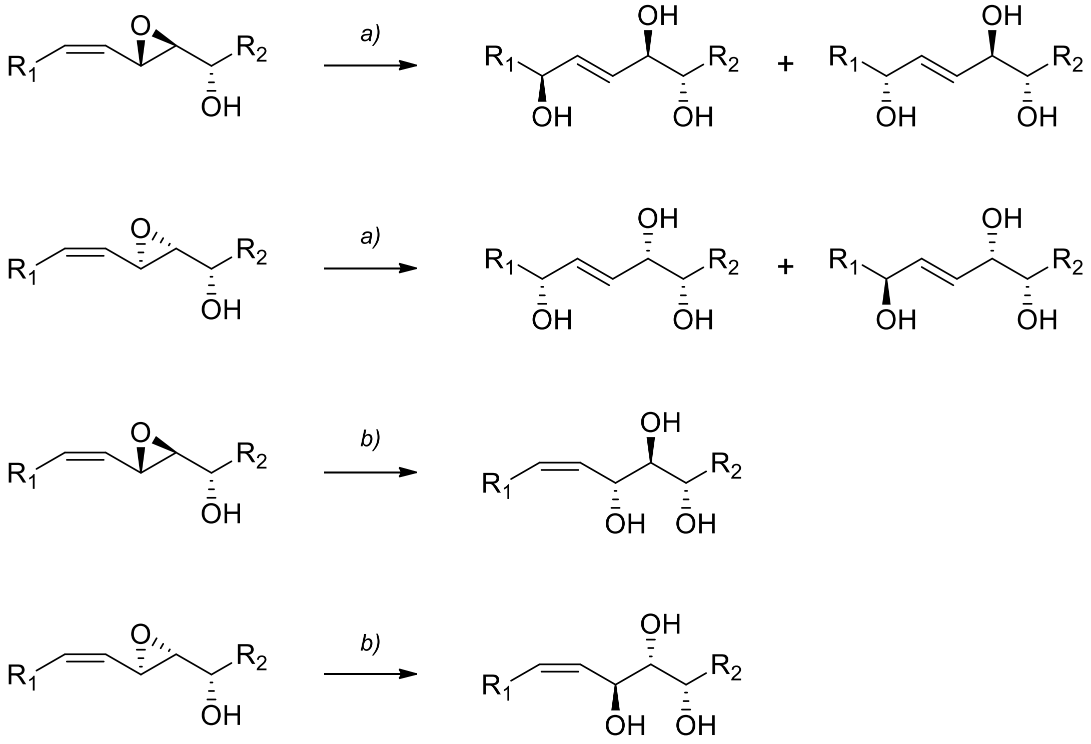

IV) Development of the chiral SFC method

Table S2. SFC method gradient

**Time (min)**	**%A**	**%B**	**Flow rate (mL/min)**	**ABPR (psi)**
0.0	95.0	5.0	2.0	2000
1.0	95.0	5.0	2.0	2000
11.0	75.0	25.0	2.0	2000
12.0	70.0	30.0	2.0	2000
12.3	70.0	30.0	1.5	2000
12.5	50.0	50.0	1.5	2000
14.5	50.0	50.0	1.5	2000
14.8	95.0	5.0	1.5	2000
15.2	95.0	5.0	2.0	2000
17.0	95.0	5.0	2.0	2000

Mobile phase A: supercritical CO_2
Mobile phase B: MeOH: EtOH 8:2, CH_3COOH 0.1% v/v
Column temperature: 35.0°C
Injection volume: 2.0µL
Make-up solvent: MeOH, CH_3COONH_4 5mM
Make-up solvent flow rate: 0.2mL/min

Conditioning procedure for Waters AMY-1 chiral column

The following procedure was applied to newly purchased AMY-1 chiral columns in order to tune the selectivity to harmonize the method performance and to obtain reproducible separations between new columns (Figure S3A) and the column previously in use (Figure S3D). The new column was first equilibrated as suggested by the manufacturer: 30 min with 100% CO_2 at a flow rate of 1.5 mL/min, followed by 30 min in CO_2:MeOH 1:1 at a flow rate of 1.5 mL/min, and subsequently equilibrated in 100% MeOH for 15 min at 0.5 mL/min and 15 min at 1 mL/min (**Step 0**). The column was then equilibrated at the method initial conditions for 30 minutes and a test sequence consisting of 3 solvent and 3 SST injections was performed and evaluated to benchmark the column performance (Figure S3A). The following step consisted in flowing through the column 5000 mL of CO_2:MeOH, CH_3COONH_4 5 mM 1:1 at a flow rate of 1.0 mL/min, setting the ABPR at 1500 psi (**Step 1**). This step required a total of 72 hours and was followed by evaluation, performed in the same way as described above (Figure S3B). The final step provided the fine-tuning resolution of more polar species and consisted of flowing 70 mL of ACN:IPA 1:1, HCOOH 0.2% v/v over 70 minutes at a flow rate of 1.0 mL/min (no CO_2, ABPR turned off; **Step 2**). The results were evaluated by the same test sequence (Figure S3C) and can be compared with a reference chromatogram acquired with the previous column (**Reference** panel), reported in Figure S3D.

To evaluate the reproducibility and stability of the procedure, three AMY-1 columns from different production lots were conditioned as described above. The procedure yielded the same results in terms of selectivity alteration on all the tested columns (data not shown).

Figure S3. Effects of conditioning on the AMY-1 stationary phase

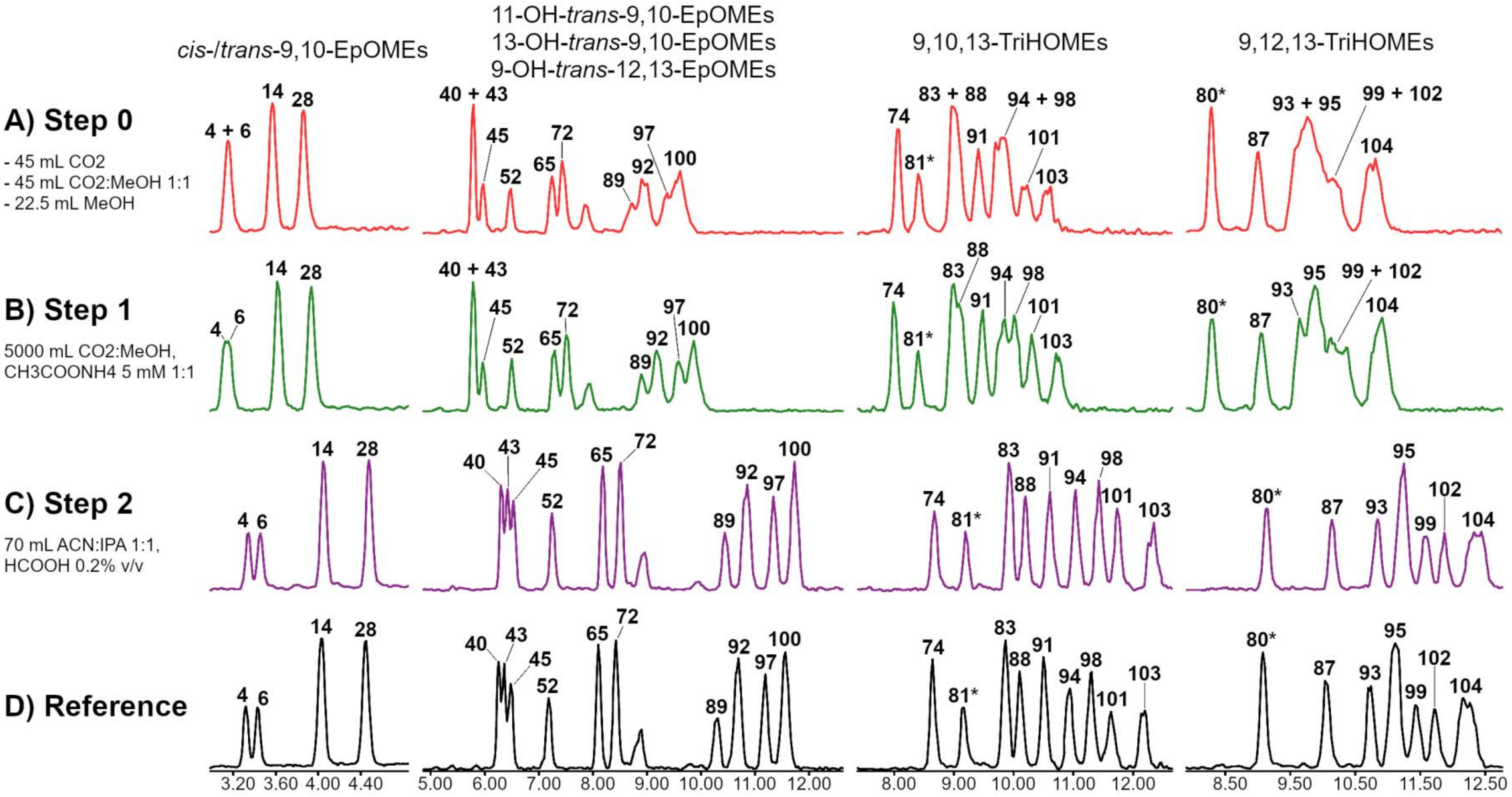

Figure S3. **Effects of conditioning on the AMY-1 stationary phase.** Evolution of the selectivity of new AMY-1 columns during the different steps of the initial conditioning procedure. The column could be used after Step 2 (purple trace). Panel D shows a reference chromatogram obtained on a different AMY-1 column, subjected to the same conditioning procedure. The procedure was tested on 3 AMY-1 columns from different production lots providing the same results. Refer to Table S1 for peak numbering and identity. Peaks labeled with * in the TriHOME panels refer to different positional isomers that share a transition with the illustrated TriHOMEs and appear in the same MRM channel.

Table S3. Solvent effect on chiral separation of octadecanoids

Mobile phase composition	% resolved species of the total # possible isomers			
**Best conditions**	**Epoxides**	**EHODEs**	**Diols**	**Triols**
MeOH, CH_3COOH 0.1% v/v	<u>82%</u>	<u>81%</u>	75%	**94%**
MeOH : EtOH 8:2, CH_3COOH 0.1% v/v	**86%**	**88%**	<u>80%</u>	<u>88%</u>
MeOH : ACN 1:1, CH_3COOH 0.1% v/v	64%	63%	**85%**	56%
MeOH : IPA 8:2, CH_3COOH 0.1% v/v	**86%**	<u>81%</u>	70%	75%
MeOH : ACN : IPA 8:1:1, CH_3COOH 0.1% v/v	**86%**	**88%**	70%	75%

The optimal result for each class of compounds is highlighted in **bold**, and the second best is <u>underlined</u>

All tests were performed without changing other parameters: ABPR 2000 psi, column T=35.0°C, gradient as reported in Table S2

The best result for each solvent composition is reported in the table; the full list of all the solvent compositions tested is reported below:

List of mobile phase B compositions tested:

MeOH (CH_3COOH 0.1% v/v)
MeOH : ACN 1:1 (CH_3COOH 0.1% v/v)
MeOH : ACN 8:2 (CH_3COOH 0.1% v/v)
MeOH : IPA 8:2 (CH_3COOH 0.1% v/v)
MeOH : IPA : ACN 6:2:2 (CH_3COOH 0.1% v/v)
MeOH : IPA : ACN 7:2:1 (CH_3COOH 0.1% v/v)
MeOH : IPA : ACN 7:1:2 (CH_3COOH 0.1% v/v)
MeOH : IPA : ACN 8:1:1 (CH_3COOH 0.5% v/v)
MeOH : IPA : ACN 8:1:1 (CH_3COOH 0.1% v/v)
MeOH : ACN : EtOH 8:1:1 (CH_3COOH 0.1% v/v)
MeOH : IPA : EtOH 8:1:1 (CH_3COOH 0.1% v/v)
MeOH : EtOH 9:1 (CH_3COOH 0.1% v/v)
MeOH : EtOH 8:2 (CH_3COOH 0.1% v/v)
MeOH : EtOH 7:3 (CH_3COOH 0.1% v/v)
MeOH : EtOH 6:4 (CH_3COOH 0.1% v/v)
MeOH : EtOH : IPA : ACN 6:2:1:1 (CH_3COOH 0.1% v/v)

The effect of different acid additives in the mobile phase and of the variation of other parameters such as the ABPR pressure, column temperature, as well as flow rate and composition of the make-up solvent were evaluated but not reported in the present work.

Figure S4. Solvent effect on chiral separation of octadecanoids

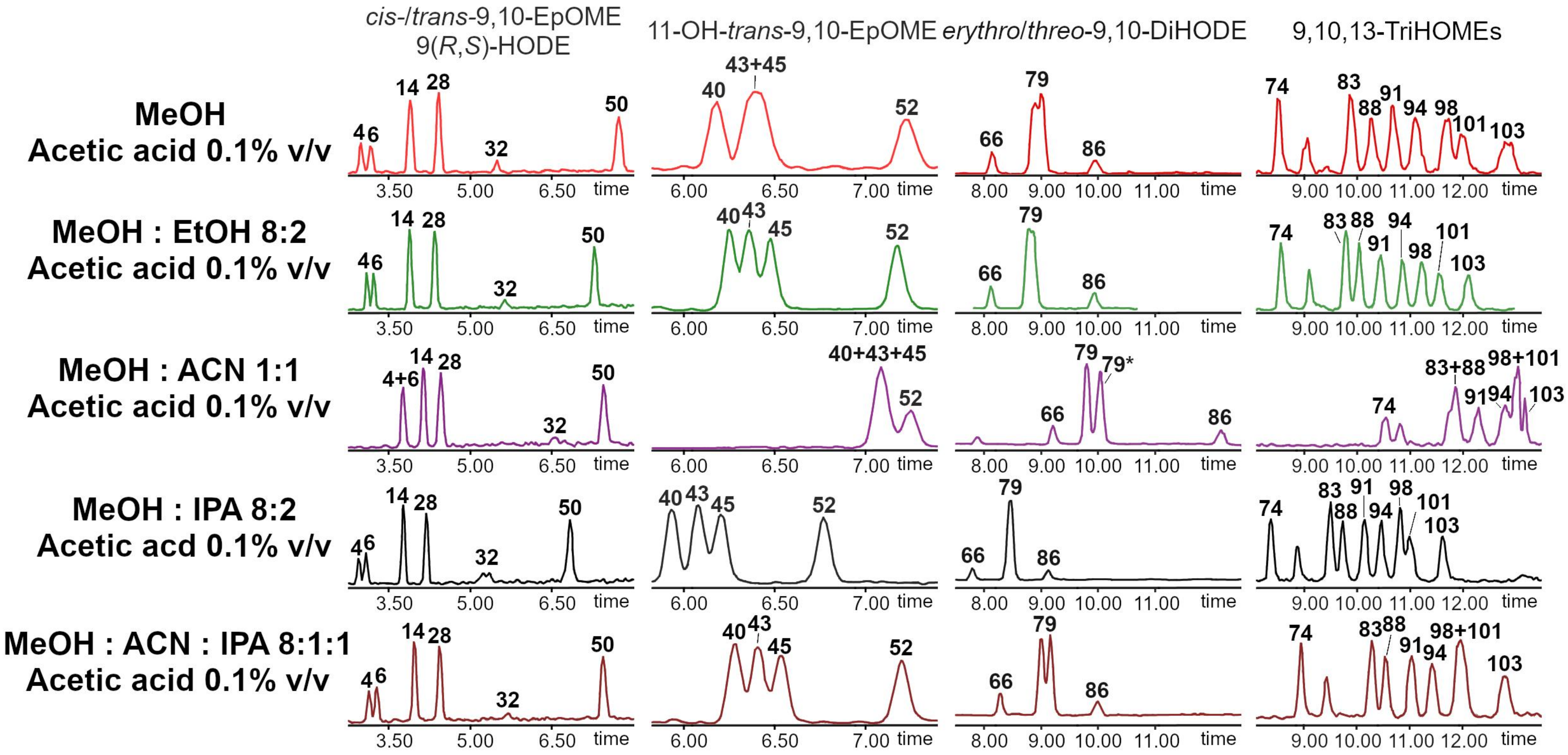

Figure S4. **Solvent effect on chiral separation of octadecanoids.** Comparison of different mobile phase compositions on the chromatographic resolution of octadecanoids on the AMY-1 column. Refer to Table S1 for peak numbering and identity. Expected peaks: *cis*-/*trans*-9,10-EpOME: 4; 9(*R*,*S*)-HODE: 2; 11-OH-*trans*-9,10-EpOME: 4; 9,10,13-TriHOME: 8. *Separation is not reported in Table S1 because a different solvent system was chosen for the final method.

Figure S5. AMY-1 stationary phase resolution upon 9,10- vs. 12,13-oxidized octadecanoids

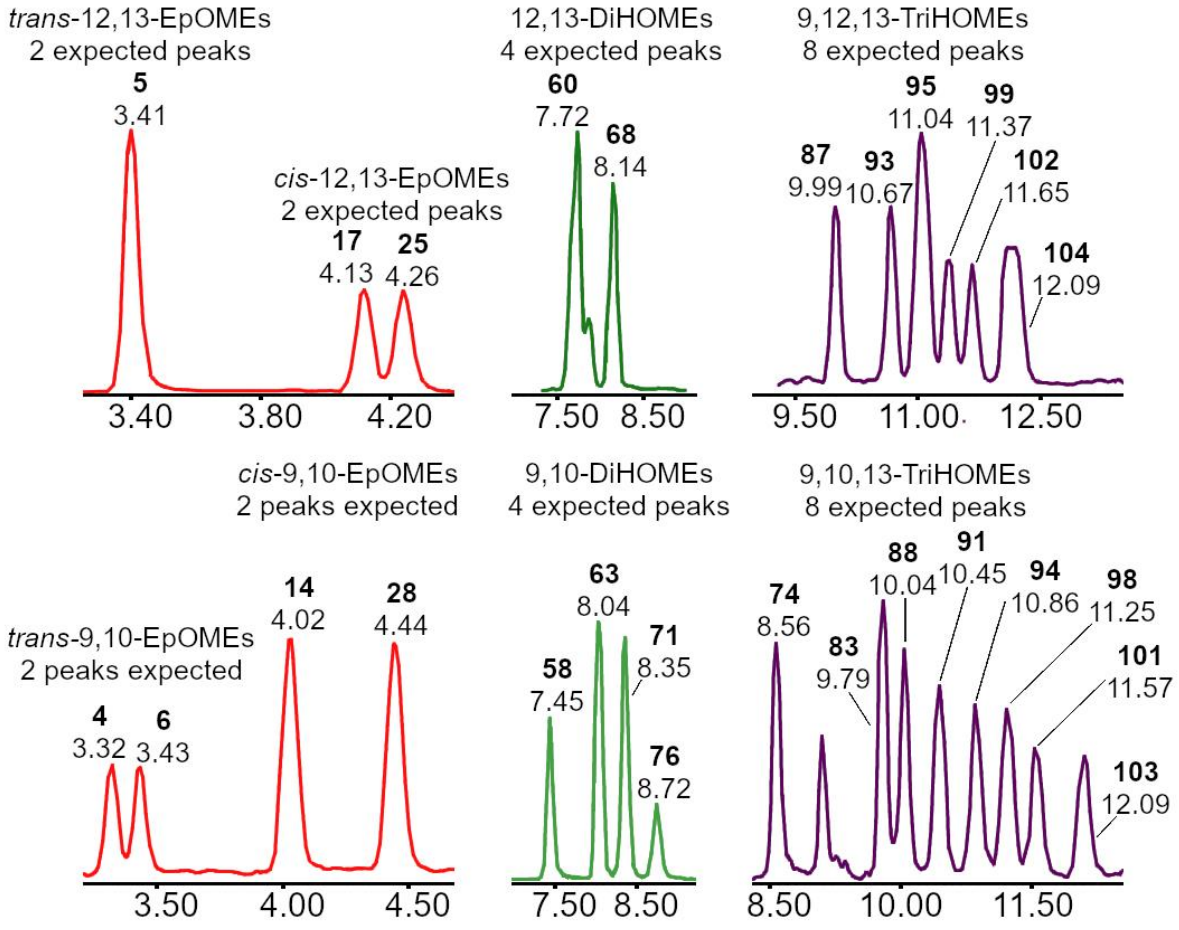

Figure S5. AMY-1 stationary phase resolution upon 9,10- vs. 12,13-oxidized octadecanoids. Comparison of the resolution of the AMY-1 stationary phase towards 12,13- (top panels) and 9,10- (bottom panels) oxidized octadecanoids. Chromatograms show illustrative LA metabolites: EpOMEs (red tracks), DiHOMEs (green tracks), and TriHOMEs (purple tracks). Peak identity and peak numbering are shown in Table S1.

Table S4. Method development parameters for the CEL-2 column

Time (min)	%A	%B	Flow rate (mL/min)	ABPR (psi)
0.0	97.0	3.0	2.0	2000
15.0	50.0	50.0	2.0	2000
16.0	50.0	50.0	2.0	2000
16.1	97.0	3.0	2.0	2000
18.0	97.0	3.0	2.0	2000

Mobile phase A: supercritical CO_2
Mobile phase B: EtOH : ACN 1:1, trifluoroacetic acid 0.2% v/v
Injection volume: 3.0µL
Column temperature: 50.0℃
Column: Waters Trefoil Cel-2, 3.0x150mm, 2.5µm
Make-up solvent: MeOH, NH_4OH 0.1% v/v
Make-up solvent flow rate: 0.25mL/min

Figure S6. Comparison of separation of 9,12,13-TriHOMEs on CEL-2 vs. AMY-1 columns

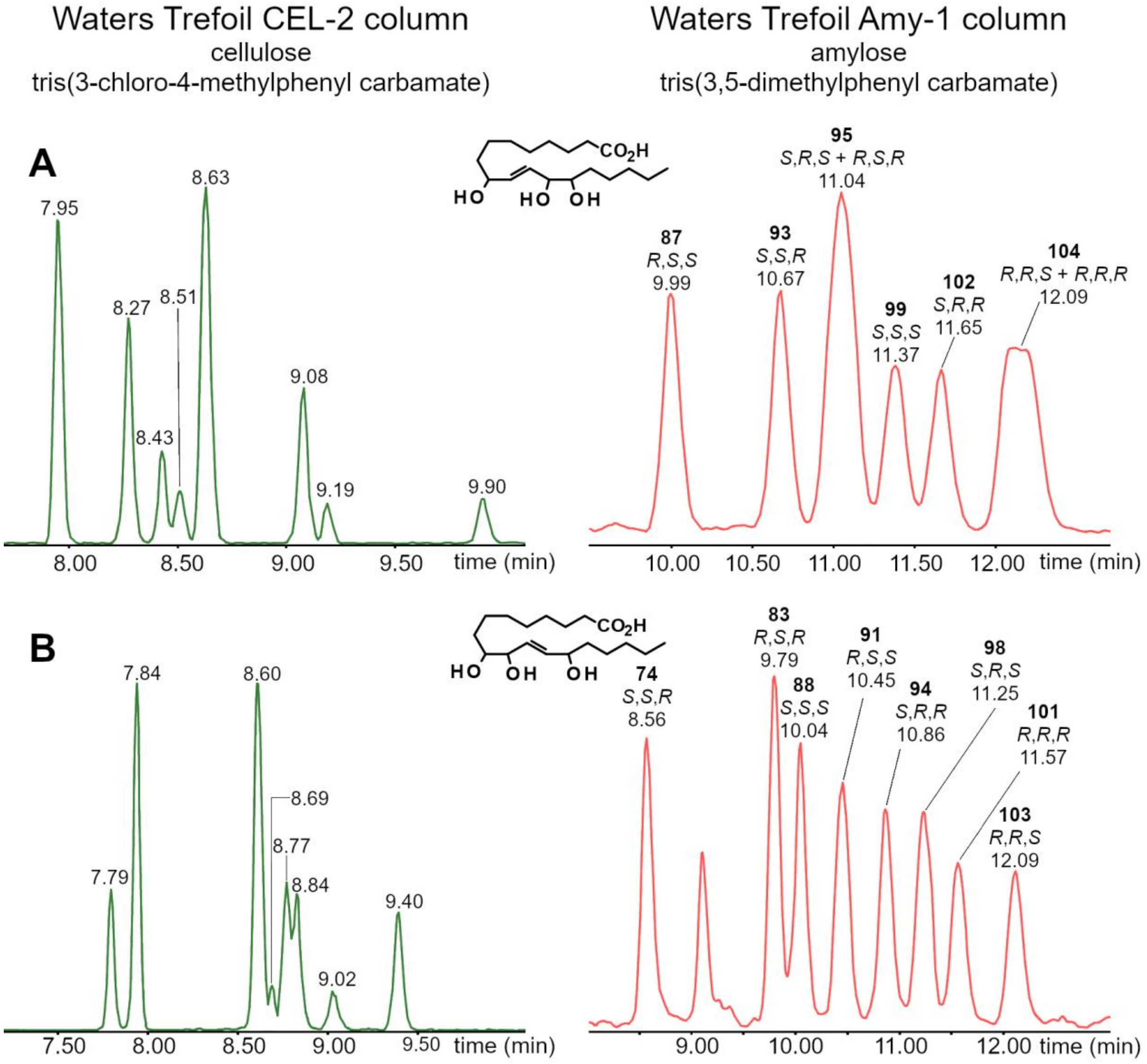

Figure S6. Comparison of separation of 9,12,13-TriHOMEs on CEL-2 vs. AMY-1 columns. Comparison of the chromatogram obtained for **A)** 9,12,13-TriHOME and **B)** 9,10,13-TriHOME with the CEL-2 column (cellulose-tris(3-chloro-4-methylphenyl carbamate), green tracks) and with the AMY-1 column (amylose-tris-3,5-dimethylphenyl carbamate), red tracks). Chromatograms were acquired with the methods described in Table S4 (CEL-2) and in Table S2 (AMY-1). For each TriHOME, 8 diastereoisomer peaks can be expected. Refer to Table S1 for peak numbering and identity for the AMY-1 chromatograms. The order of elution was not determined in the CEL-2 chromatograms. Different concentrations were injected in the two examples.

Table S5. SFC-MS/MS method parameters

**Compound**	**Retention time (min)**	**MRM transition (Q1 > Q3)**	**Cone voltage (V)**	**Collision energy (eV)**	**Quantified on**
12(*Z*)-10-KOME	3.49	295.2 > 139.1	25	21	Standard
12(*Z*),15(*Z*)-10-KODE	3.72	293.2 > 199.1	25	20	Standard
11(*E*),15(*Z*)-10-KODE	3.87	293.2 > 199.1	25	20	Standard
9-KODE	4.13	293.2 > 185.1	25	20	Standard
13-KOTrE	4.22	291.2 > 111.1	25	20	Standard
13-KODE	4.23	293.2 > 113.1	25	20	Standard
11(*E*)-10-KOME	4.24	295.2 > 139.1	25	21	Standard
9-KOTrE	4.32	291.2 > 1.85.1	25	17	Standard
13-KODE-d_3[a]	4.15	296.2 > 114.1	25	21	N.A.
trans-5,6-EpODE_1	2.89	293.2 > 137.1	25	13	Standard
trans-5,6-EpODE_2	3.01	293.2 > 137.1	25	13	Standard
cis-5,6-EpODE_1	3.22	293.2 > 137.1	25	13	Standard
cis-5,6-EpODE_2	3.58	293.2 > 137.1	25	13	Standard
trans-9,10-EpOME_1	3.32	295.2 > 171.1	25	18	*cis*-9,10-EpOME_1
trans-9,10-EpOME_2	3.43	295.2 > 171.1	25	18	*cis*-9,10-EpOME_2
cis-9(*S*),10(*R*)-EpOME	4.02	295.2 > 171.1	25	18	Standard
cis-9(*R*),10(*S*)-EpOME	4.44	295.2 > 171.1	25	18	Standard
cis-9(*S*),10(*R*)*-EpODE	3.93	293.2 > 171.1	25	15	*cis*-12,13-EpODE
cis-9(*R*),10(*S*)*-EpODE	4.17	293.2 > 171.1	25	15	*cis*-12,13-EpODE
trans-12,13-EpOME	3.41	295.2 > 195.1	25	17	Standard
cis-12(*R*),13(*S*)-EpOME	4.13	295.2 > 195.1	25	17	Standard
cis-12(*S*),13(*R*)-EpOME	4.26	295.2 > 195.1	25	17	Standard
cis-15,16-EpODE	3.84	293.2 > 235.1	25	13	*cis*-12,13-EpODE
trans-9,10-EpODA_1	3.90	297.2 > 171.1	28	19	Standard
trans-9,10-EpODA_2	4.04	297.2 > 171.1	28	19	Standard
cis-9(*S*),10(*R*)-EpODA	4.20	297.2 > 171.1	28	19	Standard
cis-9(*R*),10(*S*)-EpODA	4.46	297.2 > 171.1	28	19	Standard
cis-12,13-EpODE	4.34	293.2 > 183.1	25	15	Standard
9-oxo-*trans*-12,13-EpOME_1	7.44	309.2 > 209.1	25	11	Standard
9-oxo-*trans*-12,13-EpOME_2	8.12	309.2 > 209.1	25	11	Standard
cis-12,13-EpOME-d_4 [a]	4.18	299.2 > 198.1	25	16	N.A.
9(*R*)-HODE	5.60	295.2 > 171.1	25	18	9(*S*)-HODE
9(*S*)-HODE	7.10	295.2 > 171.1	25	18	Standard
12(*Z*)-10(*R*)*-HOME	5.61	297.2 > 185.1	25	20	Standard
12(*Z*)-10(*S*)*-HOME	5.93	297.2 > 185.1	25	20	Standard
13-OH-12-KOME	5.61	311.2 > 195.1	25	20	Standard

Compound	Retention time (min)	MRM transition (Q1 > Q3)	Cone voltage (V)	Collision energy (eV)	Quantified on
11(*E*),15(*Z*)-10(*R*)*-HODE	5.68	295.2 > 185.1	25	18	Standard
11(*E*),15(*Z*)-10(*S*)*-HODE	6.24	295.2 > 185.1	25	18	Standard
12(*Z*),15(*Z*)-10(*R*)*-HODE	6.08	295.2 > 185.1	25	18	Standard
12(*Z*)-15(*Z*)-10(*S*)*-HODE	7.44	295.2 > 185.1	25	18	Standard
9(*R*)-HOTrE	6.56	293.2 > 171.1	25	15	9(*S*)-HOTrE
9(*S*)-HOTrE	8.29	293.2 > 171.1	25	15	Standard
11(*E*)-10(*R*)*-HOME	6.45	297.2 > 185.1	25	20	Standard
11(*E*)-10(*S*)*-HOME	6.83	297.2 > 185.1	25	20	Standard
9(*S*)-HODE-d_4 [a]	6.93	299.2 > 172.1	25	16	N.A.
threo-9(*R*),10(*R*)-DiHOME	7.45	313.2 > 201.1	25	20	Standard
erythro-9,10-DiHOME_1	8.04	313.2 > 201.1	25	20	*threo*-9,10-DiHOME_1
threo-9(*R*),10(*R*)-DiHODE*	8.11	311.2 > 201.1	25	18	*erythro*-9,10-DiHODE_1
threo-9(*R*),10(*R*)-DiHODA*	8.85	315.2 > 171.1	25	18	Standard
threo_2/erythro_1-9,10-DiHODA	9.82	315.2 > 171.1	25	18	Standard
erythro-9,10-DiHODE	8.86	311.2 > 201.1	25	18	Standard
threo-9,10-DiHOME-d_4_1[a]	7.23	317.2 > 203.1	25	20	N.A.
erythro-9,10-DiHOME_2	8.35	313.2 > 201.1	25	20	*threo*-9,10-DiHOME_2
threo-9(*S*),10(*S*)-DiHODE*	9.92	311.2 > 201.1	25	18	*erythro*-9,10-DiHODE_1
threo-9(*S*),10(*S*)-DiHOME	8.72	313.2 > 201.1	25	20	Standard
erythro-9,10-DiHODA_2	10.30	315.2 > 171.1	25	18	Standard
threo-9,10-DiHOME-d_4_2[a]	8.48	317.2 > 203.1	25	20	N.A.
threo/erythro_1-12,13-DiHOME	7.72	313.2 > 183.1	25	20	Standard
erythro-12,13-DiHODE	7.80	311.2 > 213.1	25	20	Standard
erythro-12,13-DiHOME_2	8.14	313.2 > 183.1	25	20	*threo*-12,13-DiHOME
erythro-15,16-DiHODE	8.95	311.2 > 223.1	25	18	Standard
threo-12,13-DiHOME-d_4 [a]	7.42	317.2 > 185.1	25	22	N.A.
11-OH-*trans*-12,13-EpOME_1	6.09	311.2 > 197.1	25	17	Standard
11-OH-*trans*-12,13-EpOME_2	6.28	311.2 > 197.1	25	17	Standard
11-OH-*trans*-12,13-EpOME_3	7.12	311.2 > 197.1	25	17	Standard
11-OH-*trans*-12,13-EpOME_4	7.39	311.2 > 197.1	25	17	Standard
11-OH-*trans*-9,10-EpOME_1	6.24	311.2 > 171.1	25	17	Standard
11-OH-*trans*-9,10-EpOME_2	6.35	311.2 > 171.1	25	17	Standard
11-OH-*trans*-9,10-EpOME_3	6.47	311.2 > 171.1	25	17	Standard
11-OH-*trans*-9,10-EpOME_4	7.18	311.2 > 171.1	25	17	Standard
13-OH-*trans*-9,10-EpOME_1	8.08	311.2 > 171.1	25	20	Standard
13-OH-*trans*-9,10-EpOME_2	10.28	311.2 > 171.1	25	20	Standard
13-OH-*trans*-9,10-EpOME_3	10.64	311.2 > 171.1	25	20	Standard
9-OH-*trans*-12,13-EpOME_1	8.41	311.2 > 171.1	25	20	Standard

Compound	Retention time (min)	MRM transition (Q1 > Q3)	Cone voltage (V)	Collision energy (eV)	Quantified on
9-OH-*trans*-12,13-EpOME_2	11.15	311.2 > 171.1	25	20	Standard
9-OH-*trans*-12,13-EpOME_3	11.55	311.2 > 171.1	25	20	Standard
13-OH-*trans*-9,10-EpME-d_5 [a]	7.86	316.2 > 171.1	25	18	N.A.
13(*R*)-HOTrE	4.93	293.2 > 195.1	25	15	13(*S*)-HOTrE
13(*S*)-HOTrE	6.32	293.2 > 195.1	25	15	Standard
13(*R*)-HODE	5.01	295.2 > 195.1	25	17	13(*S*)-HODE
13(*S*)-HODE	8.07	295.2 > 195.1	25	17	Standard
13(*S*)-HOTrE-γ	6.83	293.2 > 193.1	25	13	Standard
13(*S*)-HODE-d_4 [a]	8.14	299.2 > 198.1	25	16	N.A.
9(*S*),10(*S*),13(*R*)-TriHOME	8.56	329.2 > 139.1	25	23	*9(S),10(S),13(S)-TriHOME*
9(*R*),10(*S*),13(*R*)-TriHOME	9.79	329.2 > 139.1	25	23	*9(S),10(S),13(S)-TriHOME*
9(*S*),10(*S*),13(*S*)-TriHOME	10.04	329.2 > 139.1	25	23	Standard
9(*R*),10(*S*),13(*S*)-TriHOME	10.45	329.2 > 139.1	25	23	*9(S),10(S),13(S)-TriHOME*
9(*S*),10(*R*),13(*R*)-TriHOME	10.86	329.2 > 139.1	25	23	*9(S),10(S),13(S)-TriHOME*
9(*S*),10(*R*),13(*S*)-TriHOME	11.25	329.2 > 139.1	25	23	*9(S),10(S),13(S)-TriHOME*
9(*R*),10(*R*),13(*R*)-TriHOME	11.57	329.2 > 139.1	25	23	*9(S),10(S),13(S)-TriHOME*
9(*R*),10(*R*),13(*S*)-TriHOME	12.09	329.2 > 139.1	25	23	*9(S),10(S),13(S)-TriHOME*
11(*R*),12(*S*),13(*S*)-TriHOME	9.03	329.2 > 199.1	25	23	Standard
9(*S*),10(*S*),11(*R*)-TriHOME	9.11	329.2 > 201.1	25	22	Standard
9(*S*),10(*S*),13(*S*)-TriHODE	9.22	327.2 > 85.1	25	22	Standard
9(*S*),10(*S*),11(*R*)-TriHODE	9.87	327.2 > 201.1	25	18	Standard
9(*R*),12(*S*),13(*S*)-TriHOME	9.99	329.2 > 211.1	25	23	*9(S),12(S),13(S)-TriHOME*
9(*S*),12(*S*),13(*R*)-TriHOME	10.67	329.2 > 211.1	25	23	*9(S),12(S),13(S)-TriHOME*
9,12,13-TriHOME (*S*,*R*,*S*+*R*,*S*,*R*)	11.04	329.2 > 211.1	25	23	*9(S),12(S),13(S)-TriHOME*
9(*S*),12(*S*),13(*S*)-TriHOME	11.37	329.2 > 211.1	25	23	Standard
9(*S*),12(*R*),13(*R*)-TriHOME	11.65	329.2 > 211.1	25	23	*9(S),12(S),13(S)-TriHOME*
9,12,13-TriHOME (*R*,*R*,*S*+*R*,*R*,*R*)	12.09	329.2 > 211.1	25	23	*9(S),12(S),13(S)-TriHOME*
9(*S*),12(*S*),13(*S*)-TriHODE	12.42	327.2 > 211.1	25	20	Standard
9(*S*),12(*S*),13(*S*)-$^{13}C_3$-TriHOME[a]	11.14	332.2 > 213.1	25	22	N.A.
9,10,11-TriHOME_1[b]	7.84	329.2 > 201.1	25	22	Qualitative screening
9,10,11-TriHOME_2+3[b]	8.04	329.2 > 201.1	25	22	Qualitative screening
9,10,11-TriHOME_4[b]	8.82	329.2 > 201.1	25	22	Qualitative screening
9,10,11-TriHOME_6[b]	9.25	329.2 > 201.1	25	22	Qualitative screening
9,10,11-TriHOME_7[b]	9.53	329.2 > 201.1	25	22	Qualitative screening
9,10,11-TriHOME_8[b]	9.86	329.2 > 201.1	25	22	Qualitative screening
11,12,13-TriHOME_1[b]	8.17	329.2 > 199.1	25	23	Qualitative screening
11,12,13-TriHOME_2[b]	8.37	329.2 > 199.1	25	23	Qualitative screening
11,12,13-TriHOME_4[b]	9.05	329.2 > 199.1	25	23	Qualitative screening

[a] Internal standards are highlighted in gray. Each compound is quantified with the internal standard that follows in the compound list.

[b] Not quantified, but only screened qualitatively. Standards of 9,10,11-TriHOME and 11,12,13 were synthesized, it was not possible to assign stereochemistry. In addition, full chromatographic resolution was not achieved for all isomers, which are therefore reported as peak numbers.

* Order of elution and identity not confirmed with an enantiopure standard but inferred by comparison with similar compounds under the same conditions.

N.A.=not applicable.

Table S6. SFC-MS/MS quantification parameters

Compound	LLOQ[a] (ng/mL)	ULOQ[b] (ng/mL)	Solvent-matched[c]			Matrix-matched[c]		
			slope	intercept	R^2	slope	intercept	R^2
12(*Z*)-10-KOME	0.600	600	0.0242	0.0076	0.998	0.0253	-0.0008	0.998
12(*Z*),15(*Z*)-10-KODE	0.200	200	0.1164	0.0127	0.999	0.1219	0.0008	0.997
11(*E*),15(*Z*)-10-KODE	0.200	200	0.1080	0.0075	0.999	0.1052	0.0041	0.998
9-KODE	0.200	200	0.1344	0.0285	0.998	0.1359	0.0219	0.998
13-KOTrE	0.200	200	0.0671	0.0092	0.999	0.0656	0.0044	0.997
13-KODE	0.200	200	0.1505	0.0473	0.999	0.1526	0.0575	0.997
11(*E*)-10-KOME	0.600	600	0.0236	0.0265	0.999	0.0242	0.0199	0.998
9-KOTrE	0.250	100	0.1190	0.0079	0.998	0.1229	0.0052	0.998
trans-5,6-EpODE_1	0.100	100	0.0508	-0.0008	0.998	0.0478	-0.0035	0.996
trans-5,6-EpODE_2	0.250	100	0.0542	0.0008	0.998	0.0536	-0.0126	0.997
cis-5,6-EpODE_1	0.500	100	0.0410	-0.0007	0.997	0.0329	-0.0053	0.996
cis-5,6-EpODE_2	0.200	100	0.0457	0.0023	0.997	0.0425	-0.0012	0.996
trans-9,10-EpOME_1	N.A.	N.A.	N.A.	N.A.	N.A.	N.A.	N.A.	N.A.
trans-9,10-EpOME_2	N.A.	N.A.	N.A.	N.A.	N.A.	N.A.	N.A.	N.A.
cis-9(*S*),10(*R*)-EpOME	0.250	100	0.2576	0.0044	0.997	0.2738	-0.0085	0.997
cis-9(*R*),10(*S*)-EpOME	0.250	100	0.2543	0.0014	0.998	0.2765	-0.0209	0.998
cis-9(*S*),10(*R*)*-EpODE	N.A.	N.A.	N.A.	N.A.	N.A.	N.A.	N.A.	N.A.
cis-9(*R*),10(*S*)*-EpODE	N.A.	N.A.	N.A.	N.A.	N.A.	N.A.	N.A.	N.A.
trans-12,13-EpOME	0.500	200	0.3228	-0.0070	0.996	0.3375	-0.0097	0.996
cis-12(*R*),13(*S*)-EpOME	0.200	200	0.1346	0.0076	0.998	0.1409	-0.0041	0.996
cis-12(*S*),13(*R*)-EpOME	0.200	200	0.1361	0.0126	0.999	0.1570	-0.0152	0.998
cis-15,16-EpODE	N.A.	N.A.	N.A.	N.A.	N.A.	N.A.	N.A.	N.A.
trans-9,10-EpODA_1	0.600	600	0.1059	0.0625	0.997	0.1159	0.0269	0.998
trans-9,10-EpODA_2	0.600	600	0.1076	0.1285	0.998	0.1214	0.0930	0.997
cis-9(*S*),10(*R*)-EpODA	1.000	1000	0.0656	0.0364	0.999	0.0704	-0.0014	0.997
cis-9(*R*),10(*S*)-EpODA	1.000	1000	0.0701	0.0775	0.998	0.0755	0.0301	0.997
cis-12,13-EpODE	0.250	100	0.1074	-0.0018	0.997	0.1145	-0.0009	0.997
9-oxo-*trans*-12,13-EpOME_1	0.250	100	0.2332	-0.0090	0.997	0.2487	-0.0121	0.997
9-oxo-*trans*-12,13-EpOME_2	0.500	100	0.2162	-0.0017	0.997	0.2395	-0.0405	0.998
9(*R*)-HODE	N.A.	N.A.	N.A.	N.A.	N.A.	N.A.	N.A.	N.A.
9(*S*)-HODE	0.250	100	0.0978	0.0141	0.998	0.1081	0.0133	0.998
12(*Z*)-10(*R*)*-HOME	0.100	100	0.1413	0.0041	0.997	0.1639	-0.0117	0.998
12(*Z*)-10(*S*)*-HOME	0.100	100	0.1429	0.0045	0.998	0.1639	-0.0048	0.998
13-OH-12-KOME	0.200	200	0.0638	0.0033	0.998	0.0743	-0.0160	0.998
11(*E*),15(*Z*)-10(*R*)*-HODE	0.075	30	0.1405	0.0013	0.996	0.1549	-0.0032	0.998
11(*E*),15(*Z*)-10(*S*)*-HODE	0.075	30	0.1279	0.0014	0.999	0.1485	-0.0055	0.998

Compound	LLOQ[a] (ng/mL)	ULOQ[b] (ng/mL)	Solvent-matched[c]			Matrix-matched[c]		
			slope	intercept	R^2	slope	intercept	R^2
12(*Z*),15(*Z*)-10(*R*)*-HODE	0.070	70	0.1725	0.0033	0.998	0.2001	-0.0133	0.998
12(*Z*)-15(*Z*)-10(*S*)*-HODE	0.070	70	0.1817	-0.0007	0.998	0.2132	-0.0081	0.998
9(*R*)-HOTrE	N.A.	N.A.	N.A.	N.A.	N.A.	N.A.	N.A.	N.A.
9(*S*)-HOTrE	0.100	100	0.0811	-0.0011	0.996	0.0865	-0.0042	0.998
11(*E*)-10(*R*)*-HOME	0.220	220	0.0415	0.0033	0.997	0.0469	-0.0109	0.998
11(*E*)-10(*S*)*-HOME	0.180	180	0.0436	-0.0004	0.998	0.0498	-0.0113	0.998
threo-9(*R*),10(*R*)-DiHOME	0.120	120	0.3192	0.0105	0.998	0.3560	-0.0050	0.998
erythro-9,10-DiHOME_1	N.A.	N.A.	N.A.	N.A.	N.A.	N.A.	N.A.	N.A.
threo-9(*R*),10(*R*)-DiHODE*	N.A.	N.A.	N.A.	N.A.	N.A.	N.A.	N.A.	N.A.
threo-9(*R*),10(*R*)-DiHODA*	5.000	1000	0.0237	0.0219	0.998	0.0270	-0.0224	0.998
threo_2/erythro_1-9,10-DiHODA	5.000	1000	0.0259	0.0167	0.997	0.0289	-0.0110	0.998
erythro-9,10-DiHODE	0.063	62.7	0.8475	0.0128	0.997	0.9676	-.0.157	0.998
erythro-9,10-DiHOME_2	N.A.	N.A.	N.A.	N.A.	N.A.	N.A.	N.A.	N.A.
threo-9(S),10(S)-DiHODE*	N.A.	N.A.	N.A.	N.A.	N.A.	N.A.	N.A.	N.A.
threo-9(S),10(S)-DiHOME	0,200	80	0.3113	0.0088	0.998	0.3283	-0.0006	0.999
erythro-9,10-DiHODA_2	5.000	1000	0.0299	0.0276	0.999	0.0308	0.0056	0.999
threo/erythro_1-12,13-DiHOME	0.200	200	0.1478	0.0083	0.999	0.1598	-0.0068	0.998
erythro-12,13-DiHODE	0.285	28.5	0.1017	-0.0005	0.997	0.1121	-0.0061	0.998
erythro-12,13-DiHOME_2	N.A.	N.A.	N.A.	N.A.	N.A.	N.A.	N.A.	N.A.
erythro-15,16-DiHODE	0.252	100.7	0.1909	-0.0059	0.997	0.1121	-0.0061	0.998
11-OH-*trans*-12,13-EpOME_1	0.840	840	0.0822	0.0122	0.998	0.0920	-0.0294	0.998
11-OH-*trans*-12,13-EpOME_2	0.360	360	0.1105	0.0107	0.998	0.1274	-0.0395	0.998
11-OH-*trans*-12,13-EpOME_3	0.360	360	0.0997	0.0031	0.998	0.1051	-0.0179	0.998
11-OH-*trans*-12,13-EpOME_4	0.840	840	0.0869	-0.0025	0.998	0.0872	-0.0586	0.998
11-OH-*trans*-9,10-EpOME_1	0.375	150	0.3262	-0.0039	0.998	0.3411	-0.0102	0.998
11-OH-*trans*-9,10-EpOME_2	0.375	150	0.3616	-0.0058	0.997	0.3809	-0.0369	0.996
11-OH-*trans*-9,10-EpOME_3	0.375	150	0.3271	0.0125	0.998	0.3583	-0.0275	0.998
11-OH-*trans*-9,10-EpOME_4	0.375	150	0.3280	-0.0062	0.998	0.3410	-0.0293	0.998
13-OH-*trans*-9,10-EpOME_1	0.900	900	0.1088	0.0199	0.997	0.1166	-0.0237	0.996
13-OH-*trans*-9,10-EpOME_2	1.500	600	0.1213	0.0084	0.997	0.1325	-0.0377	0.998
13-OH-*trans*-9,10-EpOME_3	1.500	1500	0.1056	0.0365	0.998	0.1151	-0.0481	0.998
9-OH-*trans*-12,13-EpOME_1	1.200	1200	0.0739	0.0274	0.998	0.0797	-0.0251	0.998
9-OH-*trans*-12,13-EpOME_2	3.000	1200	0.0653	0.0166	0.998	0.0711	-0.0279	0.998
9-OH-*trans*-12,13-EpOME_3	4.000	1600	0.0769	0.0240	0.998	0.0832	-0.0680	0.998
13(*R*)-HOTrE	N.A.	N.A.	N.A.	N.A.	N.A.	N.A.	N.A.	N.A.
13(*S*)-HOTrE	0.500	100	0.0468	0.0002	0.998	0.0526	-0.0091	0.998
13(*R*)-HODE	N.A.	N.A.	N.A.	N.A.	N.A.	N.A.	N.A.	N.A.
13(*S*)-HODE	0.200	200	0.1227	0.0198	0.998	0.1324	0.0212	0.998

**Compound**	**LLOQ[a] (ng/mL)**	**ULOQ[b] (ng/mL)**	**Solvent-matched[c]**			**Matrix-matched[c]**		
			**slope**	**intercept**	**R^2**	**slope**	**intercept**	**R^2**
13(*S*)-HOTrE-γ	0.250	100	0.1015	-0.0021	0.997	0.1101	-0.0136	0.998
9(*S*),10(*S*),13(*R*)-TriHOME	N.A.	N.A.	N.A.	N.A.	N.A.	N.A.	N.A.	N.A.
9(*R*),10(*S*),13(*R*)-TriHOME	N.A.	N.A.	N.A.	N.A.	N.A.	N.A.	N.A.	N.A.
9(*S*),10(*S*),13(*S*)-TriHOME	0.600	600	0.0336	0.0196	0.997	0.0371	-0.0184	0.998
9(*R*),10(*S*),13(*S*)-TriHOME	N.A.	N.A.	N.A.	N.A.	N.A.	N.A.	N.A.	N.A.
9(*S*),10(*R*),13(*R*)-TriHOME	N.A.	N.A.	N.A.	N.A.	N.A.	N.A.	N.A.	N.A.
9(*S*),10(*R*),13(*S*)-TriHOME	N.A.	N.A.	N.A.	N.A.	N.A.	N.A.	N.A.	N.A.
9(*R*),10(*R*),13(*R*)-TriHOME	N.A.	N.A.	N.A.	N.A.	N.A.	N.A.	N.A.	N.A.
9(*R*),10(*R*),13(*S*)-TriHOME	N.A.	N.A.	N.A.	N.A.	N.A.	N.A.	N.A.	N.A.
11(*R*),12(*S*),13(*S*)-TriHOME	0.500	200	0.0494	0.0083	0.996	0.0563	-0.0076	0.998
9(*S*),10(*S*),11(*R*)-TriHOME	0.600	600	0.0724	0.0150	0.996	0.0766	-0.0287	0.998
9(*S*),10(*S*),13(*S*)-TriHODE	1.000	1000	0..211	0.0094	0.997	0.0224	-0.0074	0.997
9(*S*),10(*S*),11(*R*)-TriHODE	15.000	600	0.0028	0.0058	0.999	0.0030	-0.0113	0.996
9(*R*),12(*S*),13(*S*)-TriHOME	N.A.	N.A.	N.A.	N.A.	N.A.	N.A.	N.A.	N.A.
9(*S*),12(*S*),13(*R*)-TriHOME	N.A.	N.A.	N.A.	N.A.	N.A.	N.A.	N.A.	N.A.
9,12,13-TriHOME (*S*,*R*,*S*+*R*,*S*,*R*)	N.A.	N.A.	N.A.	N.A.	N.A.	N.A.	N.A.	N.A.
9(*S*),12(*S*),13(*S*)-TriHOME	0.600	600	0.0569	0.0325	0.998	0.0612	-0.0107	0.998
9(*S*),12(*R*),13(*R*)-TriHOME	N.A.	N.A.	N.A.	N.A.	N.A.	N.A.	N.A.	N.A.
9,12,13-TriHOME (*R*,*R*,*S*+*R*,*R*,*R*)	N.A.	N.A.	N.A.	N.A.	N.A.	N.A.	N.A.	N.A.
9(*S*),12(*S*),13(*S*)-TriHODE	0.600	600	0.0355	0.0180	0.997	0.0372	-0.0080	0.998

* Order of elution and identity not confirmed with an enantiopure standard but inferred by comparison with similar compounds under the same conditions.

[a] LLOQ: lowest limit of quantification. Lowest standard used to build the curve model; accepted if S/N>10 and deviation on the backcalculated standard concentration <20%.

[b] ULOQ: upper limit of quantification. Highest calibrant used to build the curve model.

[c] Slopes and intercepts calculated as average of three curves injected three times.

N.A.=not applicable: the compound is quantified on the curve built on the closest isomer (see Table S5).

V) Reversed phase LC method development

Table S7. LC method gradient

Time (min)	%A	%B	Flow rate (mL/min)
0.0	65.0	35.0	0.45
2.1	60.0	40.0	0.45
3.5	58.0	42.0	0.45
5.5	50.0	50.0	0.45
11.5	35.0	65.0	0.45
13.0	27.5	72.5	0.45
15.0	20.0	80.0	0.45
15.1	0.0	100.0	0.45
17.0	0.0	100.0	0.45
17.1	65.0	35.0	0.45
19.0	65.0	35.0	0.45

Mobile phase A: H_2O, CH_3COOH 0.1% v/v
Mobile phase B: ACN : IPA 9:1
Injection volume: 5.0µL
Column temperature: 60.0°C

Figure S7. Overlaid chromatogram for the LC separation of octadecanoids

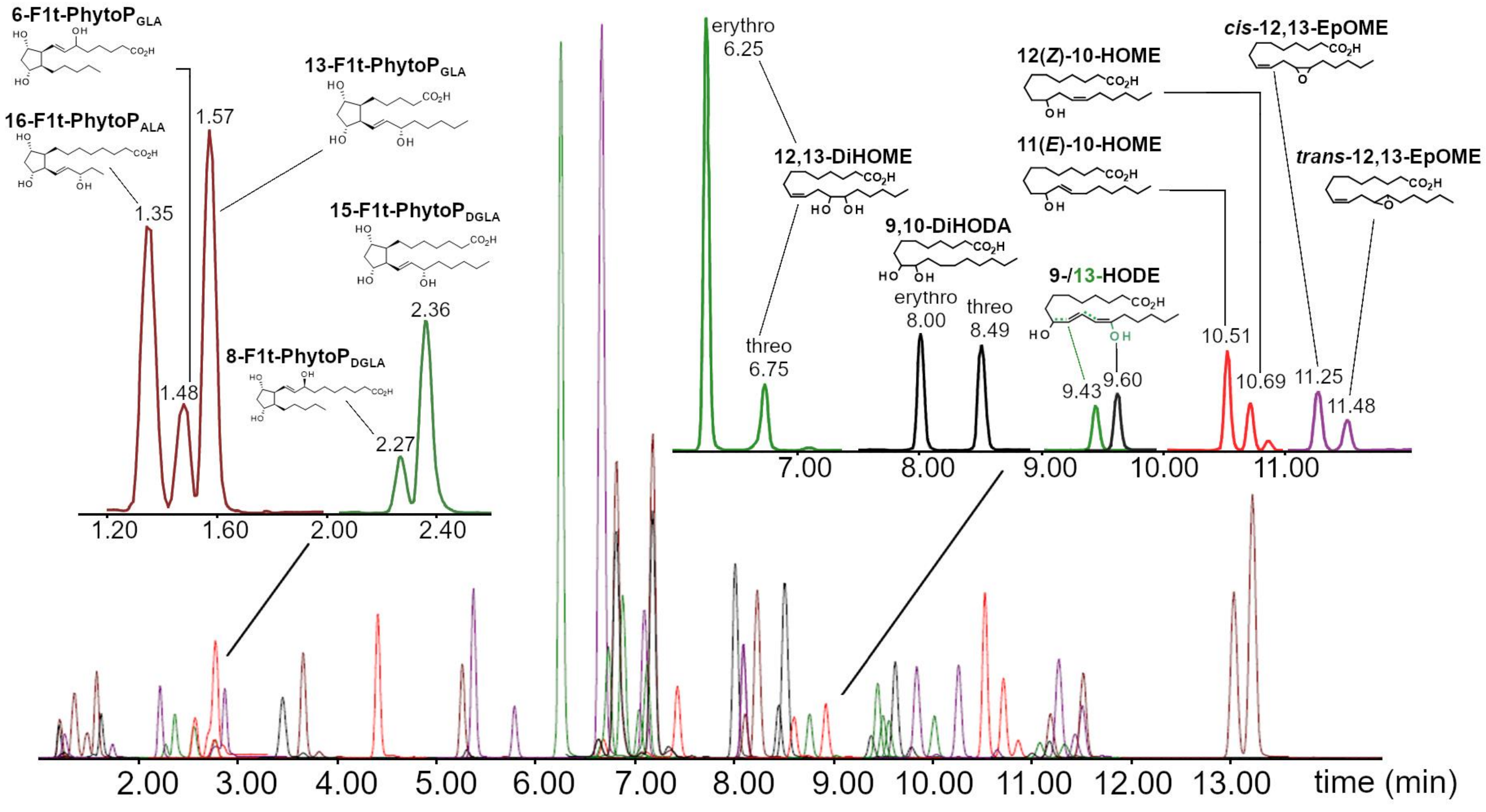

Figure S7. Overlaid chromatogram and resolution features for the LC separation of octadecanoids. Overlaid LC chromatogram of all the acquired MRM transitions with zoom panels showing the separation of isomeric features including *erythro*/*threo* diols, *cis*/*trans* epoxides, and *E*/*Z* double bonds, as well as the position of -OH groups and double bonds in PhytoPs.

Table S8. LC-MS/MS method parameter

Compound	Retention time (min)	MRM transition (Q1 > Q3)	Cone Voltage (V)	Collision energy (eV)	Quantified on
ent-9-12-*epi*-ST- Δ^{10}-13-PhytoF	1.20	343.4 > 237.2	25	20	Standard
ent-16-13-*epi*-ST- Δ^{14}-9-PhytoF	1.21	343.5 > 201.2	25	23	Standard
16-F_{2t}-$PhytoP_{SDA}$	1.21	325.5 > 207.2	25	18	Standard
6-F_{2t}-$PhytoP_{SDA}$	1.25	325.3 > 129.1	25	18	Standard
16-F_{1t}-$PhytoP_{ALA}$	1.34	327.2 > 225.2	25	23	Standard
9-F_{1t}-$PhytoP_{ALA}$	1.37	327.3 > 171.3	25	23	Standard
6-F_{1t}-$PhytoP_{GLA}$	1.48	327.4 > 129.1	25	20	Standard
13-F_{1t}-$PhytoP_{GLA}$	1.57	327.2 > 283.2	25	18	Standard
ent-9-D_{1t}-$PhytoP_{ALA}$	1.62	325.3 > 289.2	25	12	Standard
8-F_{1t}-$PhytoP_{DGLA}$	2.27	355.5 > 157.1	25	23	Standard
15-F_{1t}-$PhytoP_{DGLA}$	2.36	355.3 > 293.2	25	20	Standard
9-L_1-$PhytoP_{ALA}$	2.55	307.3 > 185.1	25	18	Standard
16-B_1-$PhytoP_{ALA}$	2.56	307.2 > 235.2	25	18	Standard
16-F_{1t}-PhytoP-C_{19} [a]	1.73	341.3 > 279.2	25	23	N.A.
9,12,13-TriHODE	2.21	327.2 > 211.1	25	20	Standard
9,10,13-TriHODE	2.23	327.2 > 85.1	25	22	Standard
9,10,11-TriHODE	2.76	327.2 > 201.1	25	18	Standard
9,12,13-TriHOME	2.77	329.2 > 211.1	25	22	Standard
9,10,13-TriHOME	2.86	329.2 > 139.1	25	23	Standard
11,12,13-TriHOME	3.44	329.2 > 199.1	25	23	Standard
9,10,11-TriHOME	3.65	329.2 > 201.1	25	22	Standard
9(*S*),12(*S*),13(*S*)-$^{13}C_3$-TriHOME[a]	2.77	332.2 > 213.1	25	22	N.A.
13-OH-*trans*-9,10-EpOME	6.62	311.2 > 171.1	25	20	Standard
11-OH-*trans*-9,10-EpOME _1	6.81	311.2 > 201.1	25	17	Standard
11-OH-*trans*-9,10-EpOME _2	7.17	311.2 > 201.1	25	17	Standard
11-OH-*trans*-12,13-EpOME_1	6.86	311.2 > 197.1	25	17	Standard
11-OH-*trans*-12,13-EpOME_2	7.11	311.2 > 197.1	25	17	Standard
9-OH-*trans*-12,13-EpOME	7.33	311.2 > 171.1	25	20	Standard
13-OH-*trans*-9,10-EpOME-d_5[a]	6.59	316.2 > 171.1	25	18	N.A.
8(*R*),11(*S*)-DiHODE	4.40	311.2 > 293.1	25	15	Standard
erythro-15,16-DiHODE	5.25	311.2 > 223.1	25	18	Standard
threo-15,16-DiHODE	5.68	311.2 > 223.1	25	18	Erythro isomer
erythro-12,13-DiHODE	5.30	311.2 > 213.1	25	20	Standard
threo-12,13-DiHODE	5.75	311.2 > 213.1	25	20	Erythro isomer
erythro-12,13-DiHOME	6.25	313.2 > 183.1	25	20	Standard
threo-12,13-DiHOME	6.75	313.2 > 183.1	25	20	Standard

Compound	Retention time (min)	MRM transition (Q1 > Q3)	Cone Voltage (V)	Collision energy (eV)	Quantified on
threo-12,13-DiHOME-d_4[a]	6.68	317.2 > 185.1	25	22	N.A.
erythro-9,10-DiHODE	5.37	311.2 > 201.1	25	18	Standard
threo-9,10-DiHODE	5.78	311.2 > 201.1	25	18	Standard
erythro-9,10-DiHOME	6.65	313.2 > 201.1	25	20	Standard
threo-9,10-DiHOME	7.09	313.2 > 201.1	25	20	Standard
erythro-9,10-DiHODA	8.00	315.2 > 171.1	25	25	Standard
threo-9,10-DiHODA	8.49	315.2 > 171.1	25	25	Standard
threo-9,10-DiHOME-d_4[a]	7.04	317.2 > 203.1	25	20	N.A.
13-OH-12-KOME	7.42	311.2 > 195.1	25	20	Standard
13-HOTrE	8.21	293.2 > 195.1	25	15	Standard
13-HOTrE-γ	8.43	293.2 > 193.1	25	13	Standard
13-HODE	9.43	295.2 > 195.1	25	17	Standard
13-HODE-d_4[a]	9.37	299.2 > 198.1	25	16	N.A.
9-HOTrE	8.10	293.2 > 171.1	25	15	Standard
12(*Z*),15(*Z*)/11(*E*),15(*Z*)-10-HODE	8.91	295.2 > 185.1	25	18	Standard
9-HODE	9.60	295.2 > 171.1	25	18	Standard
12(*Z*)-10-HOME	10.51	297.2 > 185.1	25	20	Standard
11(*E*)-10-HOME	10.69	297.2 > 185.1	25	20	Standard
9-HODE-d_4[a]	9.54	299.2 > 172.1	25	16	N.A.
13-KOTrE	8.59	291.2 > 111.1	25	20	Standard
9-KOTrE	8.74	291.2 > 185.1	25	17	Standard
12(*Z*),15(*Z*)/11(*E*),15(*Z*)-10-KODE	9.49	293.2 > 199.1	25	20	Standard
13-KODE	9.83	293.2 > 113.1	25	20	Standard
9-KODE	10.24	293.2 > 185.1	25	20	Standard
12(*Z*)-10-KOME	11.06	295.2 > 139.1	25	21	Standard
11(*E*)-10-KOME	11.30	295.2 > 139.1	25	21	Standard
13-KODE-d_3[a]	9.78	296.2 > 114.1	25	21	N.A.
9-oxo-*trans*-12,13-EpOME	8.08	309.2 > 209.1	25	21	Standard
cis-15,16-EpODE	9.64	293.2 > 235.1	25	16	*cis*-12,13-EpOME
cis-12,13-EpODE	10.00	293.2 > 183.1	25	15	Standard
cis-12,13-EpOME	11.25	295.2 > 195.1	25	17	Standard
trans-12,13-EpOME	11.48	295.2 > 195.1	25	17	Standard
cis-12,13-EpOME-d_4[a]	11.17	299.2 > 198.1	25	16	N.A.
cis-9,10-EpODE	9.79	293.2 > 171.1	25	15	*cis*-12,13-EpOME
cis-5,6-EpODE	10.98	293.2 > 137.1	25	13	Standard
trans-5,6-EpODE	11.15	293.2 > 137.1	25	13	Standard
cis-9,10-EpOME	11.49	295.2 > 171.1	25	18	Standard
trans-9,10-EpOME	11.79	295.2 > 171.1	25	18	*cis*-9,10-EpOME

Compound	Retention time (min)	MRM transition (Q1 > Q3)	Cone Voltage (V)	Collision energy (eV)	Quantified on
cis-9,10-EpODA	13.01	297.2 > 171.1	25	19	Standard
trans-9,10-EpODA	13.20	297.2 > 171.1	25	19	Standard
cis-9,10-EpOME-d_4[a]	11.42	299.2 > 172.1	25	16	N.A.

[a] Internal standards are highlighted in gray. Each compound is quantified with the internal standard that follows in the compound list.

N.A.=not applicable.

Table S9. LC-MS/MS quantification parameters

Compound	LLOQ[a] (ng/mL)	ULOQ[b] (ng/mL)	Solvent-matched[c]			Matrix-matched[c]		
			slope	intercept	R^2	slope	intercept	R^2
ent-9-12-*epi*-ST- Δ^{10}-13-PhytoF	0.100	100	0.0303	0.0012	0.998	0.0296	0.0010	0.997
ent-16-13-*epi*-ST- Δ^{14}-9-PhytoF	0.050	25	0.0749	0.0014	0.998	0.0704	0.0024	0.997
16-F_{2t}-$PhytoP_{SDA}$	0.250	100	0.0035	0.0018	0.998	0.0032	0.0026	0.997
6-F_{2t}-$PhytoP_{SDA}$	0.050	50	0.0516	0.0015	0.998	0.0460	0.0022	0.997
16-F_{1t}-$PhytoP_{ALA}$	0.125	100	0.0289	0.0025	0.998	0.0279	0.0029	0.997
9-F_{1t}-$PhytoP_{ALA}$	0.050	100	0.0467	0.0016	0.998	0.0432	0.0019	0.998
6-F_{1t}-$PhytoP_{GLA}$	0.050	100	0.0362	0.0010	0.998	0.0332	0.0013	0.997
13-F_{1t}-$PhytoP_{GLA}$	0.050	100	0.0582	0.0013	0.998	0.0535	0.0040	0.998
ent-9-D_{1t}-$PhytoP_{ALA}$	0.010	40	0.1807	0.0010	0.999	0.1797	0.0005	0.999
8-F_{1t}-$PhytoP_{DGLA}$	0.025	50	0.0441	0.0006	0.998	0.0423	0.0010	0.998
15-F_{1t}-$PhytoP_{DGLA}$	0.100	100	0.0370	0.0043	0.998	0.0331	0.0011	0.999
9-L_1-$PhytoP_{ALA}$	0.020	20	0.1712	0.0010	0.998	0.1692	0.0022	0.998
16-B_1-$PhytoP_{ALA}$	0.025	100	0.0695	0.0009	0.999	0.0708	0.0018	0.999
9,12,13-TriHODE	0.050	200	0.0305	-0.0002	0.999	0.0300	-0.0001	0.998
9,10,13-TriHODE	0.025	100	0.0219	0.0000	0.999	0.0209	0.0001	0.999
9,10,11-TriHODE	1.250	1000	0.0023	-0.0001	0.999	0.0022	0.0000	0.999
9,12,13-TriHOME	0.050	100	0.0655	0.0058	0.999	0.0576	0.0180	0.999
9,10,13-TriHOME	0.050	200	0.0440	0.0020	0.999	0.0397	0.0091	0.999
11,12,13-TriHOME	0.050	200	0.0333	-0.0003	0.999	0.0329	-0.0001	0.998
9,10,11-TriHOME	0.050	200	0.0549	-0.0008	0.999	0.0538	-0.0003	0.998
13-OH-*trans*-9,10-EpOME	0.300	150	0.1073	0.0174	0.999	0.1041	0.0180	0.999
11-OH-*trans*-9,10-EpOME _1	0.150	600	0.1141	-0.0011	0.998	0.0899	0.0149	0.997
11-OH-*trans*-9,10-EpOME _2	0.150	600	0.1477	-0.0005	0.999	0.1205	0.0159	0.999
11-OH-*trans*-12,13-EpOME_1	0.250	1000	0.2123	0.0523	0.999	0.1921	0.0313	0.997
11-OH-*trans*-12,13-EpOME_2	0.250	1000	0.1195	0.0080	0.999	0.1006	0.0256	0.999
9-OH-*trans*-12,13-EpOME	0.500	1000	0.0167	0.0041	0.999	0.0111	0.0055	0.998
8(*R*),11(*S*)-DiHODE	0.050	200	0.0229	0.0002	0.999	0.0249	0.0003	0.999
erythro-15,16-DiHODE	0.133	106	0.0527	-0.0002	0.999	0.0504	-0.0002	0.999
threo-15,16-DiHODE	N.A.	N.A.	N.A.	N.A.	N.A.	N.A.	N.A.	N.A.
erythro-12,13-DiHODE	0.035	28	0.0162	0.0005	0.999	0.0161	0.0003	0.999
threo-12,13-DiHODE	N.A.	N.A.	N.A.	N.A.	N.A.	N.A.	N.A.	N.A.
erythro-12,13-DiHOME	0.050	25	0.6893	0.0058	0.997	0.6695	0.0092	0.996
threo-12,13-DiHOME	0.025	100	0.1351	0.0062	0.999	0.1298	0.0076	0.999
erythro-9,10-DiHODE	0.017	66	0.0516	0.0001	0.999	0.0551	0.0000	0.999
threo-9,10-DiHODE	0.010	40	0.0404	0.0001	0.999	0.0436	0.0001	0.999
erythro-9,10-DiHOME	0.050	25	0.3354	0.0033	0.996	0.3093	0.0047	0.994

Compound	LLOQ[a] (ng/mL)	ULOQ[b] (ng/mL)	Solvent-matched[c]			Matrix-matched[c]		
			slope	intercept	R^2	slope	intercept	R^2
threo-9,10-DiHOME	0.025	100	0.0596	0.0022	0.999	0.0571	0.0030	0.999
erythro-9,10-DiHODA	0.500	250	0.0064	0.0029	0.996	0.0065	0.0050	0.996
threo-9,10-DiHODA	0.250	250	0.0053	0.0044	0.997	0.0056	0.0059	0.996
13-OH-12-KOME	0.050	200	0.0309	0.0019	0.999	0.0298	0.0020	0.999
13-HOTrE	0.500	250	0.0189	0.0023	0.996	0.0187	0.0026	0.995
13-HOTrE-γ	0.025	100	0.0501	0.0012	0.998	0.0446	0.0013	0.997
13-HODE	0.025	100	0.0612	0.0178	0.999	0.0592	0.0183	0.999
9-HOTrE	0.020	40	0.0386	0.0007	0.997	0.0349	0.0008	0.998
12(*Z*),15(*Z*)/11(*E*),15(*Z*)-10-KODE	0.020	40	0.0586	0.0000	0999	0.0550	0.0002	0.999
9-HODE	0.025	100	0.0477	0.0150	0.999	0.0472	0.0141	0.999
12(*Z*)-10-HOME	0.025	100	0.0727	0.0004	0.999	0.0734	0.0003	0.999
11(*E*)-10-HOME	0.050	200	0.0189	0.0046	0.999	0.0184	0.0046	0.999
13-KOTrE	0.050	200	0.0357	0.0007	0.999	0.0324	0.0013	0.998
9-KOTrE	0.025	100	0.0722	0.0008	0.999	0.0676	0.0005	0.998
12(*Z*),15(*Z*)/11(*E*),15(*Z*)-10-KODE	0.050	100	0.0688	0.0012	0.999	0.0674	0.0007	0.999
13-KODE	0.050	200	0.0780	0.0250	0.999	0.0779	0.0282	0.998
9-KODE	0.050	200	0.0737	0.0101	0.998	0.0731	0.0092	0.998
12(*Z*)-10-KOME	0.100	200	0.0140	0.0010	0.999	0.0137	0.0011	0.998
11(*E*)-10-KOME	0.100	200	0.0154	0.0013	0.999	0.0147	0.0018	0.999
9-oxo-*trans*-12,13-EpOME	0.050	200	0.0255	0.0013	0.998	0.0217	0.0034	0.999
cis-15,16-EpODE	N.A.	N.A.	N.A.	N.A.	N.A.	N.A.	N.A.	N.A.
cis-12,13-EpODE	0.050	100	0.0183	-0.0001	0.998	0.0168	-0.0001	0.998
cis-12,13-EpOME	0.050	200	0.0230	0.0037	0.999	0.0211	0.0037	0.999
trans-12,13-EpOME	0.025	100	0.0270	0.0015	0.999	0.0252	0.0024	0.999
cis-9,10-EpODE	N.A.	N.A.	N.A.	N.A.	N.A.	N.A.	N.A.	N.A.
cis-5,6-EpODE	0.100	200	0.0110	-0.0003	0.999	0.0033	0.0002	0.998
trans-5,6-EpODE	0.050	200	0.0139	0.0001	0.999	0.0083	0.0003	0.998
cis-9,10-EpOME	0.025	100	0.0637	0.0075	0.998	0.0617	0.0075	0.998
trans-9,10-EpOME	N.A.	N.A.	N.A.	N.A.	N.A.	N.A.	N.A.	N.A.
cis-9,10-EpODA	0.250	125	0.0195	0.0217	0.996	0.0217	0.0334	0.995
trans-9,10-EpODA	0.250	125	0.0303	0.0780	0.997	0.0300	0.0372	0.996

[a] LLOQ: lowest limit of quantification. Lowest standard used to build the curve model; accepted if S/N>10 and deviation on the backcalculated standard concentration <20%.

[b] ULOQ: upper limit of quantification. Highest calibrant used to build the curve model.

[c] Slopes and intercepts calculated as average of three curves injected three times.

N.A.=not applicable: the compound is quantified on the curve built on the closest isomer (see Table S8).

VI) Evaluation of the modified SPE procedure

Experimental: Breakthrough determination of polar species

HILIC LC-MS analyses of SPE extracts of surrogate matrix were performed on a Waters Acquity UPLC system coupled to a Xevo-TQ-XS mass spectrometer. Separation was performed on a SeQuant ZIC-HILIC (100 x 2.1 mm, 3.5 µM, 100 Å); Merck Millipore (Billerica, MA, USA), with mobile phase A consisting of Milli-Q water, formic acid 0.1% v/v and mobile phase B consisting of ACN, formic acid 0.1% v/v. The column temperature was set to 25°C, the autosampler temperature was maintained to 8°C, the injection volume was set to 5 µL, and the flow rate was kept at 0.3 mL/min. Gradient elution (illustrated in Table S8) was performed starting from 95% B, which was subsequently linearly increased to 40% at 8.0 min. The column was washed in 25% B for 3 min and re-equilibrated at initial conditions for 5 min. The MS source was operated in negative-ion ESI mode under the following conditions: capillary voltage 2.0 kV, cone voltage 20 V, source offset 30.0 V, source temperature 150°C, desolvation temperature 600°C, cone gas flow 150 L/h, desolvation gas flow 1000 L/h, nebulizer gas pressure 7.0 bar. MS analyses were performed in negative selected ion recording (SIR) mode and phosphates were determined by following the quasimolecular ion at *m/z* 96, corresponding to $[H_2PO_4]^-$, and detected at a retention time of 5.72 min.

Protein concentrations in the SPE extracts of surrogate matrix were measured with a bicinchoninic acid assay (Pierce, Fisher Scientific, Waltham, MA, USA) in a 96 well plate according to the manufacturer's protocol. Since methanol interferes with the protein quantification, the SPE eluates were dried and reconstituted in 20 µL of Milli-Q water. 20 µL of bovine serum albumin (BSA) calibration solutions were prepared in concentrations ranging 25-500 µg/mL. To ensure that drying and reconstitution of the samples was not influencing the correct quantification, a duplicate of the calibrants was dried together with the SPE eluates and reconstituted in 20 µL water, whereas another duplicate was measured directly. 200 µL of BCA solution was added to each well and the sample were subsequently incubated for 30 min at 37°C. Absorbance was measured at 550 nm using an EnSpire 2300 Multilabel Reader (Perkin Elmer, Waltham, MA, USA). To evaluate aspecific contributions from non-protein contaminants which can influence the BCA assay and from proteins different from BSA present in containers and consumables, extraction blanks consisting of PBS and extraction solutions were extracted alongside the samples. The proteins measured in these samples were subtracted to yield the final protein concentration in the SPE eluates.

Results and Discussion: Breakthrough determination of polar species

The scarce solubility of water in supercritical CO_2 limits its use in SFC mobile phases to additive level (up to 5% of water in alcohol can be used)[14]. Additionally, according to manufacturer instructions, AMY-1 columns cannot tolerate water even at trace levels since it would cause permanent alterations to their selectivity. The impossibility to use water in both mobile phase and sample solvent imposes the necessity for a quantitative removal of polar species during SPE, to avoid precipitation in the initial steps of the SFC chromatographic gradient under low MeOH conditions. SPE performance were evaluated regarding phosphates

and proteins, major components of surrogate matrix and extraction solution, which precipitation can be the source of pressure instability and column clogging. The presence of residual proteins and phosphates in SPE eluates was evaluated for the original SPE method[1] and by including 1-4 additional 3 mL water wash steps (Figure S8). While a strong phosphate signal could be detected in the eluate from the original SPE method, a significant decrease could be obtained already with the first additional wash (98.4% phosphates signal decrease), with further decreases until the third wash (Table S9). The final SPE method was designed to include 3 X 3 mL water wash steps after the initial 3 mL MeOH : H_2O 9:1 wash.

Quantification of proteins via BCA assay showed that 0.4-1.2 µg of protein were present in SPE eluates, irrespective of the number of water wash steps (Table S9). The use of the SPE method including 9 mL of additional water wash resulted in the injection of about 15 ng of protein for each sample (SFC method; 35 ng for the LC method, given the higher injection volume).

Table S10. HILIC method for the determination of phosphates in SPE eluates

**Time (min)**	**%A**	**%B**	**Flow rate (mL/min)**
0.0	5.0	95.0	0.30
1.0	5.0	95.0	0.30
8.0	60.0	40.0	0.30
8.1	75.0	25.0	0.30
11.0	75.0	25.0	0.30
11.1	5.0	95.5	0.30
16.0	5.0	95.0	0.30

Column: SeQuant ZIC-HILIC (100 x 2. mm, 3.5µM, 100Å)
Mobile phase A: H_2O, HCOOH 0.1% v/v
Mobile phase B: ACN, HCOOH 0.1% v/v
Injection volume: 5.0µL
Column temperature: 25.0°C

Figure S8. HILIC evaluation of phosphates in eluates with increasing wash volumes

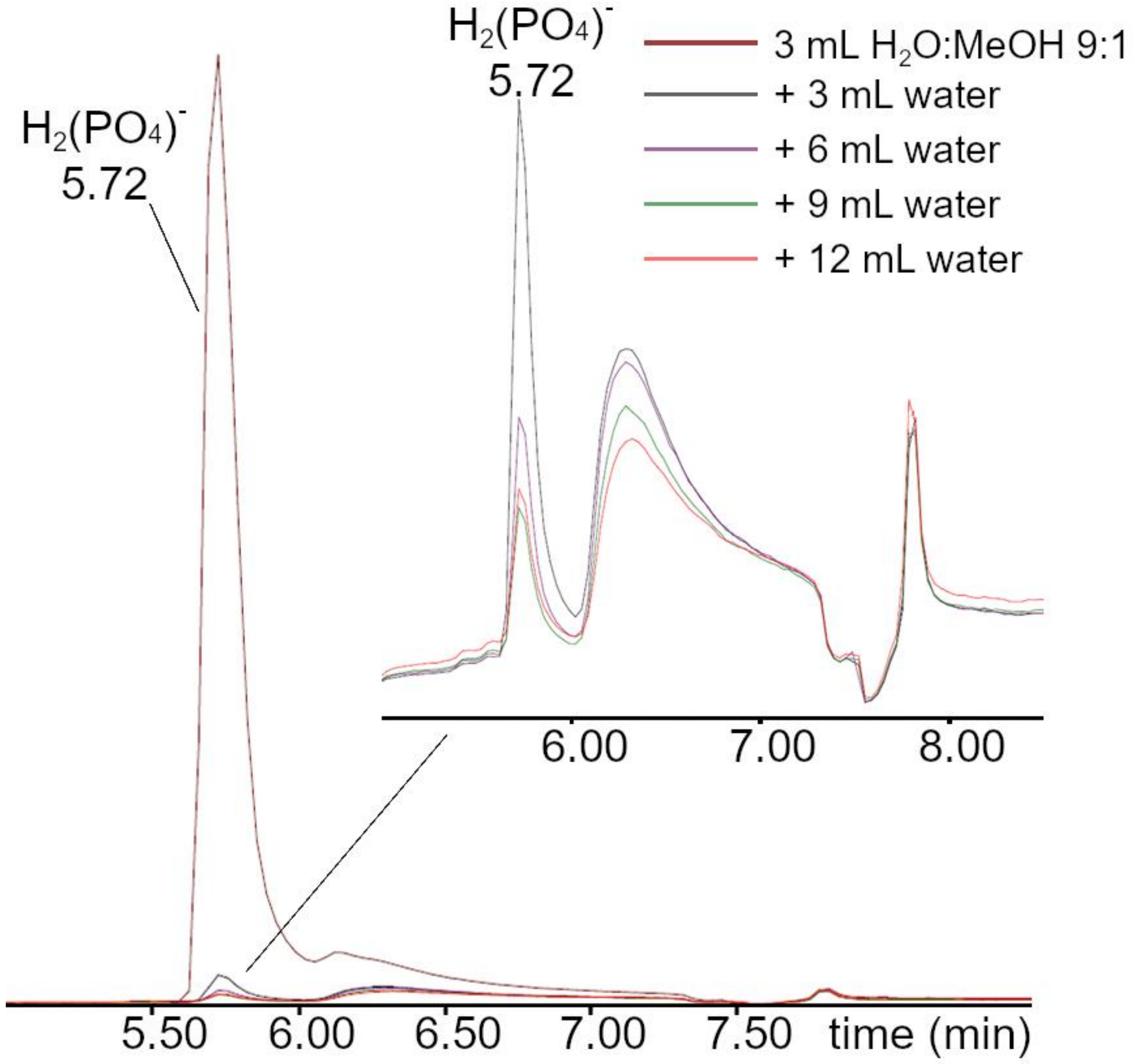

Figure S8. HILIC evaluation of phosphates in eluates with increasing wash volumes. Overlaid chromatogram obtained with the HILIC method described in Table S8 showing the variation of the phosphates peak in different SPE washing conditions. The brown track refers to the phosphates breakthrough by using the original SPE method. Additional wash steps are magnified in the inset.

Table S11. Polar species removal by additional wash steps

**Additional wash step**	**Phosphates in eluate, %**	**Proteins injected SFC (ng on column)**	**Proteins injected LC (ng on column)**
None (3 mL H_2O:MeOH 9:1)	baseline	26.3 ± 3.0	65.8 ± 7.6
3 mL H_2O	-98.4%	13.8 ± 4.6	34.5 ± 11.4
6 mL H_2O	-98.9%	23.8 ± 4.6	59.6 ± 11.4
9 mL H_2O	-99.5%	13.6 ± 1.1	34.1 ± 2.7
12 mL H_2O	-99.5%	29.8 ± 2.9	74.4 ± 7.2

VII) Validation of the analytical methods

Experimental: parameters evaluated during method validation

Sensitivity and linearity. Solvent-matched calibration curves were prepared in three replicates to determine the linear range for the target octadecanoids and the LLOQ. The curves were built by plotting the ratio of the analyte area on the related IS (response) against the theoretical concentration, with a weighting factor of 1/X and a linear fit. The LLOQ for each analyte was determined as the lowest concentration presenting a peak with a signal-to-noise ratio (S/N) ≥5, and relative error (RE) on the back-calculated concentration <30%. For higher levels, the accepted threshold for the RE was lowered to 15%. Matrix-matched calibration curves were prepared by spiking calibration levels and IS solution in surrogate matrix extracts. These curves were used to determine linearity in matrix, to calculate matrix effects, and in the quantification of accuracy and precision in matrix.

Accuracy and precision. Inter- and intra-day accuracy and precision for the whole procedure were determined by spiking quality control (QC) in surrogate matrix at three concentration levels (Low, 3 times LLOQ; Medium, middle point of the linear range; High, 75% of calibration level 9). Six replicates of each QC were extracted each day over a course of three consecutive days to evaluate inter-day precision and accuracy (n=18), while the extracts of the third day were analyzed in triplicate (n=18) to evaluate intra-day precision and accuracy. Accuracy was defined as the closeness between the theoretical concentration of each QC and the average concentration obtained by the repeated measurements, expressed as percentage difference. Precision was defined as the consistency of repeated measurements and was expressed as percentage relative standard deviation (%RSD).

Instrumental accuracy and precision were evaluated in solvent, by preparing at the same concentration levels in pure solvent and directly quantifying them on solvent-matched calibration curves over the course of three consecutive days, in the same number of replicates as described for the matrix validation.

Recovery. The recovery was calculated by spiking QC in surrogate matrix before extraction and comparing the obtained concentration with the concentration measured in the same QC, spiked after extraction (in both cases the IS solution was spiked before SPE). The recovery was calculated at the same three concentration levels used for the determination of accuracy and precision (n=6 per level) and was reported as percentage of the concentration measured in the pre-spiked QC over the concentration measured in the post-spiked QC. IS recovery was calculated both in plasma and in surrogate matrix by comparing the peak area obtained by spiking the IS solution in the biological matrix before and after SPE extraction (n=6).

Matrix effect. Matrix effect was calculated by comparing the slope of the matrix-matched calibration curves (n=3) with the slope of solvent-matched calibration curves (n=3) and was expressed as % RE between the two slopes. IS matrix effect was calculated both in plasma and in surrogate matrix by comparing the peak area of IS spiked in matrix extracts (n=6) with the peak area in solvent (n=6) and expressed as %RE between the average of the two measurements.

Analyte stability. Autosampler stability of the analytes was determined over the course of 96 hours at 8°C, to mimic typical conditions experienced during sample analysis. Stability was

evaluated by the analysis of four SST solution aliquots, spiked with the IS, stored in the autosampler, and quantified at four time points (0, 24, 48, and 96 hours) on freshly prepared calibration curves. The feasibility of leaving the samples for the investigated time range was evaluated by assessing the stability of the measured concentrations. Long-term storage stability at -80°C was not evaluated, because this has been previously reported[15].

Results and discussion: evaluation of instrumental accuracy for deviating species

While most of the investigated octadecanoids showed instrumental accuracy within the acceptable threshold for method validation, 9- and 13-HODE, 11(*E*)-10-KOME, 11(*E*)-10-HOME, and 9-OH-*trans*-12,13-EpOME evidenced extremely high deviation from the theoretical concentration value at both low and medium concentration (300–800%, Table S11). While the last three species are, to the best of our knowledge, included in a broad profiling method for the first time, HODEs are amongst the most studied octadecanoids, and are routinely included in oxylipin analytical platforms[1,16–18]. HODEs data acquired with our general eicosanoid and oxylipin profiling method[1] over the course of one year were used to evaluate the background levels and accuracy of quantification at low concentrations. Background levels ranging 0.28–1.12 ng/mL appeared randomly 50% of the time (data not shown). These levels are comparable with the alterations observed during the method validation and exert a strong effect on the accuracy at low and medium concentrations. Varying background levels may depend on a multitude of factors, including the ubiquitous presence of the analytes in solvents, glassware, and other consumables. It is therefore necessary to perform a careful evaluation of solvent and extraction blanks on each analysis day to avoid reporting of artifacts. Method validation for HODEs has been reported in the literature; however, the investigated linear ranges are generally shifted towards higher concentrations[16–18]. HODEs are relatively high-concentration metabolites, and their reported levels in plasma range 1.5–25 ng/mL[19]. At these levels, comparable to the high-concentration QC used in the LC method validation, HODEs were quantified with an accuracy of 110–113%. The average accuracy levels at low and medium concentrations were affected by the poor performance of the aforementioned compounds. It was therefore concluded that the current LC method was fit for purpose for quantification of the HODEs at the levels expected to be observed in most biological matrices; however, caution should be exerted if levels of these compounds are reported at lower concentrations.

The SFC platform was not significantly affected by background levels of any of the octadecanoids, which is most likely due to the higher detection limit of the technique and the different linear range.

Table S12. Inter- and intra-day accuracy (%) and precision (% CV) for the SFC-MS/MS method: comparison of solvent and matrix validation

Compound	Method (matrix[a])												Instrumental (solvent)											
	Low				Medium				High				Low				Medium				High			
	Intra-day		Inter-day		Intra-day		Inter-day		Intra-day		Inter-day		Intra-day		Inter-day		Intra-day		Inter-day		Intra-day		Inter-day	
	Acc.	Prec.	Acc.	Prec.	Acc.	Prec.	Acc.	Prec.	Acc.	Prec.	Acc.	Prec.	Acc.	Prec.	Acc.	Prec.	Acc.	Prec.	Acc.	Prec.	Acc.	Prec.	Acc.	Prec.
9-KOTrE	78	14	83	18	91	7	100	12	89	5	95	9	95	10	95	11	111	2	110	4	103	2	104	1
13-KOTrE	120	18	129	18	108	7	117	16	105	3	113	11	84	11	86	10	113	3	110	4	105	3	103	3
9-KODE	35	47	51	78	33	7	36	13	32	4	34	9	94	8	93	7	108	2	107	2	103	2	103	2
13-KODE	57	56	73	71	84	6	90	10	78	4	83	9	95	14	95	17	107	2	107	3	102	2	102	2
trans-5,6-EpODE_01	176	23	160	31	118	13	132	20	125	7	137	16	122	11	112	12	97	6	101	6	105	4	107	3
trans-5,6-EpODE_02	228	11	209	11	112	12	124	16	129	7	136	13	100	12	100	9	101	4	100	4	106	3	105	4
cis-5,6-EpODE_01	85	35	101	41	103	17	116	27	114	10	124	23	121	10	116	9	103	5	107	4	116	4	119	5
cis-5,6-EpODE_02	104	28	96	35	98	13	107	18	106	6	115	16	112	10	105	11	102	5	107	6	117	2	118	2
trans-9,10-EpODA_01	79	24	84	21	80	8	82	8	80	5	84	8	101	6	95	13	101	3	100	3	109	1	109	3
trans-9,10-EpODA_02	108	16	108	17	81	9	83	9	81	5	85	7	101	8	96	15	102	3	102	4	107	3	106	4
cis-9(*S*),10(*R*)-EpODA	89	21	89	21	76	8	79	8	77	5	81	8	108	10	103	7	102	2	103	4	109	2	109	4
cis-9(*R*),10(*S*)-EpODA	84	29	89	26	76	9	79	9	78	6	81	8	113	7	113	7	101	2	100	4	109	1	109	4
cis-12,13-EpODE	91	18	98	21	91	8	98	12	95	5	102	11	127	14	119	13	100	3	100	4	110	2	109	4
9(*S*)-HOTrE	92	17	95	15	82	6	90	12	84	3	91	11	106	11	110	7	97	2	98	4	105	1	107	4
13(*S*)-HOTrE	#	#	#	#	#	#	#	#	#	#	#	#	126	11	124	9	99	6	100	7	109	3	112	3
13(*S*)-HOTrE-γ	#	#	#	#	#	#	#	#	#	#	#	#	140	13	130	15	100	6	100	4	108	3	111	4
9(*S*)-HODE	49	104	63	102	85	7	90	10	88	4	94	9	108	20	108	10	105	3	101	5	116	2	111	4
cis-9(*S*),10(*R*)-EpOME	72	26	77	26	82	7	87	9	84	6	90	11	114	14	114	13	99	3	102	4	111	3	109	4
cis-9(*R*),10(*S*)-EpOME	81	25	81	29	79	7	84	10	83	5	89	10	128	17	123	16	100	3	101	4	109	2	110	3
13(*S*)-HODE	149	53	170	50	90	7	97	12	93	5	99	8	116	10	105	10	98	2	99	3	105	3	108	3
trans-12,13-EpOME	#	#	#	#	#	#	#	#	#	#	#	#	114	7	113	6	98	2	100	3	110	3	109	4
cis-12(*R*),13(*S*)-EpOME	100	38	108	36	83	8	88	11	86	7	90	9	107	9	103	7	106	3	101	4	103	2	104	2
cis-12(*S*),13(*R*)-EpOME	105	23	106	20	89	7	93	9	89	4	95	10	102	7	101	6	105	3	102	3	104	3	104	2
12(*Z*)-10-KOME	101	21	106	26	102	7	109	11	100	7	103	7	99	10	98	8	109	3	111	3	102	1	102	2
11(*E*)-10-KOME	88	42	93	44	94	11	100	12	95	6	97	7	99	12	94	10	113	5	110	4	104	1	103	2
12(*Z*),15(*Z*)-10-KODE	79	38	81	37	101	10	112	16	99	5	107	10	101	8	99	7	112	2	108	3	106	2	104	2
11(*E*),15(*Z*)-10-KODE	108	22	113	24	96	7	105	12	91	5	97	10	102	8	102	6	100	3	100	4	104	2	103	2
12(*Z*)-10(*R*)*-HOME	92	17	92	16	87	10	90	10	94	7	95	7	101	8	102	6	106	3	102	4	118	2	114	3
12(*Z*)-10(*S*)*-HOME	84	18	86	17	86	10	89	10	93	7	95	8	107	9	102	8	106	2	103	4	117	1	114	2
11(*E*)-10(*R*)*-HOME	93	22	96	21	94	9	96	9	98	8	99	7	93	13	96	9	105	3	102	4	116	2	111	4
11(*E*)-10(*S*)*-HOME	95	27	99	24	97	10	98	9	101	7	102	7	108	7	107	7	104	3	102	4	103	2	102	2
11(*E*),15(*Z*)-10(*R*)*-HODE	113	64	103	65	82	18	88	17	93	9	96	9	95	18	97	14	107	2	101	6	117	2	112	4
12(*Z*),15(*Z*)-10(*R*)*-HODE	112	12	118	13	84	9	91	12	91	6	95	8	98	10	97	5	107	2	99	4	115	2	109	4
11(*E*),15(*Z*)-10(*S*)*-HODE	96	58	92	49	73	16	79	18	83	7	87	10	88	10	92	7	104	7	100	6	111	5	109	4

Compound	Method (matrix[a])												Instrumental (solvent)											
	Low				Medium				High				Low				Medium				High			
	Intra-day		Inter-day		Intra-day		Inter-day		Intra-day		Inter-day		Intra-day		Inter-day		Intra-day		Inter-day		Intra-day		Inter-day	
	Acc.	Prec.	Acc.	Prec.	Acc.	Prec.	Acc.	Prec.	Acc.	Prec.	Acc.	Prec.	Acc.	Prec.	Acc.	Prec.	Acc.	Prec.	Acc.	Prec.	Acc.	Prec.	Acc.	Prec.
12(*Z*),15(*Z*)-10(*S*)*-HODE	90	12	101	18	84	10	91	12	93	7	98	9	114	8	107	7	105	2	103	4	109	3	106	3
9-oxo-*trans*-12,13-EpOME _01	88	15	90	14	96	5	96	7	94	5	97	9	147	15	133	13	101	5	104	5	110	3	111	3
9-oxo-*trans*-12,13-EpOME _02	84	18	85	14	97	7	97	9	93	5	97	10	135	13	118	15	100	4	102	4	108	3	112	4
11-OH-*trans*-9,10-EpOME_01	92	22	99	25	83	15	87	15	92	11	93	12	109	8	106	6	100	3	97	4	105	3	105	4
11-OH-*trans*-9,10-EpOME_02	77	15	86	24	82	9	89	12	83	5	89	10	113	14	105	12	96	6	98	6	103	3	104	4
11-OH-*trans*-9,10-EpOME_03	107	16	113	19	82	17	87	16	90	10	92	10	98	7	95	7	99	4	97	5	103	2	103	2
11-OH-*trans*-9,10-EpOME_04	104	14	111	19	80	10	86	13	85	8	89	10	109	12	108	10	97	5	101	6	105	2	106	4
13-OH-*trans*-9,10-EpOME_01	124	15	127	14	91	9	96	10	91	6	97	11	104	7	106	7	105	3	102	5	114	2	110	5
13-OH-*trans*-9,10-EpOME_02	110	12	114	11	95	10	100	11	93	6	99	10	105	7	105	6	105	3	102	5	112	2	110	4
13-OH-*trans*-9,10-EpOME_03	114	11	116	19	92	7	98	9	93	6	98	9	102	9	100	7	105	3	103	5	114	2	110	5
9-OH-*trans*-12,13-EpOME_01	125	20	125	18	104	12	111	13	112	9	116	10	101	12	105	9	106	4	103	5	117	2	111	5
9-OH-*trans*-12,13-EpOME_02	108	21	115	18	107	11	112	11	114	7	118	9	104	11	105	9	104	4	101	6	114	3	110	5
9-OH-*trans*-12,13-EpOME_03	104	22	106	18	110	14	115	13	122	10	123	10	96	9	97	8	104	3	102	5	111	2	108	4
11-OH-*trans*-12,13-EpOME_01	95	10	103	15	101	8	107	10	103	6	108	10	106	10	105	10	101	3	97	4	111	3	106	4
11-OH-*trans*-12,13-EpOME_02	111	18	116	18	101	10	107	11	106	7	110	9	98	6	97	6	105	3	103	6	110	3	106	5
11-OH-*trans*-12,13-EpOME_03	114	13	124	14	103	8	109	11	104	6	110	10	106	10	108	7	103	4	101	6	112	2	108	5
11-OH-*trans*-12,13-EpOME_04	105	12	109	12	105	6	111	10	105	6	111	10	117	9	107	13	95	5	98	5	101	3	103	4
13-OH-12-KOME	101	12	105	14	74	4	74	8	68	3	73	10	99	15	100	11	109	2	101	6	121	2	112	6
erythro-9,10-DiHODE	83	13	87	13	85	6	92	11	83	5	92	13	92	10	98	11	110	3	104	6	119	4	111	6
erythro-12,13-DiHODE	-	-	-	-	93	7	99	11	91	6	98	9	-	-	-	-	98	5	99	4	104	4	107	5
erythro-15,16-DiHODE	121	14	117	21	91	7	96	9	93	7	97	8	146	23	120	14	95	6	98	6	100	4	105	3
threo-9(*R*),10(*R*)-DiHOME	78	21	83	18	78	7	83	10	80	4	85	9	106	12	102	9	105	3	103	3	116	2	112	3
threo-9(*S*),10(*S*)-DiHOME	83	18	91	18	84	7	88	9	82	5	87	9	108	15	107	11	109	3	104	4	112	2	109	4
*threo*_1-+*threo*_2-+*erythro*_1-12,13-DiHOME	89	9	90	10	83	8	88	10	86	5	90	7	108	9	100	11	98	3	99	3	103	2	106	3
threo-9(*R*),10(*R*)-DiHODA*	-	-	-	-	82	13	85	11	79	6	81	6	111	29	111	10	100	3	98	4	110	2	109	3
*threo*_2-+*erythro*_1-9,10-DiHODA	-	-	-	-	82	9	84	8	81	5	82	6	104	8	103	9	101	2	102	4	110	2	109	4
erythro-9,10-DiHODA_2	-	-	-	-	85	8	86	9	85	7	87	7	122	14	117	7	100	4	100	4	103	2	104	2
9(*S*),10(*S*),13(*S*)-TriHODE	93	24	93	22	94	8	104	17	92	5	106	20	99	15	100	11	101	3	98	4	110	2	108	3
9(*S*),10(*S*),11(*R*)-TriHODE	#	#	#	#	#	#	#	#	#	#	#	#	-	-	-	-	98	7	97	6	113	2	110	2
9(*S*),12(*S*),13(*S*)-TriHODE	76	36	77	31	81	7	89	15	78	4	90	19	82	18	95	10	96	2	97	4	105	2	107	4
9(*S*),10(*S*),13(*S*)-TriHOME	129	34	124	31	86	9	93	13	81	5	92	17	94	20	99	13	108	2	103	6	119	1	114	5
9(*S*),10(*S*),11(*R*)-TriHOME	92	28	95	27	83	9	87	13	75	5	85	18	100	14	99	10	96	3	98	2	105	2	106	2
11(*R*),12(*S*),13(*S*)-TriHOME	70	23	69	24	76	8	79	12	70	6	79	19	101	10	98	10	107	3	101	5	120	2	113	5
9(*S*),12(*S*),13(*S*)-TriHOME	200	36	192	26	60	9	64	11	57	5	60	7	84	17	90	15	98	1	99	4	107	2	108	2

-Not detected. #Could not be determined due to technical issues. [a]Includes SPE extraction and matrix effect. * Order of elution and identity not confirmed with an enantiopure standard but inferred by comparison with similar compounds under the same conditions.

Table S13. Inter- and intra-day accuracy (%) and precision (% CV) for the LC-MS/MS method: comparison of solvent and matrix validation

Compound	Method (matrix[a])												Instrumental (solvent)											
	Low				Medium				High				Low				Medium				High			
	Intra-day		Inter-day		Intra-day		Inter-day		Intra-day		Inter-day		Intra-day		Inter-day		Intra-day		Inter-day		Intra-day		Inter-day	
	Acc.	Prec.	Acc.	Prec.	Acc.	Prec.	Acc.	Prec.	Acc.	Prec.	Acc.	Prec.	Acc.	Prec.	Acc.	Prec.	Acc.	Prec.	Acc.	Prec.	Acc.	Prec.	Acc.	Prec.
9-KOTrE	84	7	82	8	107	2	109	4	112	3	113	3	100	8	100	7	108	2	104	5	108	1	109	1
13-KOTrE	65	6	61	8	66	4	64	7	66	5	64	8	107	6	104	7	110	3	107	4	106	2	108	2
9-KODE	230	9	268	21	115	2	120	9	96	3	96	4	104	7	98	11	110	3	106	4	111	1	111	1
13-KODE	217	9	249	15	119	2	121	5	103	3	101	4	112	13	103	10	112	3	106	5	112	2	111	3
cis-5,6-EpODE	29	11	50	34	28	45	45	53	25	44	39	56	130	16	134	13	103	9	110	12	104	8	102	6
trans-5,6-EpODE	44	21	56	21	57	19	68	24	55	19	65	25	111	7	111	7	106	4	109	5	106	6	105	4
cis-9,10-EpODA	285	6	314	10	128	4	127	4	97	3	94	5	94	6	95	6	106	2	105	3	96	1	96	1
trans-9,10-EpODA	229	10	348	43	108	5	115	15	80	4	79	5	98	5	99	5	90	4	97	7	81	1	84	4
cis-12,13-EpODE	107	7	101	10	120	3	119	3	125	4	124	5	113	9	108	9	102	3	97	4	101	1	100	1
cis-9,10-EpOME	188	10	204	14	133	2	134	3	125	3	124	4	105	11	102	13	110	2	109	3	107	1	111	3
cis-12,13-EpOME	126	8	126	8	98	2	98	2	96	3	94	4	110	6	108	6	107	3	103	3	108	1	106	2
trans-12,13-EpOME	261	12	293	16	165	3	166	4	146	3	144	5	103	9	101	10	103	3	101	3	104	1	103	1
9-HOTrE	156	10	157	10	122	2	124	3	116	4	116	4	104	11	101	10	116	2	113	5	113	1	115	2
13-HOTrE	102	6	99	8	130	2	131	3	125	4	124	4	108	2	110	3	115	2	114	2	101	1	104	3
13-HOTrE-γ	111	9	114	10	128	2	130	4	125	5	125	5	116	7	109	13	111	2	109	3	101	2	107	6
9-HODE	925	12	1119	21	285	4	294	9	128	2	127	3	655	15	360	68	181	4	150	20	110	1	111	2
13-HODE	836	12	1075	24	285	4	296	8	123	2	121	4	678	15	383	64	182	3	148	22	113	1	111	3
12(*Z*)-10-KOME	75	8	77	7	74	7	77	8	77	3	78	4	104	7	101	7	109	4	106	4	110	1	112	1
11(*E*)-10-KOME	79	7	110	26	93	5	98	10	85	3	85	4	328	8	201	48	132	5	117	12	113	2	113	2
12(*Z*),15(*Z*)/11(*E*),15(*Z*)-10-KODE	67	7	68	9	73	3	76	6	76	4	77	5	99	7	101	8	109	3	105	5	109	1	109	1
12(*Z*)-10-HOME	-	-	-	-	65	5	73	22	71	5	71	6	114	3	112	2	108	2	106	3	107	1	108	2
11(*E*)-10-HOME	-	-	-	-	83	13	85	14	49	5	48	6	794	2	471	49	210	2	171	24	116	1	110	4
12(*Z*),15(*Z*)/11(*E*),15(*Z*)-10-HODE	74	10	71	11	80	8	80	6	84	3	85	5	107	5	106	6	105	2	103	2	108	1	109	2
9-oxo-*trans*-12,13-EpOME	274	6	353	27	137	4	136	8	117	3	110	9	106	6	105	6	114	2	111	4	116	1	114	2
11-OH-*trans*-9,10-EpOME_1	77	6	80	8	72	8	72	7	74	4	73	5	115	5	113	8	104	3	107	3	97	3	99	3
11-OH-*trans*-9,10-EpOME_2	66	7	69	8	68	8	67	7	71	3	70	5	111	4	107	5	105	3	107	3	102	4	100	3
13-OH-*trans*-9,10-EpOME	83	13	96	23	109	3	109	4	110	3	108	5	98	5	97	8	110	4	106	6	101	2	102	2
9-OH-*trans*-12,13-EpOME	561	9	1093	46	302	6	313	12	113	4	111	5	771	11	344	91	493	5	328	45	97	3	96	3
11-OH-*trans*-12,13-EpOME_1	70	6	69	6	91	6	92	5	86	4	85	4	95	5	100	6	117	4	114	4	107	3	107	3
11-OH-*trans*-12,13-EpOME_2	75	7	74	7	90	5	90	5	88	3	86	5	99	2	100	5	109	4	109	5	104	4	105	3
13-OH-12-KOME	83	6	109	22	103	5	97	9	96	4	91	10	165	6	143	11	114	2	112	4	109	1	110	1
8(*R*)-11(*S*)-DiHODE	132	7	127	9	180	7	172	9	180	6	172	9	102	3	103	4	106	1	103	3	107	1	108	1
erythro-9,10-DiHODE	132	5	131	7	174	7	175	6	176	5	176	5	111	5	113	4	112	1	110	3	113	1	114	1
threo-9,10-DiHODE	68	9	68	13	75	6	76	5	74	5	74	5	111	5	101	8	101	3	101	2	105	2	106	2

Compound	Method (matrix[a])												Instrumental (solvent)											
	Low				Medium				High				Low				Medium				High			
	Intra-day		Inter-day		Intra-day		Inter-day		Intra-day		Inter-day		Intra-day		Inter-day		Intra-day		Inter-day		Intra-day		Inter-day	
	Acc.	Prec.	Acc.	Prec.	Acc.	Prec.	Acc.	Prec.	Acc.	Prec.	Acc.	Prec.	Acc.	Prec.	Acc.	Prec.	Acc.	Prec.	Acc.	Prec.	Acc.	Prec.	Acc.	Prec.
erythro-12,13-DiHODE	-	-	-	-	97	6	95	6	82	5	83	6	-	-	-	-	94	6	93	7	101	2	101	2
erythro-15,16-DiHODE	75	6	72	7	82	5	82	4	84	6	85	6	128	8	129	7	108	2	104	3	109	2	110	1
erythro-9,10-DiHOME	101	4	97	8	147	3	145	3	141	4	140	4	108	2	107	3	111	1	112	2	98	1	103	3
threo-9,10-DiHOME	163	13	155	15	126	3	124	4	121	3	119	4	99	7	98	7	114	1	110	4	112	1	111	1
erythro-12,13-DiHOME	102	3	97	8	143	1	142	1	138	2	137	3	107	2	107	3	114	1	111	3	104	2	105	2
threo-12,13-DiHOME	151	11	144	13	119	2	118	3	122	2	120	4	97	6	99	6	105	3	102	3	107	2	106	2
erythro-9,10-DiHODA	150	9	186	19	87	5	87	5	76	3	74	5	128	7	115	11	129	2	122	7	104	1	105	1
threo-9,10-DiHODA	170	12	220	23	90	5	90	6	74	3	73	5	103	6	101	6	129	2	122	7	104	1	105	1
9,10,13-TriHODE	347	11	358	12	251	8	250	9	209	10	207	10	100	7	97	7	106	4	103	5	107	2	105	3
9,10,11-TriHODE	205	8	216	10	225	8	224	10	208	9	206	9	136	10	128	10	106	4	102	6	105	1	105	3
9,12,13-TriHODE	201	12	212	12	185	6	182	8	171	9	168	9	109	5	112	6	103	3	100	4	104	2	103	2
9,10,13-TriHOME	615	6	828	24	278	7	286	10	196	8	191	9	118	9	116	13	102	4	101	5	99	2	99	2
9,10,11-TriHOME	158	5	154	8	222	8	216	9	212	8	205	9	110	5	109	6	100	2	98	4	102	1	102	2
11,12,13-TriHOME	221	5	219	8	268	8	259	9	257	8	248	9	109	4	109	4	100	2	98	3	102	2	102	2
9,12,13-TriHOME	680	8	883	23	211	3	224	12	117	4	115	5	131	11	121	17	102	4	105	4	99	4	99	3
8-F_{1t}-$PhytoP_{DGLA}$	58	8	57	10	74	4	74	4	81	4	80	4	99	5	99	6	114	3	110	6	109	2	109	2
15-F_{1t}-$PhytoP_{DGLA}$	74	7	74	6	96	6	96	5	99	11	99	9	103	5	102	7	107	3	105	3	106	1	106	3
6-F_{2t}-$PhytoP_{SDA}$	58	12	62	13	57	8	59	9	58	5	59	7	95	7	94	11	98	4	102	6	101	2	106	4
16-F_{2t}-$PhytoP_{SDA}$	-	-	-	-	90	12	93	14	85	7	86	8	-	-	-	-	93	9	93	9	110	3	113	3
6-F_{1t}-$PhytoP_{GLA}$	61	15	62	12	68	5	68	5	71	3	71	4	92	11	93	23	106	4	106	6	107	2	110	3
13-F_{1t}-$PhytoP_{GLA}$	71	8	70	10	93	5	93	5	97	3	96	3	100	4	99	6	104	2	105	3	103	2	104	1
9-F_{1t}-$PhytoP_{ALA}$	121	6	130	9	74	5	74	5	64	5	64	5	86	7	87	16	113	5	109	7	109	2	112	3
16-F_{1t}-$PhytoP_{ALA}$	120	11	125	15	75	8	76	9	73	4	72	5	102	8	104	13	112	4	109	6	109	1	114	3
9-L_1-$PhytoP_{ALA}$	498	6	488	6	167	2	165	3	116	5	115	5	101	6	106	9	110	4	109	4	108	2	109	2
16-B_1-$PhytoP_{ALA}$	282	8	277	7	104	3	105	4	81	5	80	5	101	8	99	12	113	3	110	4	108	2	109	2
ent-9-D_{1t}-$PhytoP_{ALA}$	69	9	70	9	83	6	82	8	94	3	93	4	95	6	95	7	112	5	107	6	102	2	103	2
ent-9-12-*epi*-ST-Δ^{10}-13-PhytoF	77	9	77	8	89	4	89	4	91	6	90	8	94	9	100	8	108	3	108	3	109	2	108	1
ent-16-13-*epi*-ST-Δ^{14}-9-PhytoF	84	10	88	10	62	5	62	6	52	6	52	8	99	7	103	6	103	2	104	4	103	1	105	2

-Not detected

[a]Includes SPE extraction and matrix effect

Table S14. Recovery and matrix effect in plasma and surrogate matrix (SFC-MS/MS method)

**Compound**	**Recovery (%)**[a]				**Matrix effect**[b]	
	**Low conc.**	**Medium conc.**	**High conc.**	**ISTD Plasma**	**Plasma**	**Surrogate matrix**
13-KODE-d_3	N.A.	N.A.	N.A.	91±6%	39%	47%
9(*S*)-HODE-d_4	N.A.	N.A.	N.A.	94±6%	33%	46%
13(S)-HODE-d_4	N.A.	N.A.	N.A.	81±6%	32%	44%
cis-12,13-EpOME-d_4	N.A.	N.A.	N.A.	54±6%	38%	49%
13-OH-9,10-EpOME-d_5	N.A.	N.A.	N.A.	92±5%	31%	42%
threo-9,10-DiHOME-d_4_1	N.A.	N.A.	N.A.	93±9%	33%	47%
threo-9,10-DiHOME-d_4_2	N.A.	N.A.	N.A.	88±12%	74%	44%
threo-12,13-DiHOME-d_4_1	N.A.	N.A.	N.A.	94±6%	34%	44%
threo-12,13-DiHOME-d_4_2	N.A.	N.A.	N.A.	96±9%	34%	42%
9(*S*),12(*S*),13(*S*)-($^{13}C_3$)-TriHOME	N.A.	N.A.	N.A.	96±8%	43%	45%
9-KOTrE	73±15%	87±6%	86±9%	N.A.	N.A.	3%
13-KOTrE	56±11%	66±13%	70±13%	N.A.	N.A.	-2%
9-KODE	120±47%	92±7%	92±10%	N.A.	N.A.	1%
13-KODE	94±34%	89±5%	89±9%	N.A.	N.A.	1%
trans-5,6-EpODE_01	82±26%	75±10%	71±13%	N.A.	N.A.	-6%
trans-5,6-EpODE_02	56±14%	60±8%	55±7%	N.A.	N.A.	-1%
cis-5,6-EpODE_01	64±8%	56±12%	51±10%	N.A.	N.A.	-20%
cis-5,6-EpODE_02	43±16%	74±10%	70±14%	N.A.	N.A.	-7%
trans-9,10-EpODA_01	61±11%	67±8%	63±8%	N.A.	N.A.	9%
trans-9,10-EpODA_02	62±8%	62±8%	56±6%	N.A.	N.A.	13%
cis-9(*S*),10(*R*)-EpODA	69±11%	66±9%	62±7%	N.A.	N.A.	7%
cis-9(*R*),10(*S*)-EpODA	58±7%	55±8%	51±6%	N.A.	N.A.	8%
cis-12,13-EpODE	69±15%	77±6%	71±10%	N.A.	N.A.	7%
9(*S*)-HOTrE	81±12%	88±8%	78±12%	N.A.	N.A.	7%
13(*S*)-HOTrE-γ	81±5%	90±5%	79±13%	N.A.	N.A.	8%
9(*S*)-HODE	116±27%	89±5%	77±10%	N.A.	N.A.	11%
cis-9(*S*),10(*R*)-EpOME	65±8%	80±6%	75±10%	N.A.	N.A.	6%
cis-9(*R*),10(*S*)-EpOME	60±11%	64±9%	60±7%	N.A.	N.A.	9%
13(*S*)-HODE	102±23%	90±7%	78±11%	N.A.	N.A.	8%
cis-12(*R*),13(*S*)-EpOME	48±11%	54±9%	51±5%	N.A.	N.A.	5%
cis-12(*S*),13(*R*)-EpOME	76±14%	78±7%	74±9%	N.A.	N.A.	15%
12(*Z*)-10-KOME	47±16%	70±9%	70±5%	N.A.	N.A.	4%
11(*E*)-10-KOME	56±33%	72±3%	71±5%	N.A.	N.A.	3%
12(*Z*),15(*Z*)-10-KODE	54±13%	81±3%	80±9%	N.A.	N.A.	5%
11(*E*),15(*Z*)-10-KODE	66±13%	88±6%	87±10%	N.A.	N.A.	-3%
12(*Z*)-10(*R*)*-HOME	44±7%	51±2%	46±4%	N.A.	N.A.	16%
12(*Z*)-10(*S*)*-HOME	43±5%	44±4%	41±3%	N.A.	N.A.	15%
11(*E*)-10(*R*)*-HOME	37±5%	38±4%	34±3%	N.A.	N.A.	13%
11(*E*)-10(*S*)*-HOME	36±7%	34±4%	30±2%	N.A.	N.A.	14%
11(*E*),15(*Z*)-10(*R*)*-HODE	40±9%	48±2%	43±4%	N.A.	N.A.	10%
12(*Z*),15(*Z*)-10(*R*)*-HODE	64±5%	66±4%	59±5%	N.A.	N.A.	16%
11(*E*),15(*Z*)-10(*S*)*-HODE	66±19%	66±4%	59±7%	N.A.	N.A.	16%

Compound	Recovery (%)[a]				Matrix effect[b]	
	Low conc.	Medium conc.	High conc.	ISTD Plasma	Plasma	Surrogate matrix
12(*Z*),15(*Z*)-10(*S*)*-HODE	73±6%	66±3%	57±5%	N.A.	N.A.	17%
9-oxo-*trans*-12,13-EpOME _01	78±9%	80±8%	77±12%	N.A.	N.A.	7%
9-oxo-*trans*-12,13-EpOME _02	77±9%	83±10%	81±14%	N.A.	N.A.	11%
11-OH-*trans*-9,10-EpOME_01	62±7%	53±6%	49±4%	N.A.	N.A.	5%
11-OH-*trans*-9,10-EpOME_02	108±18%	96±9%	89±4%	N.A.	N.A.	5%
11-OH-*trans*-9,10-EpOME_03	73±6%	74±5%	66±4%	N.A.	N.A.	10%
11-OH-*trans*-9,10-EpOME_04	87±17%	84±7%	77±3%	N.A.	N.A.	4%
13-OH-*trans*-9,10-EpOME_01	82±11%	93±4%	87±4%	N.A.	N.A.	7%
13-OH-*trans*-9,10-EpOME_02	85±13%	93±4%	88±3%	N.A.	N.A.	9%
13-OH-*trans*-9,10-EpOME_03	89±11%	94±4%	87±3%	N.A.	N.A.	9%
9-OH-*trans*-12,13-EpOME_01	72±12%	74±5%	69±3%	N.A.	N.A.	8%
9-OH-*trans*-12,13-EpOME_02	81±13%	78±4%	72±3%	N.A.	N.A.	9%
9-OH-*trans*-12,13-EpOME_03	48±7%	55±4%	51±3%	N.A.	N.A.	8%
11-OH-*trans*-12,13-EpOME_01	88±9%	94±8%	87±3%	N.A.	N.A.	12%
11-OH-*trans*-12,13-EpOME_02	88±12%	86±7%	81±3%	N.A.	N.A.	15%
11-OH-*trans*-12,13-EpOME_03	92±10%	95±9%	88±3%	N.A.	N.A.	5%
11-OH-*trans*-12,13-EpOME_04	90±9%	98±10%	89±4%	N.A.	N.A.	0%
13-OH-12-KOME	78±4%	79±8%	79±15%	N.A.	N.A.	16%
erythro-9,10-DiHODE	72±10%	87±8%	81±10%	N.A.	N.A.	14%
erythro-12,13-DiHODE	98±20%	87±8%	80±7%	N.A.	N.A.	10%
erythro-15,16-DiHODE	74±21%	86±4%	77±5%	N.A.	N.A.	8%
threo-9(*R*),10(*R*)-DiHOME	66±10%	74±5%	69±6%	N.A.	N.A.	12%
threo-9(*S*),10(*S*)-DiHOME	68±10%	74±7%	74±8%	N.A.	N.A.	5%
*threo*_1-+*threo*_2-+*erythro*_1-12,13-DiHOME	84±5%	94±4%	82±6%	N.A.	N.A.	8%
threo-9(*R*),10(*R*)-DiHODA*	32±5%	39±4%	35±3%	N.A.	N.A.	14%
*threo*_2-+*erythro*_1-9,10-DiHODA	38±6%	44±4%	41±3%	N.A.	N.A.	12%
erythro-9,10-DiHODA_2	62±10%	52±6%	51±5%	N.A.	N.A.	3%
9(*S*),10(*S*),13(*S*)-TriHODE	76±16%	95±14%	99±11%	N.A.	N.A.	6%
9(*S*),10(*S*),11(*R*)-TriHODE	82±15%	94±13%	97±12%	N.A.	N.A.	10%
9(*S*),12(*S*),13(*S*)-TriHODE	74±17%	92±13%	96±10%	N.A.	N.A.	5%
9(*S*),10(*S*),13(*R*)-TriHOME	89±14%	103±14%	107±13%	N.A.	N.A.	N.A.
9(*R*),10(*S*),13(*R*)-TriHOME	82±17%	103±15%	106±12%	N.A.	N.A.	N.A.
9(*S*),10(*S*),13(*S*)-TriHOME	79±29%	102±13%	107±12%	N.A.	N.A.	11%
9(*R*),10(*S*),13(*S*)-TriHOME	90±24%	109±14%	112±13%	N.A.	N.A.	N.A.
9(*S*),10(*R*),13(*R*)-TriHOME	95±22%	112±17%	114±16%	N.A.	N.A.	N.A.
9(*S*),10(*R*),13(*S*)-TriHOME	101±29%	101±15%	110±14%	N.A.	N.A.	N.A.
9(*R*),10(*R*),13(*R*)-TriHOME	86±7%	106±16%	110±13%	N.A.	N.A.	N.A.
9(*R*),10(*R*),13(*S*)-TriHOME	119±39%	102±13%	108±13%	N.A.	N.A.	N.A.
9(*S*),10(*S*),11(*R*)-TriHOME	90±18%	103±15%	108±13%	N.A.	N.A.	6%
11(*R*),12(*S*),13(*S*)-TriHOME	84±17%	100±14%	106±14%	N.A.	N.A.	14%
9(*R*),12(*S*),13(*S*)-TriHOME	82±21%	98±12%	102±11%	N.A.	N.A.	N.A.
9(*S*),12(*S*),13(*R*)-TriHOME	92±17%	107±16%	114±13%	N.A.	N.A.	N.A.
9,12,13-TriHOME (*S*,*R*,*S*+*R*,*S*,*R*)	87±14%	106±14%	113±14%	N.A.	N.A.	N.A.

Compound	Recovery (%)[a]				Matrix effect[b]	
	**Low conc.**	**Medium conc.**	**High conc.**	**ISTD Plasma**	**Plasma**	**Surrogate matrix**
9(*S*),12(*S*),13(*S*)-TriHOME	53±13%	47±6%	47±3%	N.A.	N.A.	11%
9(*S*),12(*R*),13(*R*)-TriHOME	72±24%	107±19%	111±15%	N.A.	N.A.	N.A.
9,12,13-TriHOME (*R*,*R*,*S*+*R*,*R*,*R*)	90±14%	104±15%	110±11%	N.A.	N.A.	N.A.
Average[c]	74±14%	80±8%	77±8%	N.A.	N.A.	8%
Range (Minimum - Maximum)[c]	32-120%	34-112%	30-114%	N.A.	N.A.	-20-54%

* Order of elution and identity not confirmed with an enantiopure standard but inferred by comparison with similar compounds under the same conditions.

[a] Errors are calculated as %CV on 6 analytical replicates.

[b] Matrix effect for ISTD determined as the percentage relative error between the signal for internal standards measured in solvent and the signal for the same compounds post-spiked in matrix extracts. Matrix effect for octadecanoids calculated as % relative error of the slopes of the solvent-matched calibration curve and the post-spiked calibration curve built on the same linear range.

[c] Average values and ranges do not include internal standards (ISTDs).

N.A.=not applicable.

Table S15. Recovery and matrix effect in plasma and surrogate matrix (LC-MS/MS method)

Compound	Recovery (%)[a]				Matrix effect[b]	
	Low conc.	Medium conc.	High conc.	ISTD plasma	Plasma	Surrogate matrix
9-KODE-d_3	N.A.	N.A.	N.A.	84±4%	-51%	-6%
9(*S*)-HODE-d_4	N.A.	N.A.	N.A.	83±4%	-45%	-7%
13(*S*)-HODE-d_4	N.A.	N.A.	N.A.	73±3%	-52%	-9%
cis-9,10-EpOME-d_4	N.A.	N.A.	N.A.	66±3%	-47%	-7%
cis-12,13-EpOME-d_4	N.A.	N.A.	N.A.	53±2%	-40%	-5%
threo-9,10-DiHOME-d_4	N.A.	N.A.	N.A.	83±3%	-28%	-13%
threo-12,13-DiHOME-d_4	N.A.	N.A.	N.A.	86±3%	-14%	-5%
9(*S*),12(*S*),13(*S*)-($^{13}C_3$)-TriHOME	N.A.	N.A.	N.A.	88±3%	-21%	-4%
13-OH-9,10-EpOME-d_5	N.A.	N.A.	N.A.	67±3%	-33%	-16%
16-F_{1t}-PhytoP-C_{19}	N.A.	N.A.	N.A.	88±3%	-22%	-13%
9-KOTrE	103±24%	74±4%	75±1%	N.A.	N.A.	-6%
13-KOTrE	79±20%	70±3%	68±1%	N.A.	N.A.	-9%
9-KODE	91±21%	81±13%	82±1%	N.A.	N.A.	-1%
13-KODE	91±27%	80±4%	78±1%	N.A.	N.A.	0%
cis-5,6-EpODE	N.D.	93±7%	71±12%	N.A.	N.A.	-70%
trans-5,6-EpODE	67±13%	63±13%	64±8%	N.A.	N.A.	-40%
cis-9,10-EpODA	73±11%	45±8%	35±3%	N.A.	N.A.	-5%
trans-9,10-EpODA	106±38%	36±7%	34±3%	N.A.	N.A.	-1%
cis-12,13-EpODE	73±6%	63±5%	55±9%	N.A.	N.A.	-8%
cis-9,10-EpOME	104±28%	71±7%	59±10%	N.A.	N.A.	-3%
cis-12,13-EpOME	79±13%	62±6%	54±14%	N.A.	N.A.	-8%
trans-12,13-EpOME	89±25%	84±14%	63±7%	N.A.	N.A.	-7%
9-HOTrE	79±17%	75±5%	75±4%	N.A.	N.A.	-9%
13-HOTrE	80±17%	80±2%	87±2%	N.A.	N.A.	-1%
13-HOTrE-γ	N.D.	82±2%	84±6%	N.A.	N.A.	-11%
9-HODE	66±3%	66±1%	66±6%	N.A.	N.A.	-1%
13-HODE	71±10%	78±5%	73±3%	N.A.	N.A.	-3%
12(*Z*)-10-KOME	N.D.	58±6%	52±19%	N.A.	N.A.	-2%
11(*E*)-10-KOME	61±6%	63±8%	58±14%	N.A.	N.A.	-5%
12(*Z*),15(*Z*)/11(*E*),15(*Z*)-10-KODE	N.D.	83±3%	74±3%	N.A.	N.A.	-2%
12(*Z*)-10-HOME	60±8%	38±2%	32±2%	N.A.	N.A.	1%
11(*E*)-10-HOME	13±1%	24±1%	26±1%	N.A.	N.A.	-2%
12(*Z*),15(*Z*)/11(*E*),15(*Z*)-10-ODE	N.D.	51±3%	46±15%	N.A.	N.A.	-6%
9-oxo-*trans*-12,13-EpOME	77±11%	70±3%	60±9%	N.A.	N.A.	-15%
11-OH-*trans*-9,10-EpOME_1	94±16%	67±4%	77±2%	N.A.	N.A.	-21%
11-OH-*trans*-9,10-EpOME_2	100±19%	65±6%	67±5%	N.A.	N.A.	-18%
13-OH-*trans*-9,10-EpOME	100±15%	77±1%	89±2%	N.A.	N.A.	-3%
9-OH-*trans*-12,13-EpOME	N.D.	83±2%	74±2%	N.A.	N.A.	-33%
11-OH-*trans*-12,13-EpOME_1	69±6%	79±4%	90±1%	N.A.	N.A.	-10%
11-OH-*trans*-12,13-EpOME_2	99±6%	78±5%	89±1%	N.A.	N.A.	-16%
13-OH-12-KOME	72±16%	87±1%	87±3%	N.A.	N.A.	-4%
8(*R*)-11(*S*)-DiHODE	88±8%	86±2%	88±6%	N.A.	N.A.	9%

Compound	Recovery (%)[a]				Matrix effect[b]	
	Low conc.	Medium conc.	High conc.	ISTD plasma	Plasma	Surrogate matrix
erythro-9,10-DiHODE	79±4%	73±3%	71±3%	N.A.	N.A.	7%
threo-9,10-DiHODE	85±7%	74±7%	68±5%	N.A.	N.A.	8%
erythro-12,13-DiHODE	N.D.	85±4%	66±1%	N.A.	N.A.	-1%
erythro-15,16-DiHODE	91±7%	83±20%	73±6%	N.A.	N.A.	-4%
erythro-9,10-DiHOME	60±3%	69±6%	82±9%	N.A.	N.A.	-8%
threo-9,10-DiHOME	68±24%	65±6%	64±12%	N.A.	N.A.	-4%
erythro-12,13-DiHOME	73±8%	80±3%	70±6%	N.A.	N.A.	-3%
threo-12,13-DiHOME	80±13%	78±4%	61±3%	N.A.	N.A.	-4%
erythro-9,10-DiHODA	47±1%	37±2%	54±19%	N.A.	N.A.	1%
threo-9,10-DiHODA	44±15%	34±4%	51±2%	N.A.	N.A.	4%
9,10,13-TriHODE	104±15%	88±8%	80±11%	N.A.	N.A.	-5%
9,10,11-TriHODE	110±23%	82±6%	78±12%	N.A.	N.A.	-3%
9,12,13-TriHODE	103±12%	82±6%	74±9%	N.A.	N.A.	-2%
9,10,13-TriHOME	99±16%	91±6%	83±11%	N.A.	N.A.	-10%
9,10,11-TriHOME	87±9%	89±8%	87±11%	N.A.	N.A.	-2%
11,12,13-TriHOME	93±9%	93±7%	88±11%	N.A.	N.A.	-1%
9,12,13-TriHOME	95±14%	63±6%	45±18%	N.A.	N.A.	-12%
8-F_{1t}-$PhytoP_{DGLA}$	90±12%	87±8%	80±2%	N.A.	N.A.	-4%
15-F_{1t}-$PhytoP_{DGLA}$	86±9%	98±4%	81±2%	N.A.	N.A.	-11%
6-F_{2t}-$PhytoP_{SDA}$	83±22%	87±6%	79±3%	N.A.	N.A.	-11%
16-F_{2t}-$PhytoP_{SDA}$	N.D.	88±3%	79±1%	N.A.	N.A.	-8%
6-F_{1t}-$PhytoP_{GLA}$	95±15%	88±3%	83±6%	N.A.	N.A.	-8%
13-F_{1t}-$PhytoP_{GLA}$	N.D.	86±3%	83±2%	N.A.	N.A.	-8%
9-F_{1t}-$PhytoP_{ALA}$	106±5%	92±5%	83±6%	N.A.	N.A.	-7%
16-F_{1t}-$PhytoP_{ALA}$	92±12%	92±9%	83±5%	N.A.	N.A.	-3%
9-L_1-$PhytoP_{ALA}$	94±2%	95±14%	79±1%	N.A.	N.A.	-1%
16-B_1-$PhytoP_{ALA}$	95±2%	93±11%	78±1%	N.A.	N.A.	2%
ent-9-D_{1t}-$PhytoP_{ALA}$	105±13%	89±6%	83±4%	N.A.	N.A.	-1%
ent-9-12-*epi*-ST-Δ^{10}-13-PhytoF	89±14%	89±3%	84±5%	N.A.	N.A.	-2%
ent-16-13-*epi*-ST-Δ^{14}-9-PhytoF	N.D.	94±1%	85±5%	N.A.	N.A.	-6%
Average[c]	83±13%	75±5%	71±6%	N.A.	N.A.	8%
Range (Minimum - Maximum)[c]	13-110%	24-98%	26-90%	N.A.	N.A.	0-70%

[a] Errors are calculated as %CV on 6 analytical replicates.

[b] Matrix effect for ISTD determined as the percentage relative error between the signal for internal standards measured in solvent and the signal for the same compounds post-spiked in matrix extracts. Matrix effect for octadecanoids calculated as % relative error of the slopes of the solvent-matched calibration curve and the post-spiked calibration curve built on the same linear range.

[c] Average values and ranges do not include internal standards (ISTDs).

N.D.=not detected

N.A.=not applicable

Table S16. Analyte stability in the autosampler over 96 hours at 8℃

**Internal standard - Area**	**24h[a]**	**48h[a]**	**96h[a]**
13-KODE-d_3	106%	117%	128%
9-HODE-d_4	107%	117%	128%
cis-9,10-EpOME-d_4	109%	119%	131%
13-HODE-d_4	106%	118%	127%
cis-12,13-EpOME-d_4	108%	119%	128%
trans-9,10-Epoxt-13-HOME-d_5	103%	111%	112%
threo-9,10-DiHOME-d_4	105%	115%	127%
threo-12,13-DiHOME-d_4	104%	114%	127%
9,12,13-$^{13}C_3$-TriHOME	105%	119%	129%
16-F_{1t}-PhytoP-C_{19}	105%	122%	131%
**Average**	**106%**	**117%**	**127%**
**Analyte - Concentration**	**24h[a]**	**48h[a]**	**96h[a]**
9-KOTrE	99%	98%	99%
13-KOTrE	96%	95%	95%
9-KODE	101%	106%	95%
13-KODE	99%	110%	108%
cis-5,6-EpODE	74%	46%	17%
trans-5,6-EpODE	84%	73%	46%
cis-9,10-EpODA	91%	94%	97%
trans-9,10-EpODA	94%	98%	99%
cis-12,13-EpODE	100%	101%	100%
cis-9,10-EpOME	104%	107%	106%
cis-12,13-EpOME	97%	98%	96%
trans-12,13-EpOME	101%	100%	103%
9-HOTrE	106%	108%	107%
13-HOTrE	100%	106%	99%
13-HOTrE-γ	101%	95%	94%
9-HODE	102%	109%	106%
13-HODE	103%	112%	106%
12(*Z*)-10-KOME	99%	102%	100%
11(*E*)-10-KOME	98%	99%	95%
12(*Z*),15(*Z*)/11(*E*),15(*Z*)-10-KODE	98%	97%	101%
12(*Z*)-10-HOME	102%	98%	100%
11(*E*)-10-HOME	93%	100%	97%
12(*Z*),15(*Z*)/11(*E*),15(*Z*)-10-HODE	97%	97%	99%
9-oxo-*trans*-12,13-EpOME	105%	101%	103%
11-OH-*trans*-9,10-EpOME_1	106%	110%	125%
11-OH-*trans*-9,10-EpOME_2	100%	107%	122%
13-OH-*trans*-9,10-EpOME	99%	94%	96%
9-OH-*trans*-12,13-EpOME	82%	100%	116%
11-OH-*trans*-12,13-EpOME_1	102%	112%	111%
11-OH-*trans*-12,13-EpOME_2	102%	110%	110%
13-OH-12-KOME	101%	102%	103%

**Analyte - Concentration**	**24h[a]**	**48h[a]**	**96h[a]**
erythro-9,10-DiHODE	102%	103%	104%
threo-9,10-DiHODE	100%	103%	104%
erythro-12,13-DiHODE	100%	100%	97%
erythro-15,16-DiHODE	99%	99%	98%
erythro-9,10-DiHOME	97%	99%	96%
threo-9,10-DiHOME	103%	103%	107%
erythro-12,13-DiHOME	96%	97%	95%
threo-12,13-DiHOME	97%	99%	99%
erythro-9,10-DiHODA	96%	100%	93%
threo-9,10-DiHODA	97%	99%	93%
9,10,13-TriHODE	97%	97%	98%
9,10,11-TriHODE	97%	98%	97%
9,12,13-TriHODE	96%	96%	98%
9,10,13-TriHOME	95%	94%	99%
9,10,11-TriHOME	97%	95%	98%
11,12,13-TriHOME	99%	99%	99%
9,12,13-TriHOME	102%	102%	103%
**Average**	**98%**	**99%**	**99%**

[a] Values are percentage relative to time 0 (0 hours).

Table S17. Precision of extraction of QC samples analyzed by SFC-MS/MS

**Octadecanoid**	**Average concentration SFC (ng/mL)**[a]	**CV (% RSD)**
9-KOTrE	11.99 ± 0.73	6%
13-KOTrE	5.28 ± 1.97	37%
9-KODE	16.91 ± 2.20	13%
13-KODE	19.79 ± 0.86	4%
trans-5,6-EpODE_1	12.18 ± 1.77	15%
trans-5,6-EpODE_2	5.78 ± 1.13	20%
cis-5,6-EpODE_1	10.26 ± 1.55	15%
cis-5,6-EpODE _2	9.02 ± 1.55	17%
trans-9,10-EpODA_1	39.41 ± 4.54	12%
trans-9,10-EpODA_2	40.07 ± 5.63	14%
cis-9(*S*),10(*R*)-EpODA	372.10 ± 19.44	5%
cis-9(*R*),10(*S*)-EpODA	256.02 ± 33.39	13%
cis-12,13-EpODE	13.38 ± 3.37	25%
9(*S*)-HOTrE	7.90 ± 0.80	10%
13(*S*)-HOTrE	118.43 ± 8.43	7%
13(*S*)-HOTrE-γ	15.27 ± 1.23	8%
9(*S*)-HODE	20.21 ± 1.47	7%
9(*R*)-HODE	0.62 ± 0.07	11%
cis-9(*S*),10(*R*)-EpOME	10.42 ± 0.83	8%
cis-9(*R*),10(*S*)-EpOME	11.05 ± 1.96	18%
trans-9,10-EpOME_1	0.02 ± 0.01	22%
trans-9,10-EpOME_2	0.03 ± 0.01	23%
13(*S*)-HODE	21.46 ± 1.71	8%
13(*R*)-HODE	0.92 ± 0.06	7%
trans-12,13-EpOME	4.95 ± 0.74	15%
cis-12(*R*),13(*S*)-EpOME	9.43 ± 0.69	7%
cis-12(*S*),13(*R*)-EpOME	21.12 ± 1.17	6%
12(*Z*)-10-KOME	12.74 ± 1.83	14%
11(*R*)-10-KOME	18.15 ± 1.00	6%
12(*Z*),15(*Z*)-10-KODE	7.30 ± 0.66	9%
12(*Z*)-10(*R*)*-HOME	5.91 ± 0.60	10%
12(*Z*)-10(*S*)*-HOME	3.44 ± 0.32	9%
11(*R*)-10(*R*)*-HOME	5.32 ± 0.59	11%
11(*R*)-10(*S*)*-HOME	4.19 ± 0.45	11%
12(*Z*),15(*Z*)-10-(*R*)*-HODE	1.36 ± 0.09	7%
12(*Z*),15(*Z*)-10-(*S*)*-HODE	1.13 ± 0.10	9%
9-oxo-*trans*-12,13-EpOME _1	13.40 ± 2.04	15%
9-oxo-*trans*-12,13-EpOME _2	14.16 ± 2.64	19%
11-OH-*trans*-9,10-EpOME_01	21.14 ± 1.46	7%
11-OH-*trans*-9,10-EpOME_02	29.41 ± 2.05	7%
11-OH-*trans*-9,10-EpOME_03	18.71 ± 2.32	12%

Octadecanoid	Average concentration SFC (ng/mL)[a]	CV (% RSD)
11-OH-*trans*-9,10-EpOME_04	20.65 ± 1.49	7%
13-OH-*trans*-9,10-EpOME_01	13.59 ± 1.64	12%
13-OH-*trans*-9,10-EpOME_02	9.30 ± 0.63	7%
13-OH-*trans*-9,10-EpOME_03	24.06 ± 1.65	7%
9-OH-*trans*-12,13-EpOME_01	91.04 ± 7.16	8%
9-OH-*trans*-12,13-EpOME_02	94.89 ± 7.12	8%
9-OH-*trans*-12,13-EpOME_03	121.12 ± 6.79	6%
11-OH-*trans*-12,13-EpOME_01	61.88 ± 3.00	5%
11-OH-*trans*-12,13-EpOME_02	27.24 ± 2.58	9%
11-OH-*trans*-12,13-EpOME_03	27.70 ± 1.77	6%
11-OH-*trans*-12,13-EpOME_04	101.33 ± 5.43	5%
13-OH-12-KOME	28.17 ± 3.31	12%
threo-9(*R*),10(*R*)-DiHODE	0.36 ± 0.03	9%
threo-9(*S*),10(*S*)-DiHODE	0.49 ± 0.03	6%
erythro-9,10-DiHODE	3.36 ± 0.26	8%
erythro-12,13-DiHODE	1.53 ± 0.12	8%
erythro-15,16-DiHODE	6.22 ± 0.43	7%
threo-9(*R*),10(*R*)-DiHOME	6.94 ± 0.41	6%
threo-9(*S*),10(*S*)-DiHOME	8.86 ± 0.70	8%
erythro-9,10-DiHOME_1	8.29 ± 0.45	5%
erythro-9,10-DiHOME_2	7.71 ± 0.68	9%
*threo*_1+*threo*_2-+/*erythro*_1-12,13-DiHOME[#]	54.30 ± 2.42	4%
erythro-12,13-DiHOME_2	30.17 ± 2.65	9%
threo-9(*R*),10(*R*)-DiHODA*	81.39 ± 6.07	7%
*threo*_2-+*erythro*_1-9,10-DiHODA	124.03 ± 8.22	7%
erythro-9,10-DiHODA_2	37.20 ± 4.89	13%
9(*S*),10(*S*),13(*S*)-TriHODE	14.00 ± 0.96	7%
9(*S*),10(*S*),11(*R*)-TriHODE	132.22 ± 10.58	8%
9(*S*),12(*S*),13(*S*)-TriHODE	15.47 ± 1.62	10%
9(*S*),10(*S*),13(*S*)-TriHOME	23.61 ± 1.38	6%
9(*S*),10(*S*),11(*R*)-TriHOME	26.22 ± 1.99	8%
11(*R*),12(*S*),13(*S*)-TriHOME	48.83 ± 4.71	10%
9(*S*),12(*S*),13(*S*)-TriHOME	19.93 ± 1.17	6%
	**Average CV**	**10%**
	**Minimum CV**	**4%**
	**Maximum CV**	**37%**

* Order of elution not confirmed with enantiopure standard due to unavailability, but inferred by comparison with similar compounds under the same conditions.

[#] Eluted as three non-resolved peaks, integrated as a single peak.

[a] 12 analytical replicates in single injections over 151 biological sample injections; a QC replicate of human plasma was injected every 12-13 samples. Human reference plasma was acquired from Seralab (Haywards Heat, UK) with the following reported characteristics: origin, USA; sex, female; code, PK2F-123-F-28425; Batch No, T2012313.

Table S18. Precision of extraction of QC samples analyzed by LC-MS/MS

**Octadecanoid**	**Average concentration LC (ng/mL)[a]**	**CV (% RSD)**
9-KOTrE	15.20 ± 0.74	5%
13-KOTrE	5.11 ± 1.34	26%
9-KODE	22.14 ± 1.02	5%
13-KODE	16.04 ± 0.83	5%
cis-5,6-EpODE	4.15 ± 2.33	56%
trans-5,6-EpODE	12.21 ± 2.94	24%
cis-9,10-EpODA	1,023.03 ± 48.87	5%
trans-9,10-EpODA	160.15 ± 5.29	3%
cis-9,10-EpODE	0.29 ± 0.02	8%
cis-12,13-EpODE	12.60 ± 0.59	5%
cis-15,16-EpODE	5.24 ± 0.21	4%
cis-9,10-EpOME	7.37 ± 0.36	5%
trans-9,10-EpOME	1.87 ± 0.16	8%
cis-12,13-EpOME	36.79 ± 1.40	4%
trans-12,13-EpOME	4.08 ± 0.24	6%
9-HOTrE	5.61 ± 0.42	7%
13-HOTrE	51.40 ± 2.94	6%
13-HOTrE-γ	23.18 ± 1.81	8%
9-HODE	34.14 ± 1.91	6%
13-HODE	33.17 ± 1.60	5%
12(*Z*)-10-KOME	19.27 ± 1.19	6%
11(*E*)-10-KOME	24.69 ± 1.20	5%
12(*Z*),15(*Z*)/11(*E*),15(*Z*)-10-KODE	9.89 ± 0.59	6%
12(*Z*)-10-HOME	9.20 ± 0.66	7%
11(*E*)-10-HOME	12.49 ± 0.92	7%
12(*Z*),15(*Z*)/11(*E*),15(*Z*)-10-HODE	4.33 ± 0.26	6%
9-oxo-*trans*-12,13-EpOME	26.27 ± 1.64	6%
11-OH-*trans*-9,10-EpOME_1	45.87 ± 2.55	6%
11-OH-*trans*-9,10-EpOME_2	47.73 ± 2.29	5%
13-OH-*trans*-9,10-EpOME	54.59 ± 2.78	5%
9-OH-*trans*-12,13-EpOME	82.58 ± 6.01	7%
11-OH-*trans*-12,13-EpOME_1	72.74 ± 1.74	2%
11-OH-*trans*-12,13-EpOME_2	74.12 ± 1.88	3%
13-OH-12-KOME	31.29 ± 4.15	13%
8(*R*)-11(*S*)-DiHODE	21.96 ± 1.42	6%
erythro-9,10-DiHODE	3.74 ± 0.24	6%
threo-9,10-DiHODE	4.02 ± 0.27	7%
erythro-12,13-DiHODE	1.76 ± 0.09	5%
threo-12,13-DiHODE	0.32 ± 0.02	8%
erythro-15,16-DiHODE	8.10 ± 0.38	5%
threo-15,16-DiHODE	10.01 ± 0.34	3%
erythro-9,10-DiHOME	14.06 ± 0.71	5%
threo-9,10-DiHOME	16.30 ± 0.55	3%

Octadecanoid	Average concentration LC (ng/mL)[a]	CV (% RSD)
erythro-12,13-DiHOME	13.04 ± 0.66	5%
threo-12,13-DiHOME	16.53 ± 0.68	4%
erythro-9,10-DiHODA	87.25 ± 4.56	5%
threo-9,10-DiHODA	152.31 ± 9.29	6%
9,10,13-TriHODE	11.40 ± 1.06	9%
9,10,11-TriHODE	111.12 ± 3.75	3%
9,12,13-TriHODE	18.85 ± 1.76	9%
9,10,13-TriHOME	20.58 ± 1.03	5%
9,10,11-TriHOME	21.62 ± 0.81	4%
11,12,13-TriHOME	26.59 ± 1.11	4%
9,12,13-TriHOME	27.29 ± 1.87	7%
8-F_{1t}-$PhytoP_{DGLA}$	8.03 ± 0.49	6%
15-F_{1t}-$PhytoP_{DGLA}$	20.01 ± 0.78	4%
6-F_{2t}-$PhytoP_{SDA}$	5.73 ± 0.32	6%
16-F_{2t}-$PhytoP_{SDA}$	15.59 ± 1.07	7%
6-F_{1t}-$PhytoP_{GLA}$	6.55 ± 0.37	6%
13-F_{1t}-$PhytoP_{GLA}$	17.98 ± 1.04	6%
9-F_{1t}-$PhytoP_{ALA}$	5.03 ± 0.24	5%
16-F_{1t}-$PhytoP_{ALA}$	5.79 ± 0.36	6%
9-L_{1}-$PhytoP_{ALA}$	3.78 ± 0.26	7%
16-B_{1}-$PhytoP_{ALA}$	6.96 ± 0.39	6%
ent-9-D_{1t}-$PhytoP_{ALA}$	3.55 ± 0.22	6%
ent-9-12-*epi*-ST- Δ^{10}-13-PhytoF	13.29 ± 1.77	13%
ent-16-13-*epi*-ST- Δ^{14}-9-PhytoF	4.00 ± 0.43	11%
	**Average CV**	7%
	**Minimum CV**	2%
	**Maximum CV**	56%

[a] 12 analytical replicates in single injections over 151 biological sample injections; a QC replicate of human plasma was injected every 12-13 samples. Human reference plasma was acquired from Seralab (Haywards Heat, UK) with the following reported characteristics: origin, USA; sex, female; code, PK2F-123-F-28425; Batch No, T2012313.

Figure S9. Representative LC and SFC chromatogram of human and mouse plasma

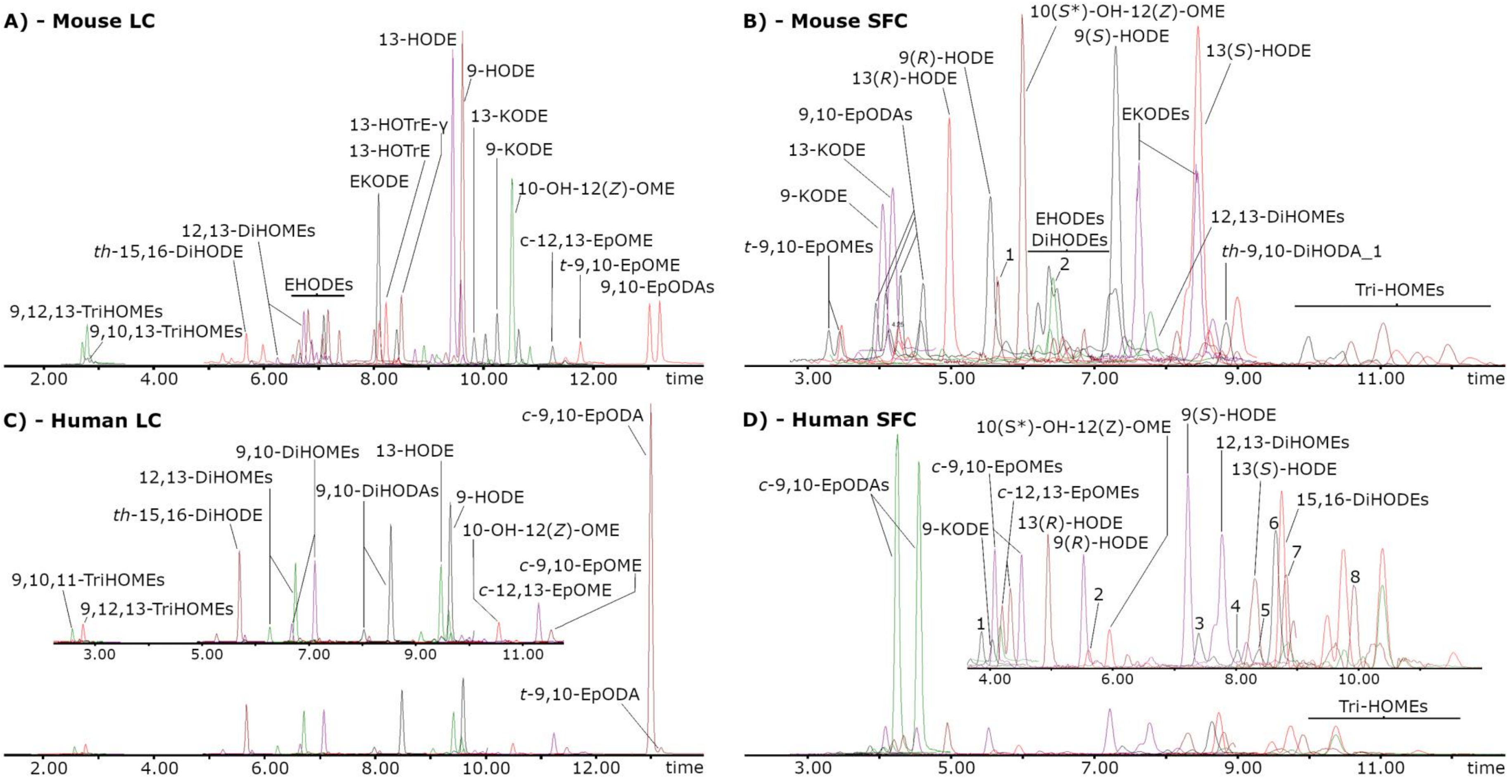

Figure S9. Representative LC and SFC chromatogram of human and mouse plasma. Chromatograms obtained by the analysis of mouse plasma by LC (**A**) and SFC (**B**) compared to those obtained by the analysis of reference human plasma by LC (**C**) and SFC (**D**). The insets in the human plasma chromatograms show the data with the exclusion of the high-abundant *cis*-9,10-EpODAs in order to enable improved visualization of all the detected species.

Abbreviations: epoxides: c- (*cis*-), t- (*trans*-); diols: e- (*erythro*-), th- (*threo*-). Full nomenclature of the detected species is reported in Table S1.

Legend for **panel B**: **1.** 12(*Z*)-10(*R*)*-HOME; **2.** 13(*S*)-HOTrE

Legend for **panel D**: **1.** *cis*-15,16-EpODE; **2.** 12(*Z*)-10(*R*)*-HOME; **3.** *threo*-9,10-DiHOME_1; **4.** *erythro*-9,10-DiHOME_1; **5.** *erythro*-9,10-DiHOME_2; **6.** *threo*-9,10-DiHOME_2; **7.** *threo*-9,10-DiHODA_1; **8.** *threo*-9,10-DiHODA_2+*erythro*-9,10-DiHODA_1

* Order of elution not confirmed with enantiopure standard due to unavailability, but inferred by comparison with similar compounds under the same conditions.

Note that the retention times on the SFC chromatograms may present minor shifts from the values reported in Table S5 due to the fact that these chromatograms were acquired on a different AMY-1 column. All shifts were within 0.2 min except for 13(*S*)-HODE, which shifted 0.45 min.

References

(1) Kolmert, J.; Fauland, A.; Fuchs, D.; Säfholm, J.; Gómez, C.; Adner, M.; Dahlén, S.-E.; Wheelock, C. E. Lipid Mediator Quantification in Isolated Human and Guinea Pig Airways: An Expanded Approach for Respiratory Research. *Anal Chem* **2018**, *90* (17), 10239–10248. https://doi.org/10.1021/acs.analchem.8b01651.

(2) Hamberg, M.; Fahlstadius, P. Allene Oxide Cyclase: A New Enzyme in Plant Lipid Metabolism. *Arch Biochem Biophys* **1990**, *276* (2), 518–526. https://doi.org/10.1016/0003-9861(90)90753-L.

(3) Ramsden, C. E.; Domenichiello, A. F.; Yuan, Z.-X.; Sapio, M. R.; Keyes, G. S.; Mishra, S. K.; Gross, J. R.; Majchrzak-Hong, S.; Zamora, D.; Horowitz, M. S.; Davis, J. M.; Sorokin, A. V.; Dey, A.; LaPaglia, D. M.; Wheeler, J. J.; Vasko, M. R.; Mehta, N. N.; Mannes, A. J.; Iadarola, M. J. A Systems Approach for Discovering Linoleic Acid Derivatives That Potentially Mediate Pain and Itch. *Sci Signal* **2017**, *10* (493), eaal5241. https://doi.org/10.1126/scisignal.aal5241.

(4) Fuchs, D.; Hamberg, M.; Sköld, C. M.; Wheelock, Å. M.; Wheelock, C. E. An LC-MS/MS Workflow to Characterize 16 Regio- and Stereoisomeric Trihydroxyoctadecenoic Acids. *J Lipid Res* **2018**, *59* (10), 2025–2033. https://doi.org/10.1194/jlr.D087429.

(5) Hamberg, M. Regio- and Stereochemical Analysis of Trihydroxyoctadecenoic Acids Derived from Linoleic Acid 9- and 13-Hydroperoxides. *Lipids* **1991**, *26* (6), 407–415. https://doi.org/10.1007/BF02536065.

(6) Hamberg, M. Fatty Acid Hydroperoxide Isomerase InSaprolegnia Parasitica: Structural Studies of Epoxy Alcohols Formed from Isomeric Hydroperoxyoctadecadienoates. *Lipids* **1989**, *24* (4), 249–255. https://doi.org/10.1007/BF02535158.

(7) Guy, A.; Flanagan, S.; Durand, T.; Oger, C.; Galano, J.-M. Facile Synthesis of Cyclopentenone B1- and L1-Type Phytoprostanes. *Front Chem* **2015**, *3*. https://doi.org/10.3389/fchem.2015.00041.

(8) El Fangour, S.; Guy, A.; Despres, V.; Vidal, J.-P.; Rossi, J.-C.; Durand, T. Total Synthesis of the Eight Diastereomers of the Syn-Anti-Syn Phytoprostanes F1 Types I and II. *J Org Chem* **2004**, *69* (7), 2498–2503. https://doi.org/10.1021/jo035638i.

(9) Cuyamendous, C.; Leung, K. S.; Durand, T.; Lee, J. C.-Y.; Oger, C.; Galano, J.-M. Synthesis and Discovery of Phytofurans: Metabolites of α-Linolenic Acid Peroxidation. *Chem Commun* **2015**, *51* (86), 15696–15699. https://doi.org/10.1039/C5CC05736A.

(10) Kai, K.; Takeuchi, J.; Kataoka, T.; Yokoyama, M.; Watanabe, N. Structure–Activity Relationship Study of Flowering-Inducer FN against Lemna Paucicostata. *Tetrahedron* **2008**, *64* (28), 6760–6769. https://doi.org/10.1016/j.tet.2008.04.115.

(11) Pivnitsky K.K.; Lapitskaya L.A.; Vasiljeva L.L., last. A Chemoselective Synthesis of Functionalized 1,4-Alkadiynes (Skipped Diacetyelenes). *Synthesis* **1993**, 65–66.

(12) Hamberg, M. Vanadium-Catalyzed Transformation of 13(S)-Hydroperoxy-9(Z),11(E)-Octadecadienoic Acid: Structural Studies on Epoxy Alcohols and Trihydroxy Acids. *Chem Phys Lipids* **1987**, *43*, 55–67.

(13) Smith, C. R.; Bagby, M. O.; Lohmar, R. L.; Glass, C. A.; Wolff, I. A. The Epoxy Acids of Chrysanthemum Coronarium and Clarkia Elegans Seed Oils. *J. Org. Chem.* **1960**, *25* (2), 218–222. https://doi.org/10.1021/jo01072a019.

(14) Laboureur, L.; Ollero, M.; Touboul, D. Lipidomics by Supercritical Fluid Chromatography. *IJMS* **2015**, *16* (12), 13868–13884. https://doi.org/10.3390/ijms160613868.

(15) Jonasdottir, H. S.; Brouwers, H.; Toes, R. E. M.; Ioan-Facsinay, A.; Giera, M. Effects of Anticoagulants and Storage Conditions on Clinical Oxylipid Levels in Human Plasma.

BBA-Mol Cell Biol L **2018**, *1863* (12), 1511–1522. https://doi.org/10.1016/j.bbalip.2018.10.003.
(16) Wolfer, A. M.; Gaudin, M.; Taylor-Robinson, S. D.; Holmes, E.; Nicholson, J. K. Development and Validation of a High-Throughput Ultrahigh-Performance Liquid Chromatography–Mass Spectrometry Approach for Screening of Oxylipins and Their Precursors. *Anal Chem* **2015**, *87* (23), 11721–11731. https://doi.org/10.1021/acs.analchem.5b02794.
(17) Yuan, Z.-X.; Majchrzak-Hong, S.; Keyes, G. S.; Iadarola, M. J.; Mannes, A. J.; Ramsden, C. E. Lipidomic Profiling of Targeted Oxylipins with Ultra-Performance Liquid Chromatography-Tandem Mass Spectrometry. *Anal Bioanal Chem* **2018**, *410* (23), 6009–6029. https://doi.org/10.1007/s00216-018-1222-4.
(18) Yang, J.; Schmelzer, K.; Georgi, K.; Hammock, B. D. Quantitative Profiling Method for Oxylipin Metabolome by Liquid Chromatography Electrospray Ionization Tandem Mass Spectrometry. *Anal Chem* **2009**, *81* (19), 8085–8093. https://doi.org/10.1021/ac901282n.
(19) Psychogios, N.; Hau, D. D.; Peng, J.; Guo, A. C.; Mandal, R.; Bouatra, S.; Sinelnikov, I.; Krishnamurthy, R.; Eisner, R.; Gautam, B.; Young, N.; Xia, J.; Knox, C.; Dong, E.; Huang, P.; Hollander, Z.; Pedersen, T. L.; Smith, S. R.; Bamforth, F.; Greiner, R.; McManus, B.; Newman, J. W.; Goodfriend, T.; Wishart, D. S. The Human Serum Metabolome. *PLoS ONE* **2011**, *6* (2), e16957. https://doi.org/10.1371/journal.pone.0016957.
